# Microtubule posttranslational modifications provide unique recognition patterns for associated proteins

**DOI:** 10.1038/s44318-026-00908-8

**Published:** 2026-09-12

**Authors:** Arya Krishnan, Eva Desvigne-Hansch, Veronique Henriot, Alica Dodoková, Sinda Khanfir, Mariya Genova, Lenka Grycova, Maria M Magiera, Marcus Braun, Zdenek Lansky, Carsten Janke

**Affiliations:** 1 Institut Curie, Université PSL, CNRS UMR3348, Inserm U1368, Genome, RNA and Cancer, Orsay, France; 2 Université Paris-Saclay, CNRS UMR3348, Inserm U1368, Genome, RNA and Cancer, Orsay, France; 3 Institute of Biotechnology, Czech Academy of Sciences, BIOCEV, Vestec, Czech Republic; 4 Department of Cell Biology, Faculty of Science, Charles University, Prague, Czech Republic

**Keywords:** Cell Adhesion, Polarity & Cytoskeleton, Post-translational Modifications & Proteolysis

## Abstract

Microtubules are key components of the eukaryotic cytoskeleton involved in vital functions in virtually every cell. Among the emerging molecular mechanisms to adapt microtubules to their diverse functions is the biochemical diversification of tubulin molecules by posttranslational modifications (PTMs) and differential gene expression, a concept known as the ‘tubulin code’. A key question remains whether the tubulin code has the potential to selectively control microtubule interactions of different microtubule-associated proteins (MAPs) to act as a specific signalling system. To answer this question, we used a medium-throughput in vitro approach to screen 46 proteins for their binding preferences to microtubules with altered PTM or isotype composition. We demonstrate that subsets of these MAPs have unique sensitivities to PTMs, while other proteins are not affected. As a result, PTMs, or combinations of them, differentially attract or repulse individual MAPs to microtubules. Our findings offer mechanistic proof for a key hypothesis of the tubulin code—the capacity to selectively and differentially regulate MAP–microtubule interactions.

## Synopsis

Biochemical diversity of tubulin, generated by its posttranslational modifications or tubulin isotypes, endows microtubules with distinct properties and functions, a concept known as the ‘tubulin code’. This study reveals differential regulation of a broad variety of microtubule-associated proteins (MAPs) by the tubulin code.

- A medium-throughput screen using lysates of mammalian cells expressing fluorescently tagged MAPs allows quantification of the impact of tubulin posttranslational modifications and isotypes on MAP-microtubule interactions.
- Some MAPs show a specific recognition pattern for one or several tubulin modifications, while others are insensitive.
- Differential splicing of the mitochondria adaptor protein syntaphilin changes its sensitivity to tubulin polyglutamylation.
- Members of the conserved echinoderm microtubule-associated protein-like (EML) family differ in their sensitivity to tubulin tyrosination and polyglutamylation, illustrating evolutionary diversification of tubulin-code readers.

## Abstract (web summary)

A screen encompassing 46 microtubule-associated proteins reveals their distinct affinities to tubulin posttranslational modifications.

## Graphical abstract

**Figure:**
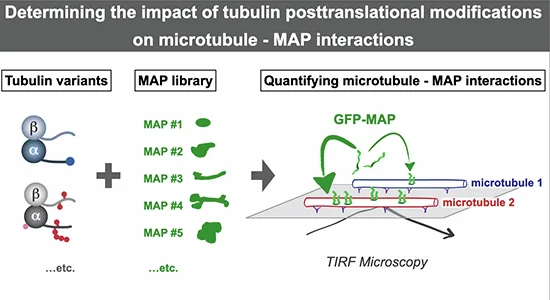

## Introduction

Microtubules play essential roles in every eukaryotic cell. How these functions are coordinated between different microtubule populations in the cell, or how they are fine-tuned to adapt to changing physiological requirements, are fascinating questions that have, until today, not been fully answered. The discovery of posttranslational modifications (PTMs) on tubulin led already in the 1970s to the hypothesis that biochemical diversification of tubulin might influence microtubule functions (Fulton and Simpson, 1976). However, solid proof of this concept, later baptised as the ‘tubulin code’ (Verhey and Gaertig, 2007), was difficult to obtain.

The tubulin code hypothesis stipulates that specific signatures on tubulin molecules, either encoded in different tubulin genes that give rise to α- and β-tubulin isotypes, or posttranslationally generated by tubulin PTMs (Janke and Magiera, 2020; McKenna et al, 2023), provide unique signals that can be sensed and interpreted in a specific manner by ‘readers’—i.e. microtubule-interacting proteins. Indeed, studies in cells found effects of tubulin PTMs or isotypes on the distribution of several microtubule-associated proteins (MAPs; e.g. Arru et al, 2021; Backer et al, 2012; Mohan et al, 2019; Peris et al, 2006), on cargo transport in neurons (Bodakuntla et al, 2020; Gilmore-Hall et al, 2019; Konietzny et al, 2024; Konishi and Setou, 2009; Magiera et al, 2018), or on ciliary and flagellar beating (Gadadhar et al, 2021; Ikegami et al, 2010; Pathak et al, 2011; Suryavanshi et al, 2010). This provided exciting insights into the potential of the tubulin code to regulate the function of the microtubule cytoskeleton within cells, which was further bolstered by observations of subsets of microtubules with unique PTMs in different cell types (Katrukha et al, 2021; Mohan et al, 2019; Orbach and Howard, 2019).

Direct in vitro measurements have demonstrated that tubulin PTMs can directly control microtubule-interacting proteins. For example, the activity of the microtubule-severing enzymes spastin and katanin is regulated by different types (Genova et al, 2023; Lacroix et al, 2010; Szczesna et al, 2022) or degrees (Valenstein and Roll-Mecak, 2016) of the PTM polyglutamylation. Molecular motors from the kinesin family display selective sensitivities to the type and degree of polyglutamylation on the microtubules they use as tracks (Genova et al, 2023; Lessard et al, 2019; Sirajuddin et al, 2014). Cytoplasmic dynein, on the other hand, requires tyrosinated α-tubulin for initial binding to microtubules, but can then walk with similar efficiency on microtubules with different PTM decoration (McKenney et al, 2016). Among the large family of MAPs, the Alzheimer-related MAP Tau, as well as centriole and spindle-associated protein (CSAP), were shown to bind more strongly to microtubules if they are polyglutamylated (Backer et al, 2012; Genova et al, 2023), while CLIP170 requires tyrosinated α-tubulin to track the plus-ends of microtubules (Bieling et al, 2008; Chen et al, 2021). Within the echinoderm microtubule-associated protein-like (EML) family, it was shown that only a short isoform of EML2 is sensitive to tubulin tyrosination, while other family members could not sense this PTM (Hotta et al, 2022).

While these striking examples have provided evidence that tubulin PTMs can control events at the cellular and molecular levels, the language of the tubulin code has so far not been deciphered. To do so requires demonstrating that individual tubulin PTMs, or combinations of them, are selectively and differentially read by effectors that interact with microtubules, while others are not affected.

Here, we put in place an approach to quantify how a representative panel of tubulin PTMs affects the interactions of a broad selection of MAPs with microtubules in vitro under directly comparable experimental conditions. We tested 46 arbitrarily chosen proteins described as structural MAPs - proteins that bind microtubules without enzymatic or motile function (Bodakuntla et al, 2019) and measured their interactions with microtubules purified from native sources that selectively lack tubulin PTMs (Genova et al, 2023). We found that about half of the tested proteins that showed robust enough binding in our assay are sensitive to tubulin composition. We then refined our analyses to single PTMs, which allowed us to determine a precise ‘PTM signature’ for every single MAP. Our work provides evidence for the existence of a mechanistic framework for the tubulin code: that different tubulin PTMs, alone or in combination, create signals that are differentially recognised, or ‘read’, by distinct microtubule interactors.

## Results

### Setting up a pipeline for medium-throughput analyses

Microtubules interact with a wide variety of proteins, and additional MAPs are discovered regularly. Despite this wealth of information, only a few MAPs have been characterised in detail (Bodakuntla et al, 2019). The restriction to a few selected MAPs is related to scientific interest, but all too often also to technical limitations. For instance, in vitro reconstitution assays are typically performed with purified proteins, and purification of proteins can require cumbersome and time-consuming optimisation, making it impossible to screen large numbers of proteins. To be able to broaden in vitro analyses to a wider collection of proteins, we used lysates of mammalian cells overexpressing the proteins of interest tagged with a fluorescent protein. We selected 46 proteins (Appendix Table S1) from the literature which are either bona fide microtubule binders with strong experimental evidence, or have been proposed to be MAPs (Bodakuntla et al, 2019). Most of the MAPs tested here have previously been confirmed as microtubule binders (Jijumon et al, 2022). We expressed GFP-labelled constructs of these MAPs (Appendix Table S1) in HEK-293 cells and prepared cytosolic lysates.

The second component of our assay are microtubules with well-defined tubulin PTMs. All tubulins used in this study are purified from mouse brain tissue or HeLa cells through repeated cycles of polymerisation and depolymerisation (Souphron et al, 2019), a method that has been a standard in the field for decades (Vallee, 1986). Tubulin from both native sources is a mixture of tubulin isotypes. However, a unique isotype that is present in brain tissue but not in wild-type HeLa cells is β3-tubulin (TubB3; Denoulet et al, 1986). The primary amino acid sequence of β3-tubulin shows some unique differences from other β-tubulin isotypes. Incorporation of β3-tubulin into microtubules can change their properties (Ti et al, 2018) as well as their interactions with other proteins (Sirajuddin et al, 2014). Another difference between brain tubulin and HeLa tubulin is the strong enrichment of the well-characterised PTMs in brain tubulin, whereas none of them is present on HeLa tubulin (Barisic et al, 2015). To determine the role of single tubulin variants, we used the following approaches: For polyglutamylation, we purified tubulin from mouse brains of double-knockout mice lacking Ttll1 and Ttll7, the two major polyglutamylases in the brain (Bodakuntla et al, 2021; Ikegami et al, 2006; Janke et al, 2005). To study the role of acetylation and β3-tubulin, we purified tubulin from *Atat1*-KO brains that does not contain acetylation (Kalebic et al, 2013), and from mice lacking the β3-tubulin (TubB3) isotype (Latremoliere et al, 2018), respectively. To study the role of detyrosination, brain tubulin, which is a mixture of tyrosinated, detyrosinated, and ∆2-tubulin (Paturle-Lafanechere et al, 1991), is rather cumbersome to separate into these different PTM variants (Giraudel et al, 1998; Lafanechere and Job, 2011). We thus used HeLa tubulin, which is naturally fully tyrosinated, and converted it to detyrosinated tubulin by treatment with carboxypeptidase A (Raybin and Flavin, 1977), and established a method known to entirely remove tyrosine from α-tubulin.

**Figure 1:**
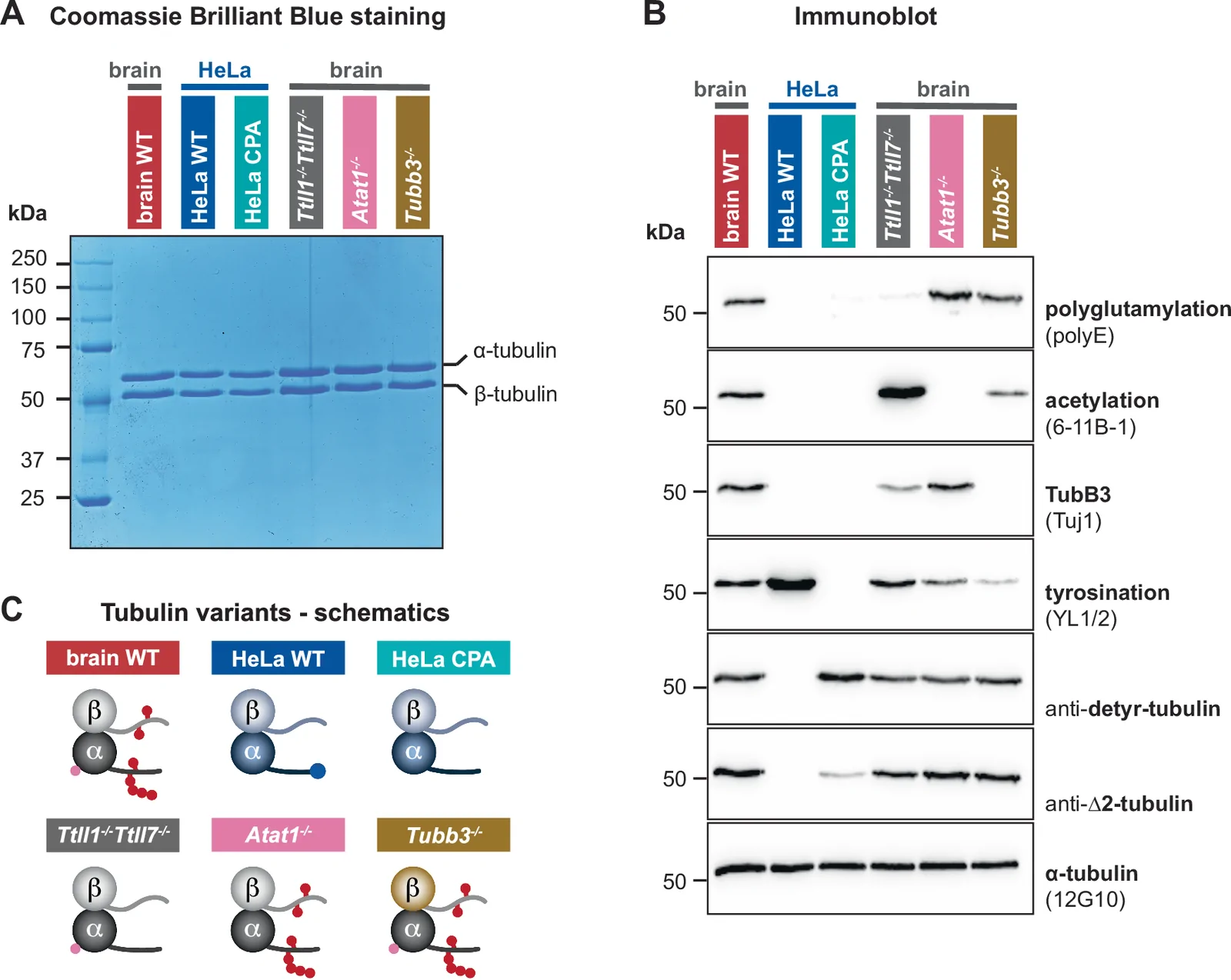
Characterisation of purified tubulin. (**A**) Two micrograms of purified tubulin were analysed on SDS-PAGE gels optimised for the resolution of α- and β-tubulin (Lacroix and Janke, 2011; Souphron et al, 2019). The gels were stained with Coomassie Brilliant Blue, revealing distinct bands corresponding to α- and β-tubulin. Note that no additional proteins are detected in the purified samples. (**B**) Immunoblot analyses of purified tubulin with PTM-specific antibodies (Appendix Table S3). Polyglutamylation was detected with polyE antibody, acetylation with 6-11B-1, β3-tubulin (TubB3) was detected using the Tuj1 antibody, tyrosination of α-tubulin with YL1/2, detyrosination and ∆2-tubulin with polyclonal antibodies specific to these two PTM variants (Paturle-Lafanechere et al, 1994). (**C**) Schematic representation of the different tubulin variants used in the current study. Source data are available online for this figure.

SDS-PAGE analysis showed high purity of the tubulin samples, and no visible differences between the different tubulin variants (Fig. 1A). Immunoblot analyses (Fig. 1B) confirmed that tubulin from *Ttll1*^*−/−*^*Ttll7*^*−/−*^ brain lacked polyE signals, confirming the absence of α- and β-tubulin polyglutamylation. The absence of polyglutamylation was further confirmed by quantitative top-down proteomics analysis of the samples in another study, showing a drop of average polyglutamylation from ~1.5 glutamates in wild-type α-tubulin to ~0.2 in *Ttll1*^*−/−*^*Ttll7*^*−/−*^ tubulin, and from ~1.7 on β-tubulin to ~0.01 in *Ttll1*^*−/−*^*Ttll7*^*−/−*^ tubulin (Gant et al, 2026). *Atat1*^*−/−*^ tubulin specifically lacked acetylation, and no β3-tubulin was detected in tubulin purified from *Tubb3*^*−/−*^ mice. We further confirmed that tubulin purified from HeLa cells does not contain any of the above-tested PTMs, as well as no β3-tubulin. After treatment with carboxypeptidase A, tyrosination of HeLa tubulin is entirely replaced by detyrosination (Fig. 1B,C). The established specificity of the antibodies for tyrosinated and detyrosinated tubulin makes this result unambiguous.

**Figure 2:**
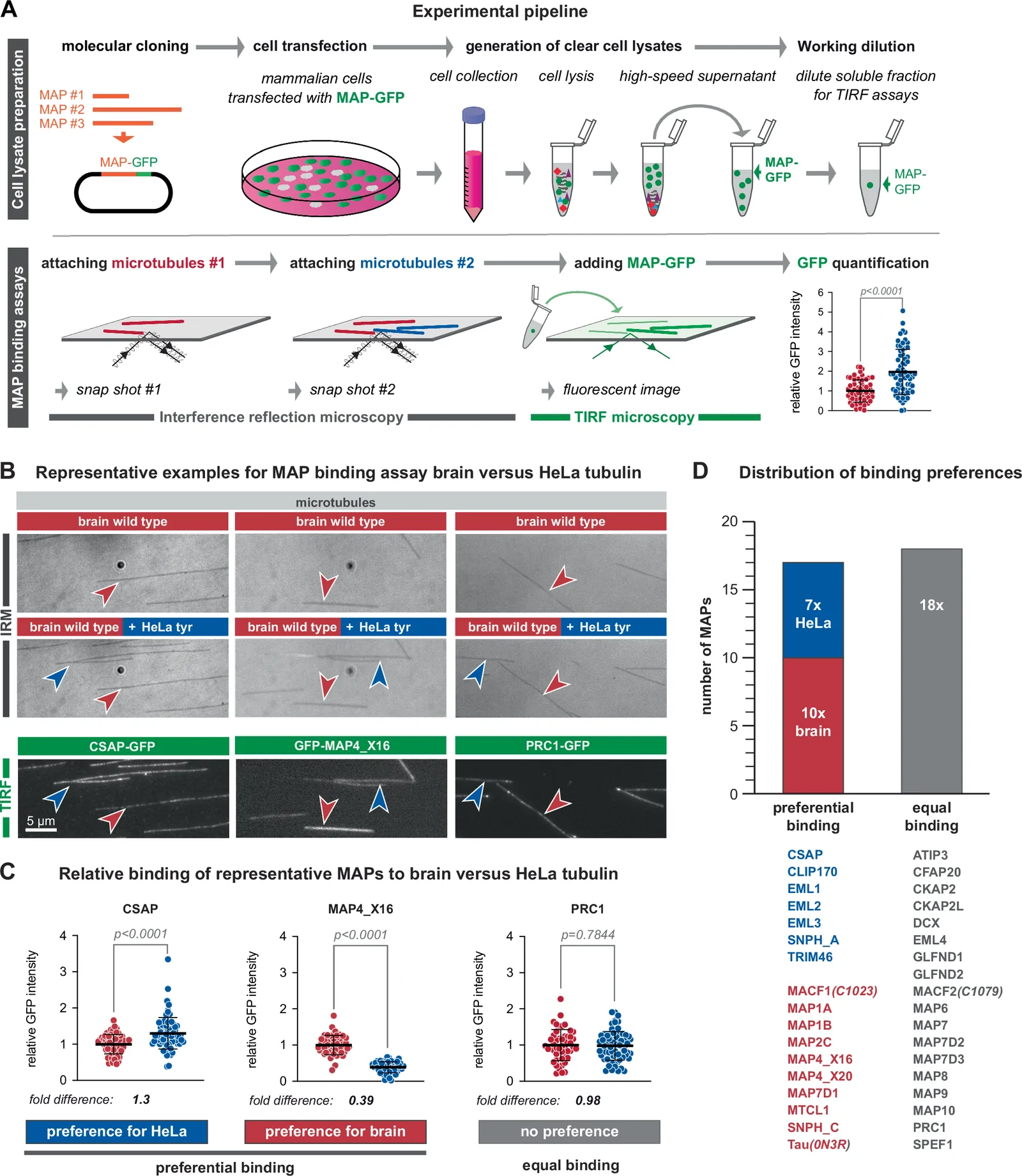
Distribution of MAP binding preferences between microtubules polymerised from brain versus HeLa tubulin. (**A**) Schematic representation of the experimental pipeline used in this study. Lysates from mammalian cells were generated similarly to our previously described method (Jijumon et al, 2022; Jijumon et al, 2024) but were highly diluted in buffer for the assays. TIRF assays with different microtubule variants were performed similar to previous work (Genova et al, 2023) but with diluted cell lysates instead of purified proteins (with the exception of purified EML proteins). (**B**) Representative examples of three MAPs with different binding preferences in the initial screen. IRM images showing snapshots taken after sequential addition of brain followed by HeLa microtubules, alongside corresponding TIRF images illustrating MAP binding. CSAP preferentially binds HeLa microtubules (image from Appendix Fig. S6), MAP4_X16 preferentially binds brain microtubules (image from Appendix Fig. S15), while PRC1 shows no binding preference (image from Appendix Fig. S4). (**C**) Quantitative analysis of CSAP, MAP4_X16, and PRC1 binding to microtubules (plots from Appendix Figs. S4, S6, and S15). Fluorescence intensities associated with HeLa microtubules were normalised to those of brain microtubules. Each data point represents an individual microtubule. Bars indicate mean ± standard deviation. Statistical significance was determined using unpaired *t* tests; *P* values are shown above each comparison, and fold changes are indicated below. (**D**) Graphical representation of the distribution of MAPs exhibiting preferential binding to either brain or HeLa microtubules, as well as MAPs showing no binding preference in the initial screen. All detailed analyses are shown in Appendix Figs. S2–S24.

This panel of tubulins enabled us to systematically test how MAPs interact with microtubules with distinct PTM or isotype patterns. Microtubule-binding assays were set up with microtubules either polymerised in the presence of GTP and then stabilised with Taxol (Schiff et al, 1979), or directly grown and stabilised with the GTP analogue GMPCPP (Hyman et al, 1992). For each experiment, microtubules of two different PTM or isotype compositions were sequentially flown into a microscopy chamber, where they were anchored by tubulin antibodies on passivised glass coverslips. To differentiate between the two microtubule species, interference reflection microscopy (IRM; Mahamdeh et al, 2018) snapshots were taken at each step (Fig. 2A,B). We then introduced the diluted cell lysates containing the overexpressed MAP-GFP and quantified the fluorescence intensities on the two different types of microtubules with total internal reflection (TIRF) microscopy (Fig. 2A,B). Given that several tested MAPs displayed the same binding preferences over a range of concentrations spanning an order of magnitude (Appendix Fig. S1), all MAP-GFP lysates were diluted such that the MAP-GFP binding to the microtubule was not saturated (Fig. 2C).

### Determination of the binding preferences of MAPs for differentially modified microtubules

To determine which MAPs can sense differences in tubulin composition, we performed an initial screen in which we compared the binding of all GFP-MAPs with brain versus HeLa microtubules. Overall, of the 46 proteins we tested here (Appendix Table S1; Fig. 2A), 35 showed a clear binding to microtubules in our assays. Each protein was tested in three sets of experiments (Appendix Figs. S2–S24), each performed with independently purified tubulins and cell lysates prepared separately in two independent batches (representative examples of the assay are shown in Fig. 2B,C). The mean values determined in the three experiments were averaged, and fold changes were determined. Here we considered fold changes ≥1.2 or ≤0.8 as differential binding, thus 17 out of 35, i.e. about 50% of the tested proteins, showed clear differential binding (Fig. 2D; Appendix Figs. S2–4, S10, S17 and S18 —no differential binding; Appendix Figs. S5–S9, S11–S16 and S19–S24 differential binding). Of those, 10 preferentially bound to brain microtubules and 7 showed preferences to HeLa microtubules (Fig. 2D). This result suggested that the different PTM and isotype signatures of these two fundamentally different types of microtubules can selectively attract or repel certain MAPs, while other MAPs are insensitive to these signatures.

**Figure 3:**
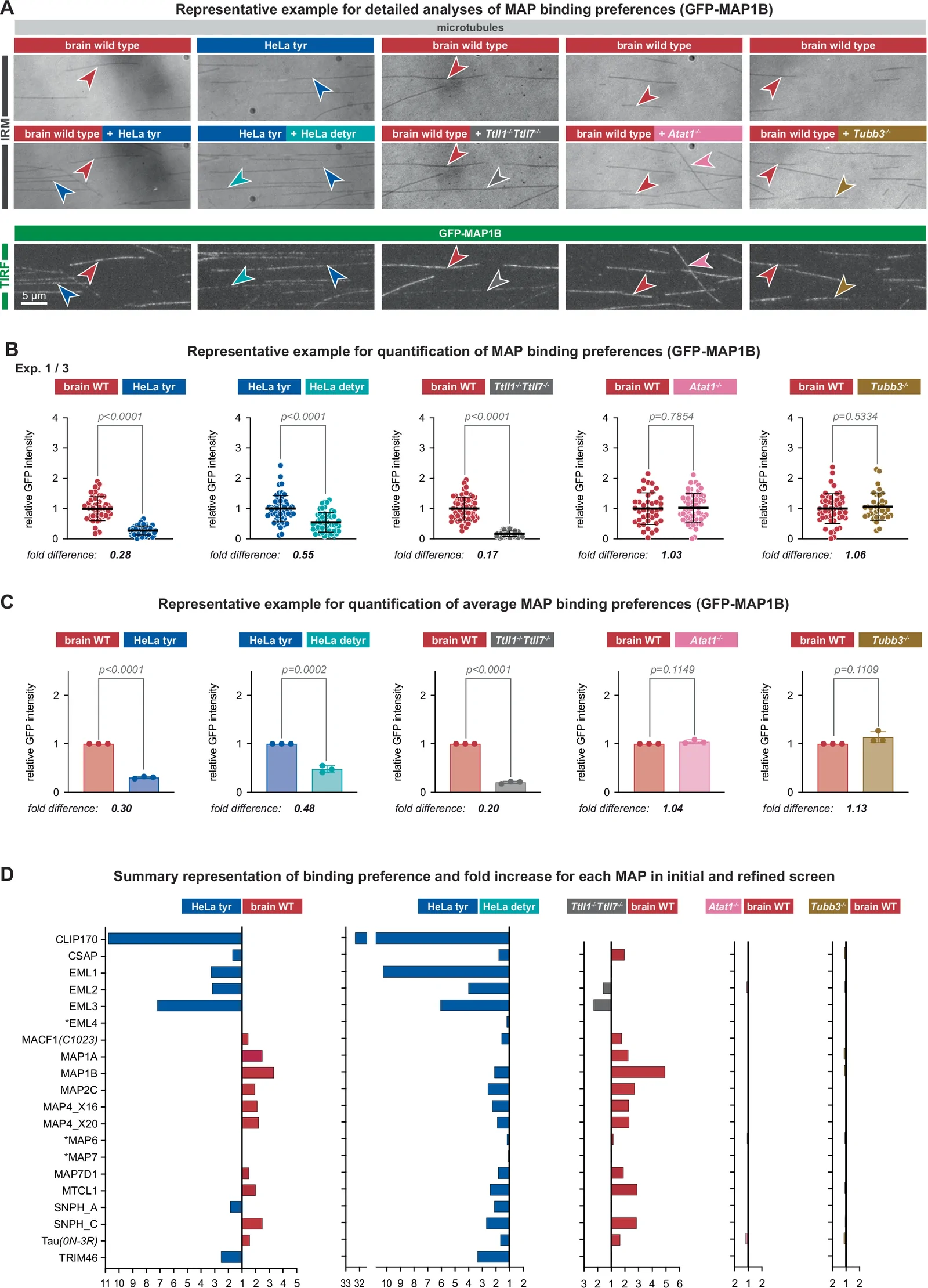
Binding preferences of MAPs to microtubules with distinct PTMs. (**A**) Representative TIRF microscopy images showing the binding of MAP1B (images from Appendix Fig. S13A) from cell lysates to Taxol-stabilised microtubules with distinct PTM patterns, sequentially introduced and visualised by IRM. (**B**) Quantification of MAP1B binding intensities (one experiment out of three replicates shown from Appendix Fig. S13B) shown directly below each corresponding TIRF image, with fluorescence values normalised to wild-type microtubules (either wild-type brain or wild-type HeLa). Each data point represents a single microtubule. Bars represent mean ± standard deviation. (**C**) Average MAP1B binding to different microtubule types (plots from Appendix Fig. S13C). Each data point represents the mean value from one independent assay. (**A**–**C**) Red indicates wild-type brain microtubules; dark blue, wild-type HeLa; light blue, detyrosinated HeLa; grey, *Ttll1*^*−/−*^*Ttll7*^*−/−*^ pink, *Atat1*^*−/−*^; and golden brown, *Tubb3*^*−/−*^ mouse brain-derived microtubules. (**B**, **C**) Statistical significance was determined using unpaired *t* tests; *P* values are shown above each comparison, and fold differences are indicated below. All experiments were performed in triplicate using independently purified tubulin and MAP1B lysates prepared from three separate cell batches. (**D**) Graphical summary of binding preferences of 17 MAPs identified as sensitive to specific tubulin PTMs, as well as *EML4, *MAP6 and *MAP7 as MAPs that did not show differences in the initial screen, are included. The *X* axis shows average binding preferences and fold differences from three independent experiments (Appendix Figs. S5–S24). Note that for CLIP170, no data were obtained for brain *Ttll1*^*−/−*^*Ttll7*^*−/−*^, *Atat1*^*−/−*^ and *Tubb3*^*−/−*^ microtubules (Appendix Fig. S5).

To determine which tubulin PTM or isotype is responsible for the differential binding between brain and HeLa microtubules of the 17 MAPs (Fig. 2D), we compared the binding of each of them between microtubule subtypes differing in only one PTM or tubulin isotype. We also included 3 MAPs that did not show differential binding in the initial screen (EML4, Appendix Fig. S10; MAP6, Appendix Fig. S17; MAP7, Appendix Fig. S18). Strikingly, we found that the mechanisms underlying the observed preferences to either brain or HeLa tubulin are highly divergent for different MAPs. The assays for each MAP were performed with independently purified tubulin batches and cell lysates prepared separately in three independent batches. The data for each set of experiments are shown separately, and the means of these subsets are then directly compared. For all MAPs tested here, we found a high degree of reproducibility among the single sets of experiments (Appendix Figs. S5–S24; MAP1B shown as a representative example in Fig. 3A–C).

Proteins such as EML1 (Appendix Fig. S7), SNPH_A (Appendix Fig. S21), and TRIM46 (Appendix Fig. S24) are predominantly, if not exclusively, affected by the state of tyrosination of microtubules, as they only show differences in binding between HeLa and detyr-HeLa microtubules (Fig. 3D), but for none of the other microtubule variants. This finding straight-forwardly explains their preferential association with HeLa over brain microtubules in the initial screen, given that brain tubulin contains less than 25% of tyrosinated tubulin (Barra et al, 1980; Paturle et al, 1989; Raybin and Flavin, 1977), while HeLa tubulin is almost fully tyrosinated (Fig. 1B). A selective sensitivity for a single PTM was also observed for MAP1A, which showed stronger binding to polyglutamylated versus non-glutamylated microtubules, but no discernible sensitivity to the tyrosination status (Fig. 3D; Appendix Fig. S12). All other MAPs showed sensitivity to two of the tested PTMs, predominantly tyrosination and polyglutamylation. By contrast, neither the absence of tubulin acetylation nor of β3-tubulin had pronounced effects on the microtubule interactions of most MAPs investigated here (Fig. 3D).

When tested for single PTM preferences, EML2 (Appendix Fig. S8) and EML3 (Appendix Fig. S9) bound preferentially to tyrosinated over detyrosinated HeLa, and to low-glutamylated over polyglutamylated brain microtubules, which makes their preference for HeLa over brain microtubules obvious: HeLa microtubules are tyrosinated and non-glutamylated, while brain microtubules have low tyrosination levels and are enriched in polyglutamylation. This, however, was rather unique, as other MAPs showed preferential binding to both tyrosinated and polyglutamylated microtubules (Fig. 3D: CSAP, MACF1*(C1023)*, MAP1B, MAP2, MAP4 isoforms, MAP7D1, MTCL1, SNPH_C, Tau*(0N-3R)*). As polyglutamylation is present predominantly in brain tubulin, and tyrosination is more abundant in HeLa tubulin, the overall impact of a single PTM on the binding preferences in this group of MAPs was therefore not inferable from the initial screen. Similarly, knowing binding preferences of MAPs for a specific PTM (for example, CSAP, MACF1*(C1023)*, MAP7D1 and Tau*(0N-3R)*, each showing an about 2-fold reduced binding to detyrosinated HeLa microtubules and an about 2-fold increased binding to polyglutamylated brain microtubules), was not sufficient to predict the overall effect on the binding of these MAPs to native microtubules from different sources: While CSAP bound preferentially to HeLa over brain microtubules, MACF1*(C1023)*, MAP7D1 and Tau*(0N-3R)* showed a strong preference for brain microtubules (Appendix Figs. S6, S11, S19 and S23). Thus, we find that combinatorial effects of concurrent PTMs are not entirely predictable and instead depend on each MAP individually, which underpins the importance of direct tests.

Our results thus provide experimental evidence for the basic assumption of the tubulin code—that combinations of tubulin PTMs create unique ‘codes’ on microtubules, which can be specifically and selectively understood by ‘readers’ (i.e. MAPs). That polyglutamylation and detyrosination/tyrosination dominate in the regulation of microtubule-MAP interactions is not surprising in light of their position on the microtubule lattice: both PTMs take place at the carboxy-terminal tails of tubulin, which protrude from the outer microtubule surface where most MAP interactions take place. Moreover, the use of stabilised microtubules in our assays might preclude the possibility that MAPs can reach into the microtubule lumen where acetylation is taking place, which, however, could happen in dynamic microtubules (Nihongaki et al, 2021). Our observations thus do not exclude the possibility that acetylation or the presence of different tubulin isotypes could also affect MAP–microtubule interactions.

### Changing the readout of the tubulin code by alternative splicing

Several of the here-tested MAPs exist in different splice variants. The well-characterised MAPs Tau, MAP2 and MAP4 are spliced in their microtubule-binding regions (Chapin et al, 1995; Doll et al, 1993; Jakes et al, 1991). In the current work, Tau*(0N-3R)* showed similar binding preferences as Tau*(2N-4R)* in our previous study (Genova et al, 2023), and the two MAP4 isoforms X16 and X20 also showed similar binding preferences to the different PTM variants (Appendix Figs. S15 and S16).

**Figure 4:**
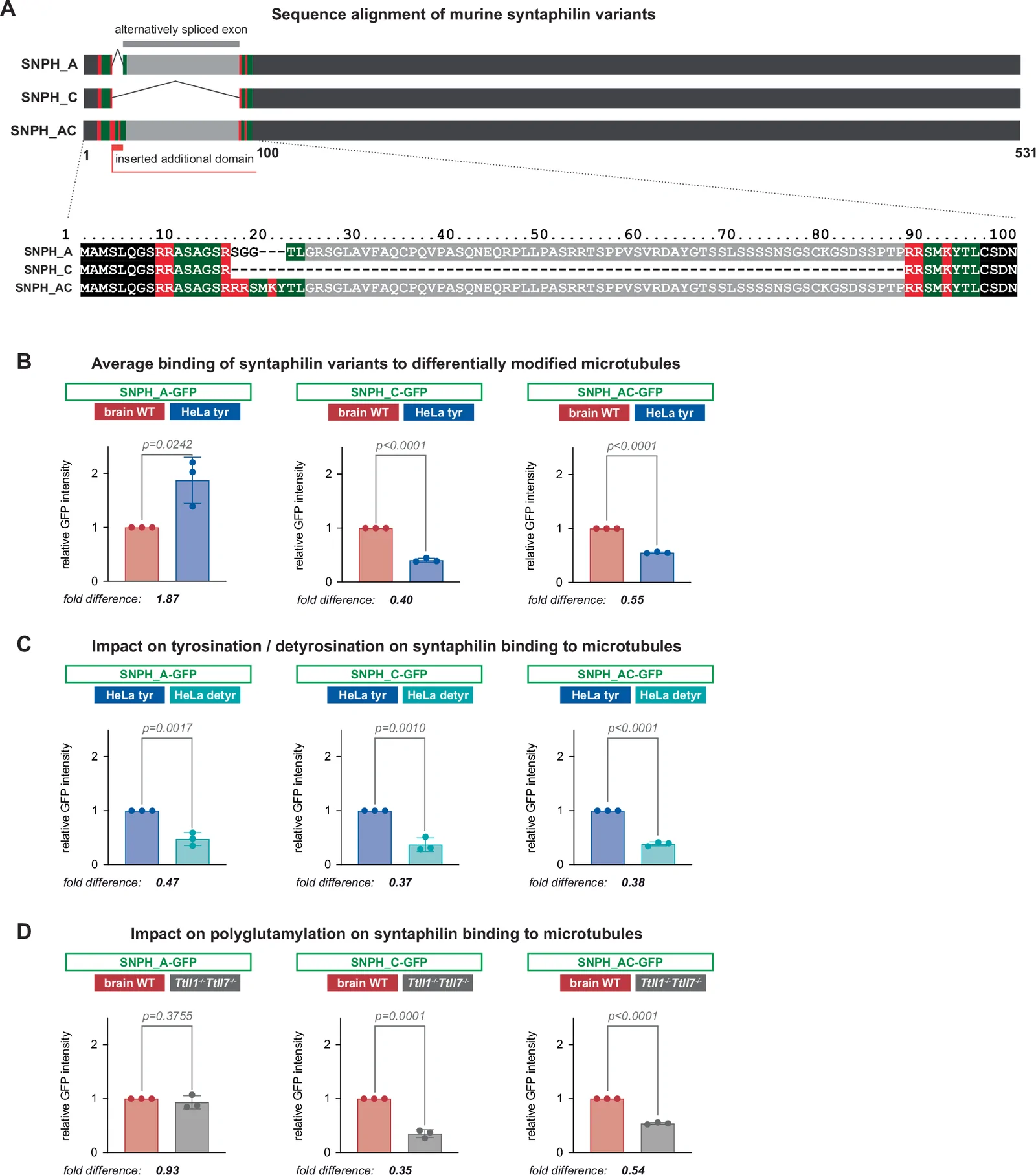
Alternative splicing of Syntaphilin modulates microtubule-binding preferences. (**A**) Schematic representation of splicing isoforms of the mouse Syntaphilin gene: SNPH_A, SNPH_C, and the chimeric construct SNPH_AC chimera. The alternatively spliced exon is labelled in light grey. Positively charged domains are red, other conserved amino acids within this domain are shown in dark green. The domain that is generated by the joining of the two exon boundaries in SNPH_C is re-created without the splicing event in the SNPH_AC chimera. Zoom view shows amino acid sequence alignment. (**B**–**D**) Average of quantifications of microtubule binding of SNPH-GFP variants (plots from Appendix Figs. S21C, S22C and S25C). The complete datasets are shown for SNPH_A (Appendix Fig. S21), SNPH_C (Appendix Fig. S22) and SNPH_AC (Appendix Fig. S25). (**B**) differential binding of SNPH variants between wild-type brain (red) and HeLa (dark blue) microtubules. (**C**) Differential binding of SNPH variants to tyrosinated (dark blue) and detyrosinated (light blue) HeLa microtubules. (**D**) Differential binding of SNPH variants to polyglutamylated (red) and low-glutamylated (grey; *Ttll1*^*−/−*^*Ttll7*^*−/−*^) brain microtubules. Each data point represents the mean value from an independent assay, as shown in Appendix Figs. S21, S22 and S25. Statistical significance was determined using unpaired *t* tests; *P* values are displayed above each comparison, and fold differences are shown below.

Syntaphilin (SNPH), by contrast, is alternatively spliced in a so-far little-characterised domain. The two splice isoforms of syntaphilin we tested here differ in an amino-terminally located stretch of 69 amino acids present in SNPH_A (amino acids 17–85) and spliced out in SNPH_C (Fig. 4A). Comparing the two isoforms, we first observed opposite preferences for HeLa and brain tubulin: SNPH_A bound stronger to HeLa, while SNPH_C preferred brain microtubules (Fig. 4B; Appendix Figs. S21 and S22). When we verified the impact of single tubulin PTMs, we found that both isoforms bound preferentially to tyrosinated over detyrosinated tubulin (Fig. 4C; Appendix Figs. S21 and S22), but showed a striking difference in their sensitivity to polyglutamylation: SNPH_A was insensitive to this PTM, whereas SNPH_C bound much more efficiently to polyglutamylated microtubules (Fig. 4D; Appendix Figs. S21 and S22). It thus appears that with a single splicing event, the preference of SNPH_A for non-modified HeLa microtubules is switched to a preference for highly modified brain microtubules by generating a previously non-existent sensitivity for polyglutamylation in SNPH_C.

But how is this preference for polyglutamylation generated? As the splicing event removes a part of the amino acid sequence, the only perceivable possibility is that a new sequence motif is generated from the two sequences flanking the spliced-out exon. Indeed, the alternatively spliced region is flanked by two positively charged sequence motifs, which after splicing are joined in the SNPH_C isoform, generating a unique sequence motif which cannot be found in any other mammalian protein. To test whether the formation of this positively charged motif is sufficient to generate a sensitivity to polyglutamylation, we created the same sequence motif within the SNPH_A isoform by retaining the alternatively spliced exon (Fig. 4A). Strikingly, the chimeric SNPH_AC protein behaved like SNPH_C in our in vitro binding assays: it preferentially binds to brain over HeLa microtubules, and it shows a preference to polyglutamylated microtubules with a similar fold change as SNPH_C (Fig. 4B,C,D; Appendix Fig. S25). This demonstrates that the inclusion of the alternatively spliced exon, possibly arisen evolutionary in an event of exonisation (Keren et al, 2010; Schmitz and Brosius, 2011), disrupts a functional protein domain able to sense microtubule polyglutamylation. The example of syntaphilin demonstrates that the regulation of splicing events can serve as a switch that changes the behaviour of MAPs in cells, for instance, by guiding them to different types of microtubules that are differentially modified following physiological reprogramming of the cytoskeleton.

### Differential PTM reading within the highly conserved EML protein family

**Figure 5:**
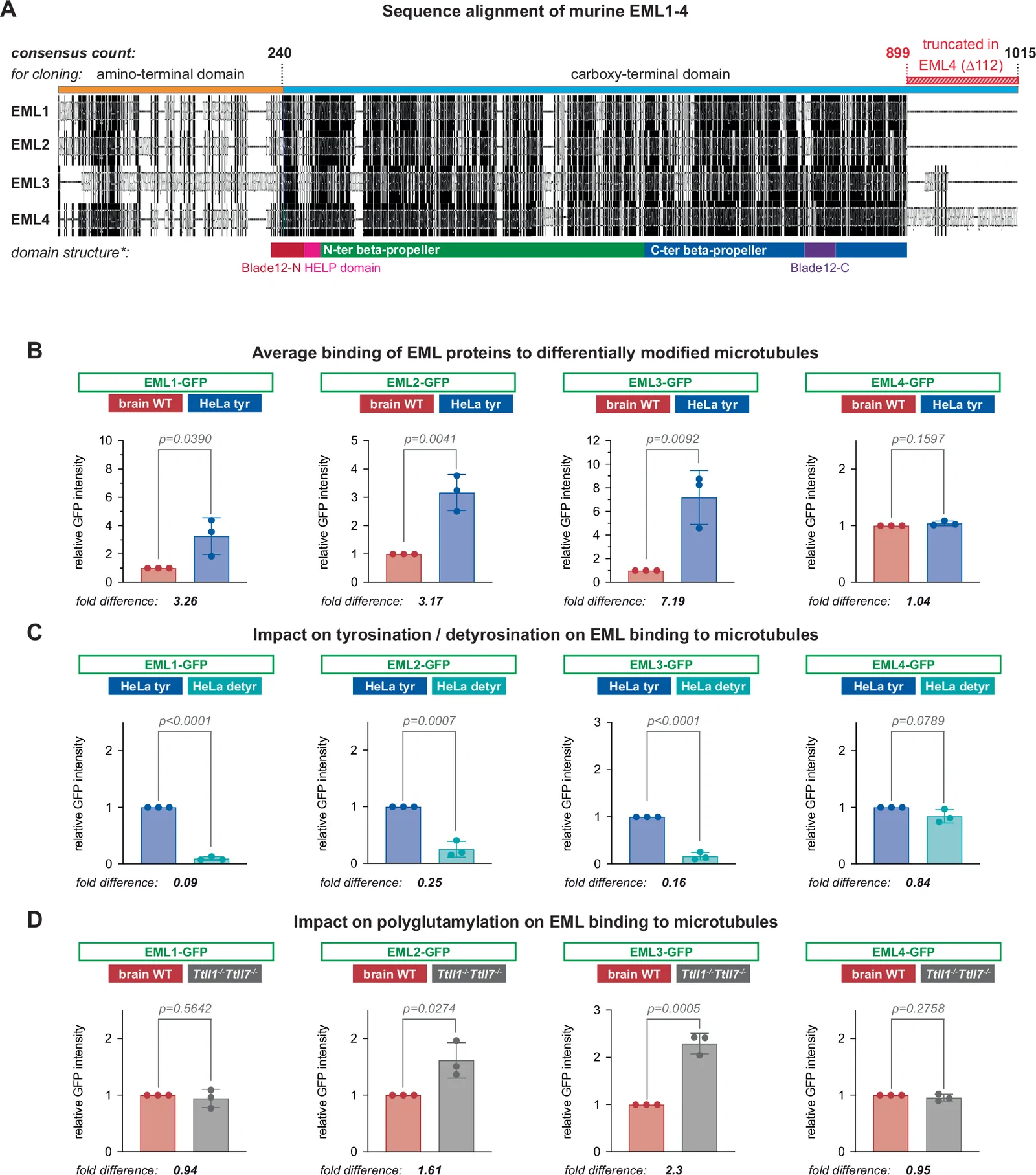
Comparative analysis of EML1-4 microtubule binding. (**A**) Schematic representation of a sequence alignment (Fig. EV1) of mouse EML1-4 proteins, with black-coloured regions indicating conserved residues, and lighter colours representing sequence dissimilarities. Numbers indicate the amino acid count of the consensus. Note that the sequence conservation in the amino-terminal domains (orange) is lower than in the carboxy-terminal domains (blue). The red-hatched box indicates the unique carboxy-terminal sequence of EML4. *A previously determined domain structure of human EML1 (Richards et al, 2014) adapted to the current alignment is shown below the aligned sequences. (**B**–**D**) Average of quantifications of microtubule binding of EML1-4-GFP. The complete datasets are represented for EML1 (plots from Appendix Fig. S7), EML2 (plots from Appendix Fig. S8), EML3 (Appendix Fig. S9), and EML4 (plots from Appendix Fig. S10). (**B**) differential binding of EML proteins between wild-type brain (red) and HeLa (dark blue) microtubules. (**C**) Differential binding of EMLs to tyrosinated (dark blue) and detyrosinated (light blue) HeLa microtubules. (**D**) Differential binding of EMLs to polyglutamylated (red) and low-glutamylated (grey; *Ttll1*^*−/−*^*Ttll7*^*−/−*^) brain microtubules. Each data point represents the mean value from an independent assay shown in Appendix Figs. S7–10. Statistical significance was determined using unpaired *t* tests; *P* values are displayed above each comparison, and fold differences are shown below.

Echinoderm microtubule-associated proteins (EMAPs) are a family of MAPs of which the first member was identified in sea urchin eggs as a protein associated with the mitotic spindle (Suprenant et al, 1993; Suprenant and Marsh, 1987). EMAP has six homologues in mammals, EMAP-like (EML)1-6, out of which four, EML1-4, are highly similar in terms of domain organisation and primary peptide sequences (Fig. 5A). EML1-4 bind microtubules in cells and in in vitro reconstitutions (Eichenmüller et al, 2002; Hotta et al, 2022; Jijumon et al, 2022; Pollmann et al, 2006; Richards et al, 2015).

**Figure EV1:**
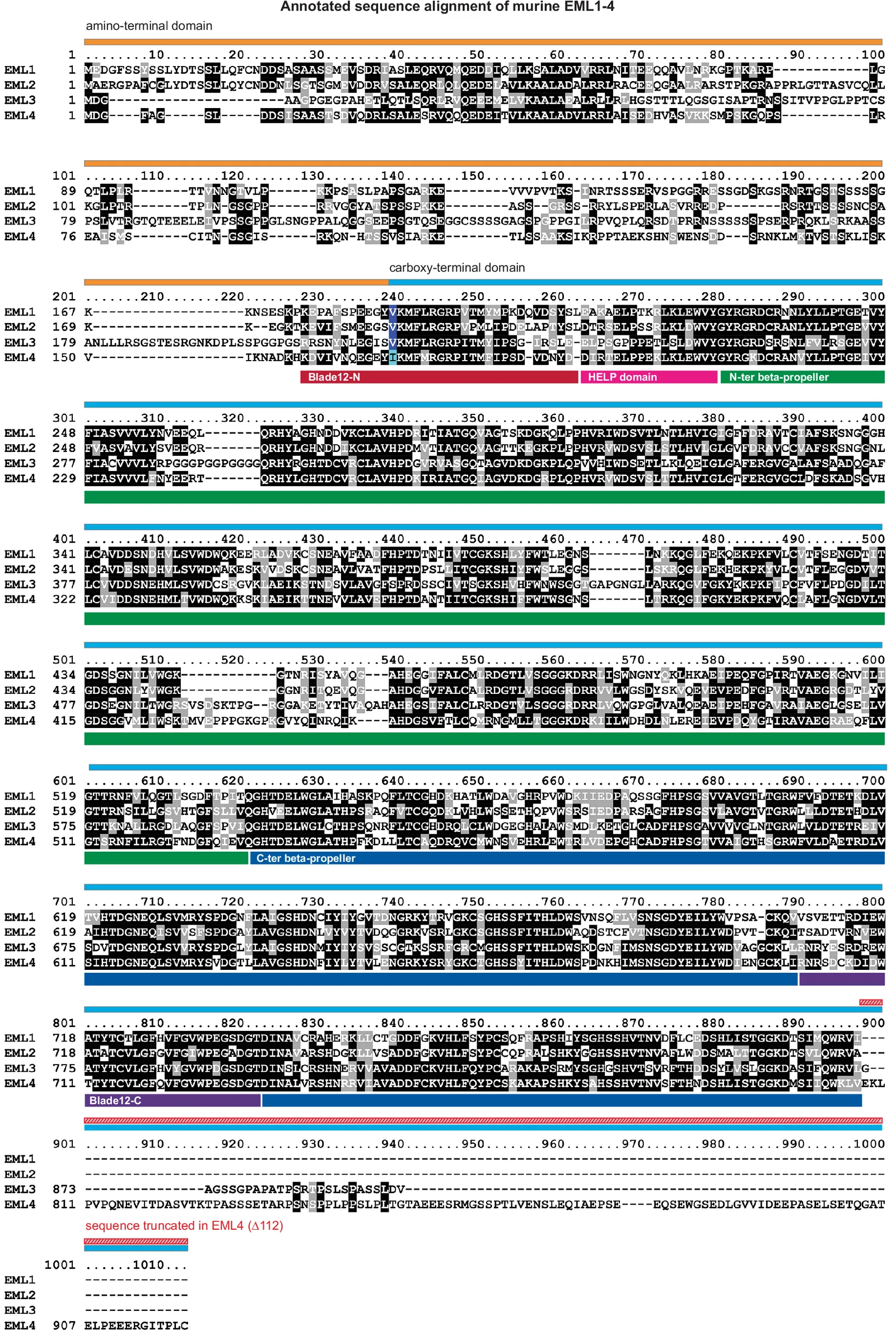
Amino acid sequence alignment of murine EML1-4 proteins. Amino acid sequences of murine EML1, EML2, EML3, and EML4 proteins were aligned to identify conserved regions. Dark-coloured regions indicate conserved residues shared among the EML family members. Amino acid numbers refer to the consensus of the alignment. Above the aligned sequences: The orange-coloured box marks the amino-terminal (N-terminal) domains, while the blue-boxed region represents the carboxy-terminal (C-terminal) domains we used for cloning truncated and chimeric constructs used in Figs. EV3–EV6. The red-hatched box indicates the C-terminal domain unique to EML4 that was deleted in the construct used in Fig. EV6. *A previously determined domain structure of human EML1 (Richards et al, 2014) adapted to the current alignment is shown below the aligned sequences.

**Figure EV2:**
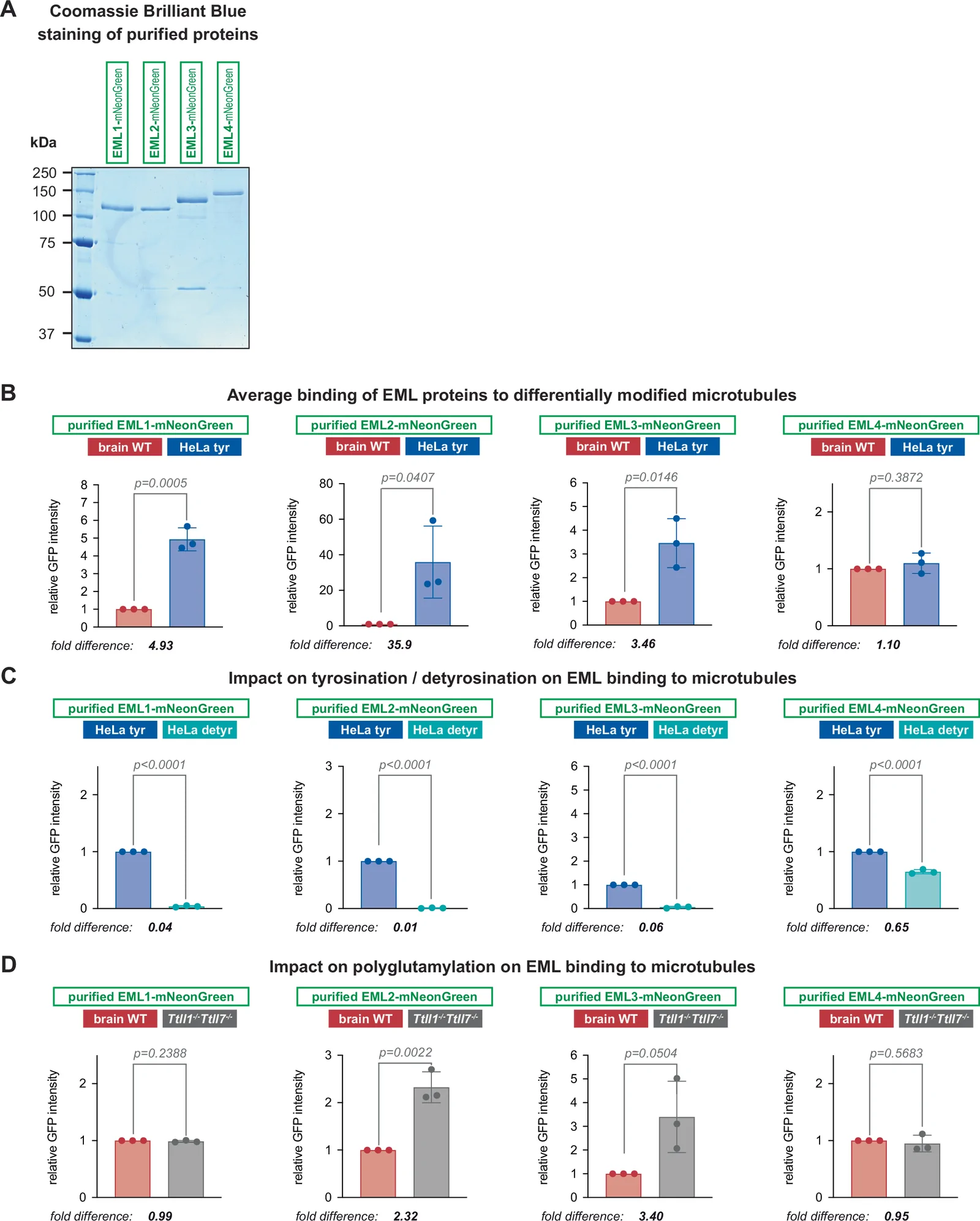
Comparative analysis of EML1-4 microtubule binding using purified proteins. (**A**) Purified EML-mNeonGreen proteins were run on SDS-PAGE gels and stained with Coomassie Brilliant Blue. (**B**–**D**) Average of quantifications of microtubule binding of purified EML1-4-mNeonGreen. The complete datasets are represented for EML1 (plots from Appendix Fig. S26), EML2 (plots from Appendix Fig. S27), EML3 (plots from Appendix Fig. S28) and EML4 (plots from Appendix Fig. S29). (**B**) differential binding of EML proteins between wild-type brain (red) and HeLa (dark blue) microtubules. (**C**) Differential binding of EMLs to tyrosinated (dark blue) and detyrosinated (light blue) HeLa microtubules. (**D**) Differential binding of EMLs to polyglutamylated (red) and low-glutamylated (grey; *Ttll1*^*−/−*^*Ttll7*^*−/−*^) brain microtubules. Each data point represents the mean value from an independent assay shown in Appendix Figs. S26–29. Statistical significance was determined using unpaired *t* tests; *P* values are displayed above each comparison, and fold differences are shown below.

Despite their high level of sequence conservation and similar domain structures (Figs. 5A and EV1), EML1-4 showed different binding preferences in our initial screen: EML1, EML2, and EML3, but not EML4 preferentially bound to HeLa over brain microtubules (Fig. 5B). In the subsequent analyses, we found even more differences: EML1 was sensitive to tyrosination level, but not to any other PTM (Fig. 5C,D; Appendix Fig. S7), while EML2 (Fig. 5C,D; Appendix Fig. S8) and EML3 (Fig. 5C,D; Appendix Fig. S9) preferentially bound to tyrosinated as well as non-glutamylated microtubules. EML4, by contrast, did not show preferential binding to any PTM or isotype variants tested here (Fig. 5C,D; Appendix Fig. S10). To confirm that those binding preferences are intrinsic features of the EML proteins and not related to other proteins present in the cell lysates, we repeated all experiments with proteins purified from insect cells, which confirmed the initial results (Fig. EV2; Appendix Figs. S26–S29).

**Figure EV3:**
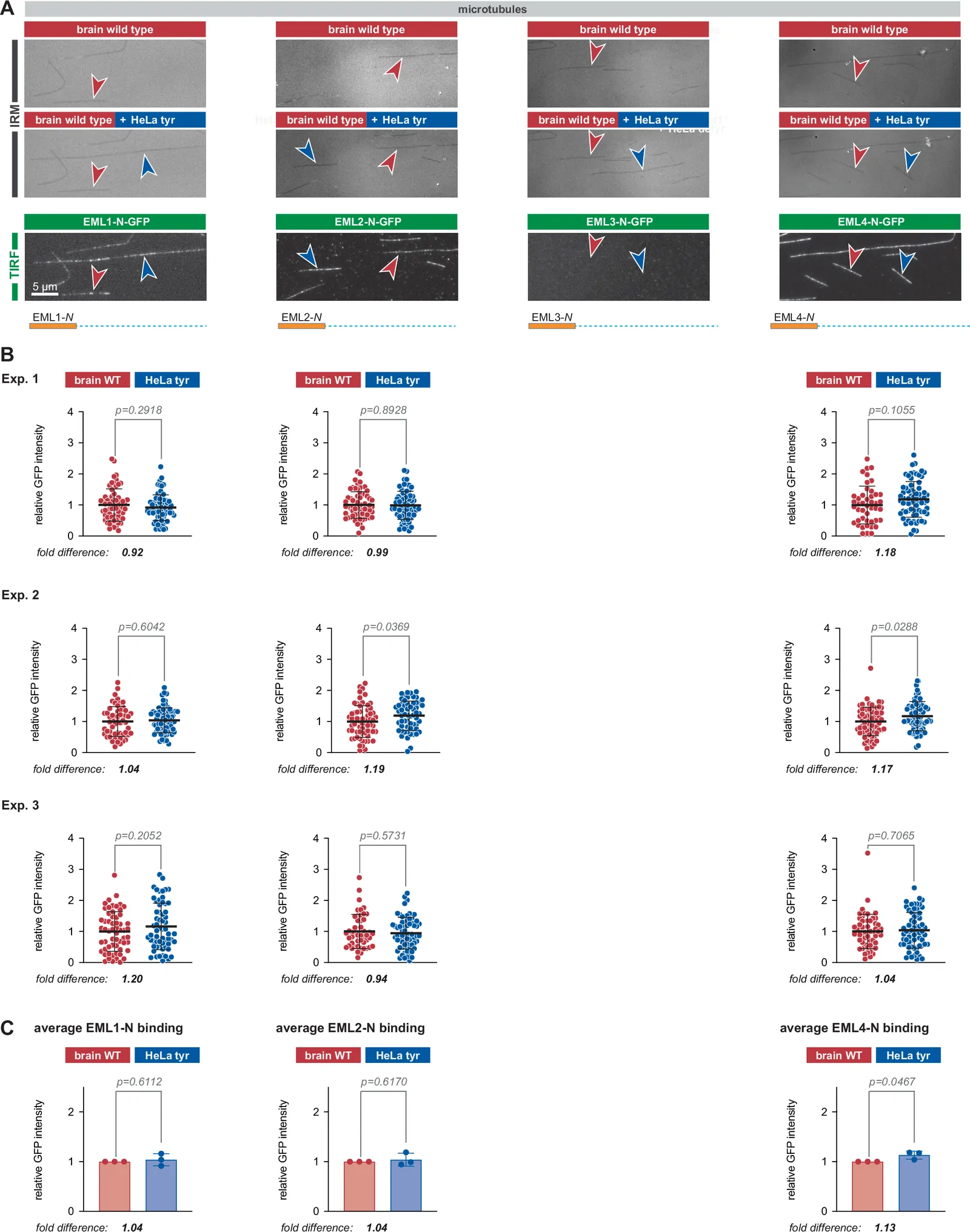
Binding of truncated EML1-4 amino termini to brain and HeLa microtubules. (**A**) TIRF microscopy-based assay showing the amino-terminal domains of EML1-4 (EML1-*N*, 2-*N*, 3-*N*, 4-*N*; Fig. 5A) from cell lysates to Taxol-stabilised microtubules polymerised from either wild-type brain or wild-type HeLa tubulin. IRM images depict microtubules formed from brain and HeLa tubulin added sequentially, with corresponding snapshots and TIRF images displaying MAP binding. (**B**) Quantification of binding intensity from three independent assays. Each dot represents a single microtubule. EML3-N binding was too weak to be clearly visualised and therefore could not be quantified. (**C**) Average binding of the amino-terminal domains of EML1-4 to brain versus HeLa microtubules. Each data point represents the mean value from one assay. (**B**, **C**) Red denotes wild-type brain microtubules; dark blue denotes wild-type HeLa microtubules. Statistical significance was assessed using unpaired *t* tests; *P* values are indicated above each comparison, and fold changes are shown below. All experiments were performed in triplicate using independently purified tubulin and EML-*N* lysates prepared from three separate cell batches. Source data are available online for this figure.

PTM specificity might be directly encoded in the microtubule-binding domains. Previous work had defined the amino-terminal part of EMLs as microtubule-binding domains (Adib et al, 2019; Hotta et al, 2022; Richards et al, 2015; Figs. 5A and EV1). Indeed, versions of EMLs lacking these domains did not bind microtubules in our experiments. We thus generated GFP-tagged constructs of the amino-terminal presumed microtubule-binding and trimerisation domains of EML1-4 following the domain assignment from previous work (Adib et al, 2019) and assessed their binding to HeLa and brain microtubules using cell lysates. Unexpectedly, these domains alone did not show preferences for the two microtubule variants, with the EML3 construct not showing any detectable microtubule binding (Fig. EV3). It thus appears that the amino-terminal domains alone are not responsible for the microtubule binding of EMLs; instead, they might be necessary but not sufficient parts of larger microtubule-binding modules. This would predict that the PTM preferences of EMLs are encoded by an entire module, composed of sequences localised in both, amino- and carboxy-terminal domains.

**Figure EV4:**
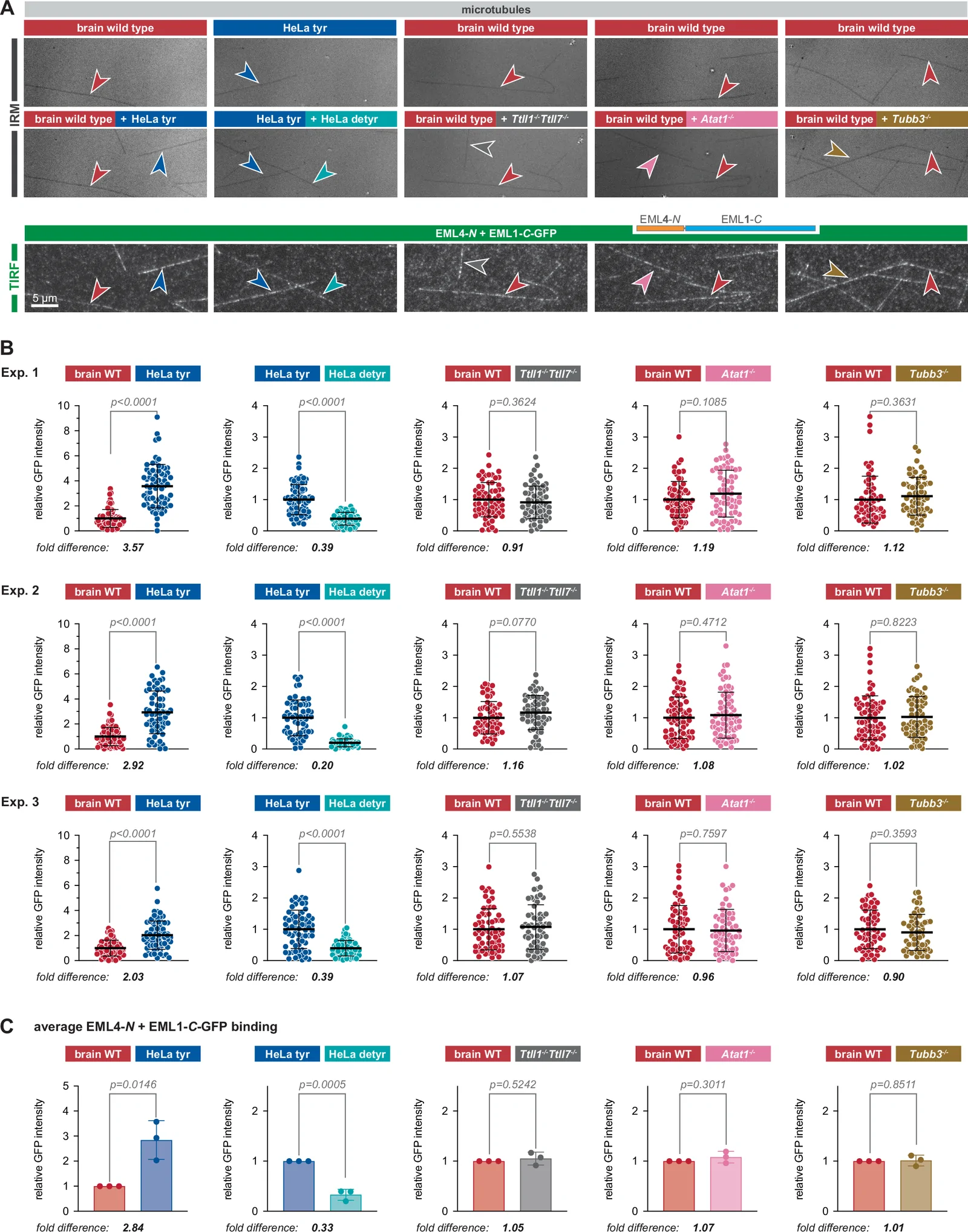
Binding of a chimaera of amino- and carboxy-terminal domains of EML4 and EML1 to microtubules with distinct tubulin PTMs and isotype compositions. (**A**) TIRF microscopy-based assay showing the binding of a chimeric construct comprising the amino-terminal sequence of EML4 (EML4-*N*) fused to the carboxy-terminal sequence of EML1 (EML1-*C*; Fig. 5A) from cell lysates to Taxol-stabilised microtubules polymerised from tubulin sources with different PTMs and isotype compositions. IRM images show microtubules added sequentially, with corresponding snapshots and TIRF images displaying binding of the chimeric construct. (**B**) Quantification of EML4-*N* + EML1-*C*-GFP chimaera intensities in three independent assays. Fluorescence values are normalised to wild-type microtubules (either wild-type brain or wild-type HeLa). Each data point represents a single microtubule. Bars represent mean ± standard deviation. (**C**) Average binding of the chimeric EML4-*N* + EML1-*C*-GFP construct to different microtubule types. Each data point represents the mean value from one independent assay. (**B**, **C**) Red indicates wild-type brain microtubules; dark blue, wild-type HeLa; light blue, detyrosinated HeLa; grey, *Ttll1*^*−/−*^*Ttll7*^*−/−*^; pink, *Atat1*^*−/−*^; and golden brown, *Tubb3*^*−/−*^ mouse brain-derived microtubules. Statistical significance was determined using unpaired *t* tests; *P* values are shown above each comparison, and fold differences are indicated below. All experiments were performed in triplicate using independently purified tubulin and chimeric construct lysates prepared from three separate cell batches. Source data are available online for this figure.

**Figure EV5:**
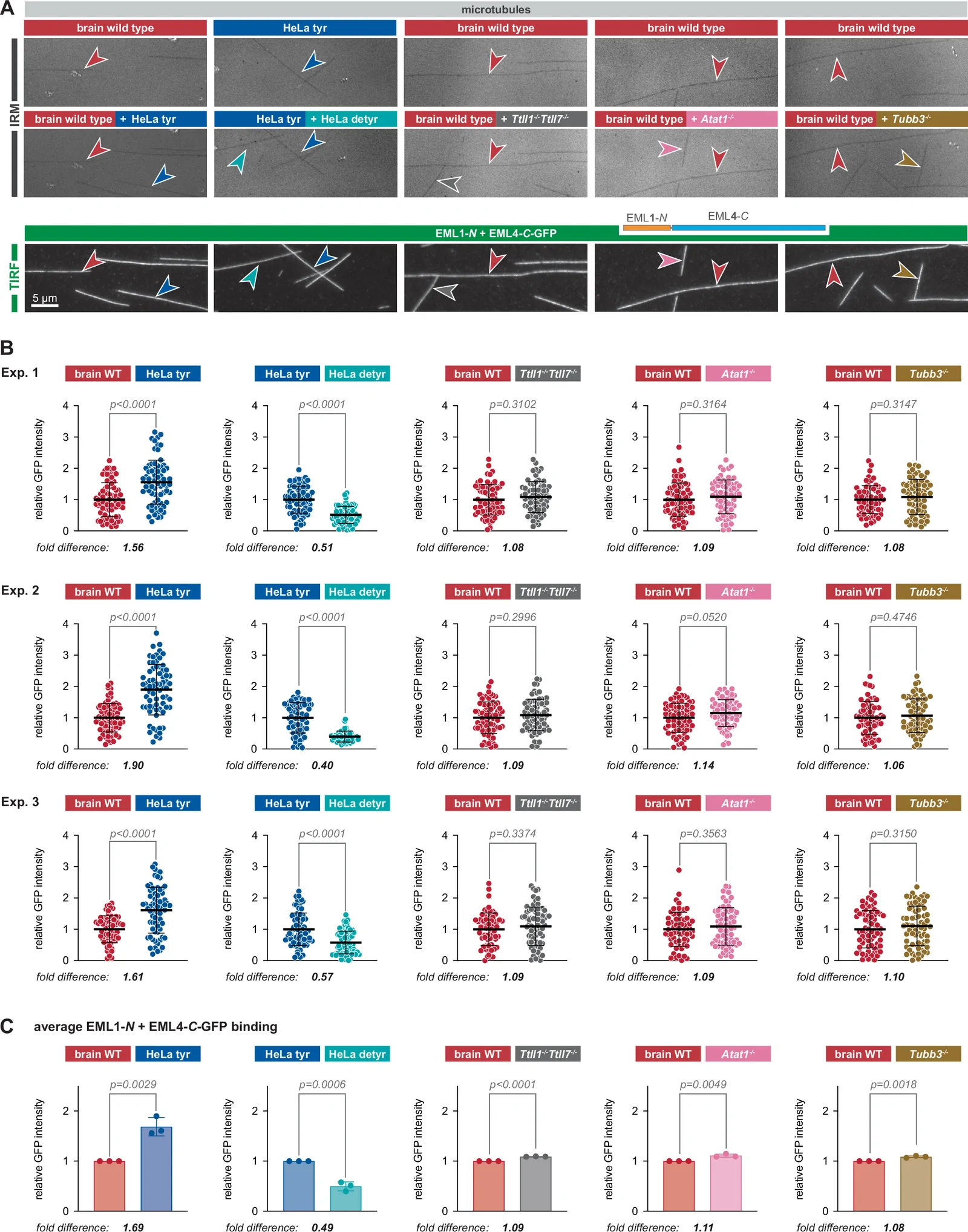
Binding of a chimaera of amino- and carboxy-terminal domains of EML1 and EML4 to microtubules with distinct tubulin PTMs and isotype compositions. (**A**) TIRF microscopy-based assay showing the binding of a chimeric construct comprising the amino-terminal sequence of EML1 (EML1-*N*) fused to the carboxy-terminal sequence of EML4 (EML4-*C*; Fig. 5A) from cell lysates to Taxol-stabilised microtubules polymerised from tubulin sources with different PTMs and isotype compositions. IRM images show microtubules added sequentially, with corresponding snapshots and TIRF images displaying binding of the chimeric construct. (**B**) Quantification of EML1-*N* + EML4-*C*-GFP chimaera intensities in three independent assays. Fluorescence values are normalised to wild-type microtubules (either wild-type brain or wild-type HeLa). Each data point represents a single microtubule. Bars represent mean ± standard deviation. (**C**) Average binding of the chimeric EML1-*N* + EML4-*C*-GFP to different microtubule types. Each data point represents the mean value from one independent assay. (**B**, **C**) Red indicates wild-type brain microtubules; dark blue, wild-type HeLa; light blue, detyrosinated HeLa; grey, *Ttll1*^*−/−*^*Ttll7*^*−/−*^; pink, *Atat1*^*−/−*^ and golden brown, *Tubb3*^*−/−*^ mouse brain-derived microtubules. Statistical significance was determined using unpaired *t* tests; *P* values are shown above each comparison, and fold differences are indicated below. All experiments were performed in triplicate using independently purified tubulin and chimeric construct lysates prepared from three separate cell batches. Source data are available online for this figure.

**Figure EV6:**
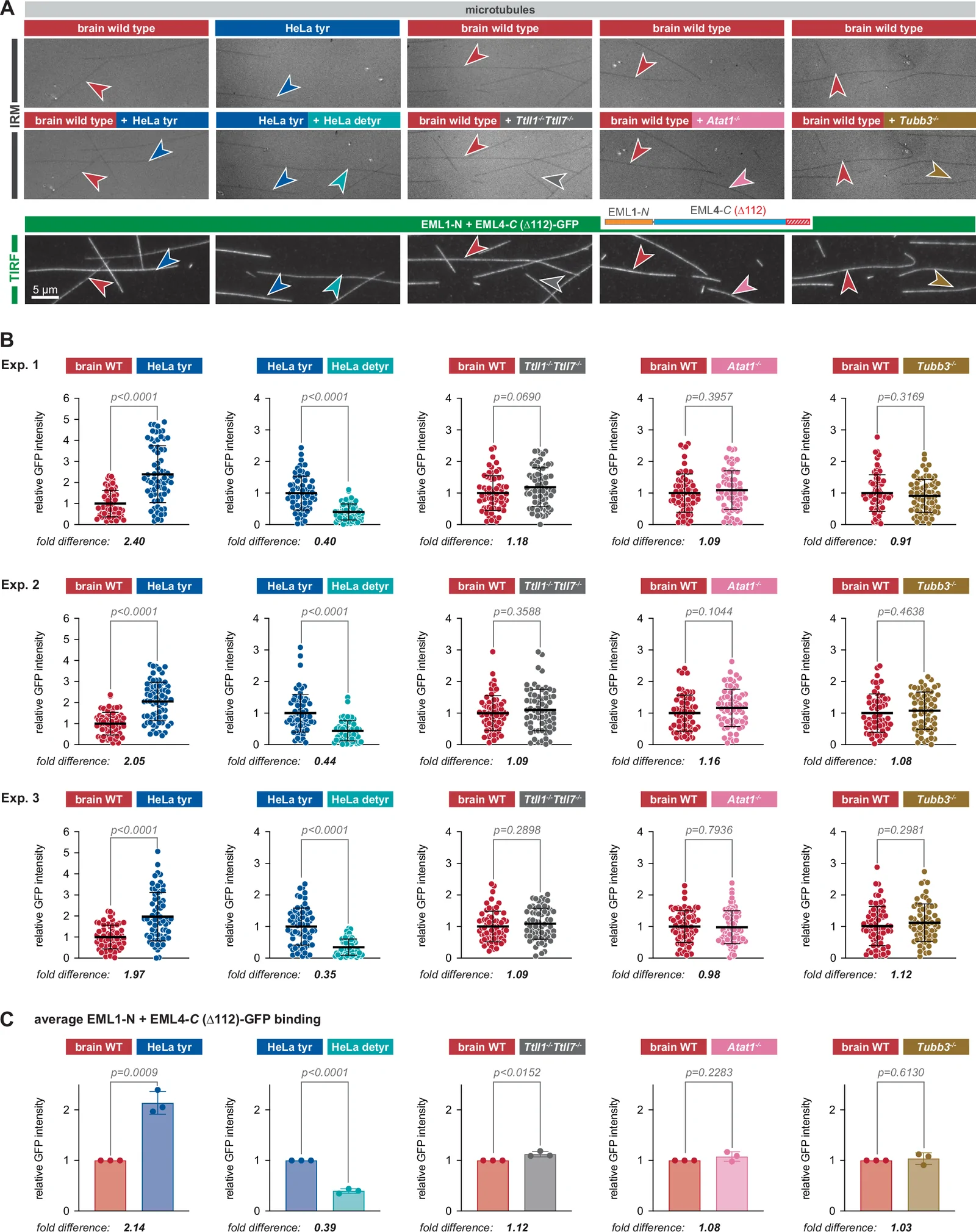
Binding of a chimaera of the amino-terminal domains of EML1 and the truncated carboxy-terminal domain of EML4 to microtubules with distinct tubulin PTMs and isotype compositions. (**A**) TIRF microscopy-based assay showing the binding of a chimeric construct comprising the amino-terminal sequence of EML1 (EML1-*N*) fused to the carboxy-terminal sequence of EML4 lacking the unique 112 carboxy-terminal amino-acid stretch that is absent in EML1 (EML4-*C*(∆112); Fig. 5A) from cell lysates to Taxol-stabilised microtubules polymerised from tubulin sources with different PTMs and isotype compositions. IRM images show microtubules added sequentially, with corresponding snapshots and TIRF images displaying binding of the chimeric construct. (**B**) Quantification of EML1-*N* + EML4-*C*(∆112)-GFP chimaera intensities in three independent assays. Fluorescence values are normalised to wild-type microtubules (either wild-type brain or wild-type HeLa). Each data point represents a single microtubule. Bars represent mean ± standard deviation. (**C**) Average binding of the chimeric EML1-*N* + EML4-*C*(∆112)-GFP construct to different microtubule types. Each data point represents the mean value from one independent assay. (**B**, **C**) Red indicates wild-type brain microtubules; dark blue, wild-type HeLa; light blue, detyrosinated HeLa; grey, *Ttll1*^*−/−*^*Ttll7*^*−/−*^; pink, *Atat1*^*−/−*^ and golden brown, *Tubb3*^*−/−*^ mouse brain-derived microtubules. Statistical significance was determined using unpaired *t* tests; *P* values are shown above each comparison, and fold differences are indicated below. All experiments were performed in triplicate using independently purified tubulin and chimeric construct lysates prepared from three separate cell batches. Source data are available online for this figure.

To test this hypothesis, we generated chimeric proteins swapping the amino- and carboxy-terminal domains of EML1 and EML4 (Figs. 5A, EV4 and EV5). Both chimeric proteins bound to microtubules, indicating that the amino- and carboxy-termini of EMLs are functionally redundant and exchangeable. Strikingly, both chimaeras showed a preference for tyrosinated microtubules; their binding was reduced by about 50% to detyrosinated as compared to tyrosinated microtubules (Figs. EV4 and EV5). This, however, was less pronounced to what was observed for wild-type EML1, which almost entirely loses its capacity to bind microtubules if they are detyrosinated (Fig. 5C; Appendix Fig. S7). Deletion of a 112-amino-acid sequence that is uniquely found at the carboxy-terminus of EML4, but not present on EML1, did not change this result (Fig. EV6). Together, these results suggest that both the amino- and carboxy-terminal domains participate not only in microtubule binding of EML proteins, but also in their reading of the microtubule PTM status, while the unique carboxy-terminal domain found on EML4 has little impact on PTM preferences. Our results reveal that more complex, multi-domain recognition motives than the previously identified CAP-Gly domain (Bieling et al, 2008; Peris et al, 2006) can be involved in the recognition of tubulin tyrosination.

### Control of EML–microtubule interactions by tyrosination in cells

**Figure EV7:**
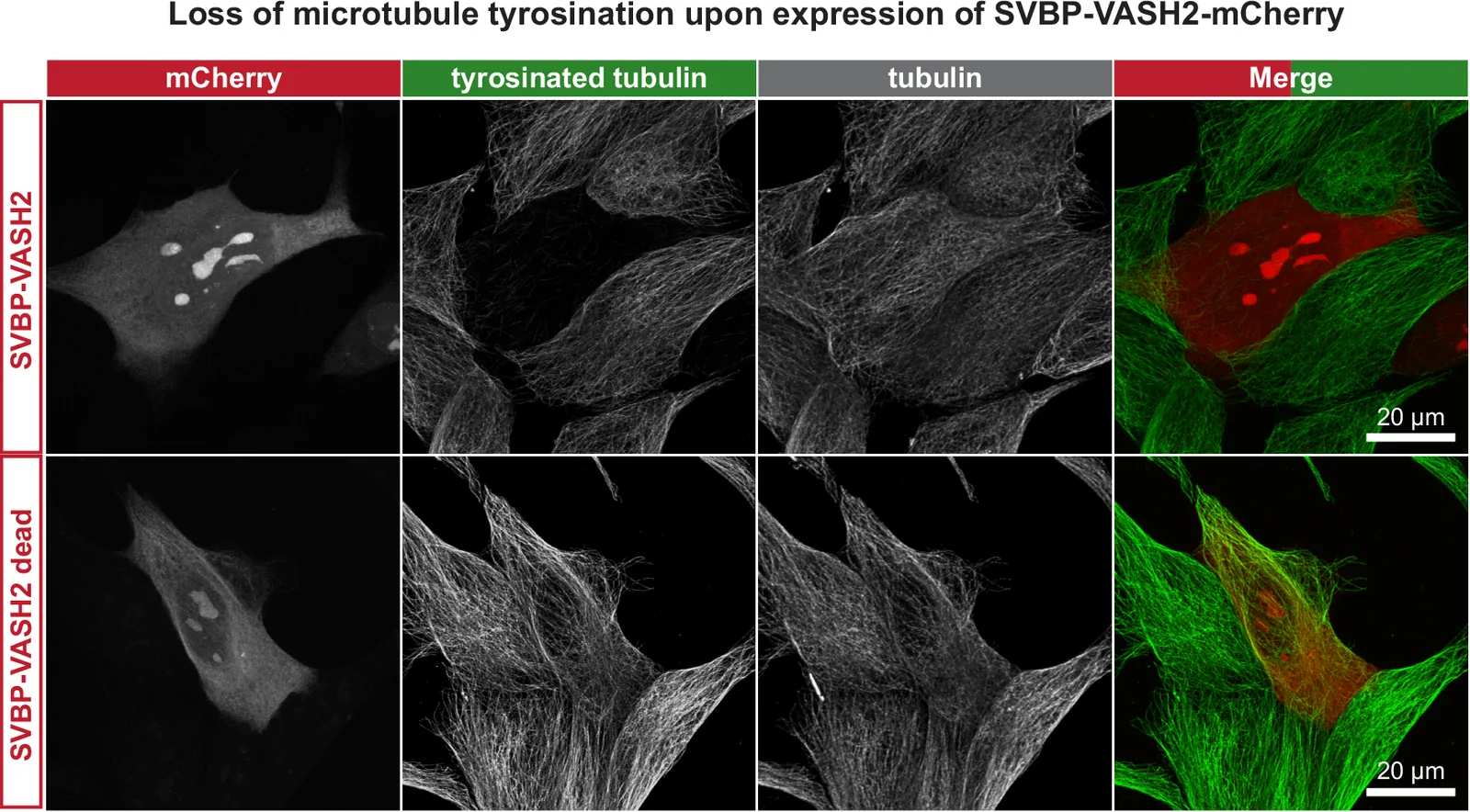
Loss of tyrosination in cells expressing Vash2-Svbp. Immunofluorescence staining of MDCK cells transiently transfected with either mCherry-tagged active Vash2-Svbp or with the catalytically inactive mutant of this enzyme. Cells were fixed and stained with anti-tyrosinated tubulin antibody (YL1/2). Note that cells expressing active, but not inactive Vash2-Svbp-mCherry, entirely lose YL1/2 labelling of microtubules, indicating full detyrosination. Scale bars: 20 µm.

To confirm that the binding preferences we determined in our in vitro settings are relevant for MAP–microtubule interactions in living cells, we chose to investigate EML proteins because of the differential and strong impact of detyrosination on the binding of some but not all EMLs to microtubules (Fig. 5; Appendix Figs. S7–10). To abolish tyrosination, which is ubiquitously present on microtubules in the MDCK cell line, we overexpressed the tubulin detyrosinating enzyme Svbp-Vash2 (Aillaud et al, 2017; Nieuwenhuis et al, 2017), which, as previously shown (Kesarwani et al, 2020), is an effective tool to remove tyrosination from all microtubules in cells. Indeed, overexpression of Svbp-Vash2, but not of the enzymatically dead mutant, entirely detyrosinated all microtubules (Fig. EV7).

**Figure 6:**
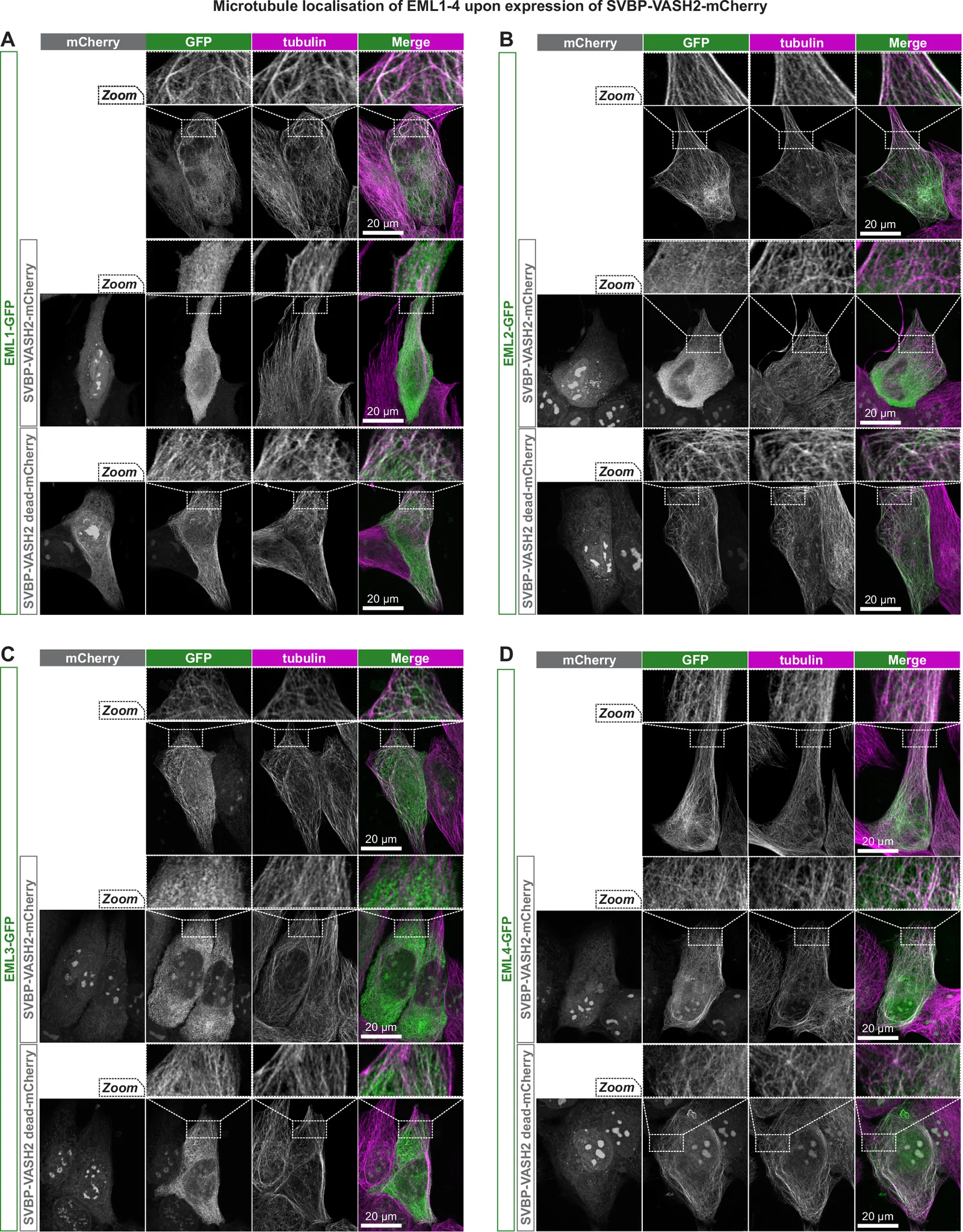
Impact of tyrosination on binding of EML proteins to microtubules in cells. (**A**–**D**) Immunofluorescence staining of MDCK cells transiently transfected with GFP-tagged EML1 (**A**), EML2 (**B**), EML3 (**C**), or EML4 (**D**), co-expressed with either mCherry-tagged active Vash2-Svbp enzyme (causing complete loss of microtubule tyrosination; Fig. EV7) or its catalytically inactive mutant as a control. Cells were fixed and stained with anti-tubulin antibody to visualise microtubules. GFP fluorescence indicates the localisation of EML-GFP proteins, and mCherry fluorescence marks the expression of Vash2-Svbp. Note the differences in EML-GFP binding to microtubules upon expression of active, but not inactive, Vash2-Svbp-mCherry. Zoom sections highlight parts of the cells where microtubules are particularly well visible. Scale bars: 20 µm. Source data are available online for this figure.

Next, we expressed EML(1-4)-GFP alone, co-expressed with Svbp-Vash2-mCherry, or with the enzymatically dead mutant of this enzyme as a control. While in the control condition and in the presence of an enzymatically dead enzyme, where microtubules are tyrosinated (Fig. EV7), all four EMLs showed strong co-localisation with microtubules. EML1 is entirely delocalised from microtubules when co-expressed with Svbp-Vash2-mCherry (Fig. 6A), and EML2 as well as EML3 binding is strongly reduced (Fig. 6B,C). By contrast, binding of EML4 is not affected by the expression of Svbp-Vash2-mCherry, confirming the insensitivity of this protein to detyrosination that was found in vitro (Fig. 5B,C) in cells (Fig. 6D). These experiments demonstrate that even under the condition of overexpression, when MAPs are present at saturating concentrations and tend to strongly bind and bundle microtubules (Jijumon et al, 2022), changes in tubulin PTM levels have the capacity to completely impede the binding of certain MAPs to microtubules in living cells (Fig. 6). The experiments in cells validate our in vitro observations and confirm the presence of selective regulatory mechanisms of EML proteins in response to different tubulin PTMs.

## Discussion

The key question we addressed in our study is whether the signals generated by the tubulin code can be specifically and, most importantly, differentially ‘read’ by a wide variety of microtubule interactors. To answer this question, we screened binding preferences of a comprehensive selection of 46 mammalian proteins that are known as, or were suggested to be, MAPs. A key requirement for our work was the possibility to screen many proteins under similar conditions. Purification of proteins is a limiting step for many in vitro reconstitution experiments and would have strongly limited the number of MAPs to be screened due to time limitations and technical challenges. We thus chose a cell lysate-based approach adapted from our previous work (Jijumon et al, 2022). Importantly, in contrast to our previous and other studies (Alfaro-Aco and Petry, 2015; Field et al, 2017; Schimert et al, 2019; Soppina et al, 2014), we highly diluted the cell lysates in buffer (Fig. 2A,B), which minimises the effect of cytosolic factors on the interactions we studied. This observation validates the use of cell lysates as a robust and feasible approach for medium-throughput in vitro experiments. However, even diluted, a potential difference in using cell lysates versus purified proteins is that residual cytosolic components could affect MAP–microtubule interactions. Here we tested this possibility by repeating an entire set of experiments with four purified proteins: EML1-4, which demonstrated that the PTM preferences initially identified in cell lysates (Fig. 5) were fully reproduced with the purified proteins (Fig. EV2). Similarly, we reproduced data obtained with purified Tau previously (Genova et al, 2023) in cell lysates. Nonetheless, we cannot exclude that in some cases, cytosolic proteins or other components in the cell lysates may affect the binding of the overexpressed proteins.

By covering an unprecedentedly wide range of MAPs, our study provides direct experimental evidence for the tubulin code hypothesis. Our observations that only about half of the MAPs could sense tubulin PTMs, and that among those that could sense them, each MAP recognised different, and often unique combinations of PTMs, demonstrate that the tubulin code can differentially and selectively regulate microtubule interactions. We further show that PTM-guided MAP re-distribution functions in cells, underpinning how the tubulin code could control the functions of different microtubules side-by-side in cells, where normally not all microtubules are homogeneously modified.

An unexpected finding was that alternative splicing can switch the binding preference of a MAP. On the example of syntaphilin, we demonstrate how splicing out a single exon leads to the emergence of tubulin-PTM sensor domains that can drastically change how a protein reads the tubulin code. The preference for polyglutamylation that emerges after splicing out the exon was strong enough to overwrite the preference of this protein for tyrosinated tubulin, switching its binding preference for HeLa tubulin to brain tubulin in our experiments. It remains an intriguing question whether this splicing event originated in an evolutionary introduction of a new exon (Keren et al, 2010; Schmitz and Brosius, 2011) that led to a loss of sensitivity for polyglutamylation, or whether it represents a generic splicing event that allows the protein to adapt to changing PTM landscapes in cells. Our findings might have far-reaching implications for the function of syntaphilin as a mitochondria adaptor protein that controls the motility of these organelles (Canty et al, 2023; Chen and Sheng, 2013; Kang et al, 2008). Given the importance of mitochondria transport and re-distribution in neurons (Jenkins et al, 2024), where polyglutamylation is a prominent PTM, differential splicing of syntaphilin might play a key role in linking mitochondria homeostasis to tubulin code signalling. Indeed, it has been shown that changes in tubulin polyglutamylation levels strongly affect mitochondria motility in cultured neurons (Bodakuntla et al, 2021; Gilmore-Hall et al, 2019; Magiera et al, 2018).

Similarly unexpected was the discovery that EML proteins, despite their high degree of sequence conservation, show profound differences in their sensitivities to tubulin PTMs. These specificities could be a mechanism assuring that individual EMLs, while all being present at the same time in the same cytosol, are guided to distinct microtubule populations in cells. In the light of the emerging roles of EML proteins in cell division (Adib et al, 2019; Bizzotto et al, 2017; Chen et al, 2015; Luo et al, 2019), and of tubulin PTMs at mitotic spindles (Akera et al, 2017; Barisic et al, 2015; Zadra et al, 2022), it is tempting to hypothesise that tubulin PTMs regulate the distribution of EML proteins among distinct microtubule subpopulations during mitosis. Our observations do not entirely align with previous work that did not find differences in PTM sensitivity for EML1, 2, 3 and 4, while only a shorter isoform of EML2 preferentially interacted with tyrosinated over detyrosinated microtubules (Hotta et al, 2022). We believe that methodological differences, in particular the use of co-pelleting assays that quantify bulk binding of MAPs to microtubules, could mask PTM preferences, e.g. by strongly increasing the cooperativity of MAP–microtubule interactions in the bundles formed in these assays. This finding underpins that multiple and complementary experimental approaches are needed to draw a complete picture of a complex biological process. Given that many PTM-mediated effects we have observed in the current study had rather gradual effects on MAP–microtubule interactions, we expect that more sensitive experimental approaches will find novel tubulin code readers in the future.

While further studies are required to determine the physiological readouts of this regulation, current work at the organism level already highlights important roles in homeostasis and disease. For example, mutations in tubulin-modifying enzymes lead to a broad spectrum of pathologies, such as neurodegeneration (Magiera et al, 2018; Shashi et al, 2018), male fertility defects (Gadadhar et al, 2021; Lin et al, 2024; Vogel et al, 2010), retina degeneration (Astuti et al, 2016; Mercey et al, 2024), or heart disease (Chen et al, 2018; Le Dour et al, 2022; Robison et al, 2016). Understanding pathways that are controlled by a selective regulation of microtubule interactors via tubulin PTMs could thus provide a fresh view on disease mechanisms and might pinpoint novel molecular targets for the development of therapeutic approaches.

## Methods

**Table:** Reagents and tools table

Reagent/resource	Reference or source	Identifier or catalogue number
**Experimental models**		
Ttll1^−/−^/Ttll7^−/−^ mice	Genova et al, 2023	
Atat1^−/−^ mice	Kalebic et al, 2013	
Tubb3^−/−^ mice	Latremoliere et al, 2018	
HEK-293 cells	ATCC	CRL-1573
MDCK cells	ATCC	CRL-2935
HeLa S3 cells	ATCC	CCL-2.2
SF9-ESF insect cells	Expression Systems	94-001 F
**Recombinant DNA**		
Mammalian expression plasmids for fluorescently tagged MAPs	Refer to Appendix Table S1	
pOCC-mEMAP1_mNeonGreen-3C-HIS6	This study	
pOCC-mEMAP2_mNeonGreen-3C-HIS6	This study	
pOCC-mEMAP3_mNeonGreen-3C-HIS6	This study	
pOCC-mEMAP4_mNeonGreen-3C-HIS6	This study	
pTRIP-CAG-mSvbp-mVash2_X1-mCherry-StrepII	This study	
mSvbp-mVash2_X1_mut1-mCherry	This study	
**Antibodies**		
Primary antibodies for immunoblotting	Refer to Appendix Table S3	
Primary antibodies for immunostaining	Refer to Appendix Table S4	
HRP-conjugated secondary antibodies	Refer to Appendix Table S3	
Fluorescent secondary antibodies	Refer to Appendix Table S4	
**Oligonucleotides and other sequence-based reagents**		
Mouse genotyping primers	Refer to Appendix Table S2	
GFP-MAP cloning primers for cell lysate	Refer to Appendix Table S1	
**Chemicals, enzymes and other reagents**		
DMEM	Thermo Fisher Scientific	41965062
Foetal bovine serum (FBS)	Sigma-Aldrich	F7524
L-glutamine	Thermo Fisher Scientific	25030123
Penicillin–streptomycin	Thermo Fisher Scientific	15140130
Trypsin-EDTA	Thermo Fisher Scientific	25200056
jetPEI	Polyplus	101000020
PIPES	Sigma-Aldrich	P6757
EGTA	Sigma-Aldrich	E3889
MgCl_2_	Sigma-Aldrich	M1028
PMSF	Sigma-Aldrich	P7626
Aprotinin	Sigma-Aldrich	A1153
Leupeptin	Sigma-Aldrich	L2884
4-(2-aminoethyl)-benzene sulfonyl fluoride	Sigma-Aldrich	A8456
2-mercaptoethanol	Sigma-Aldrich	M3148
Glycerol	VWR Chemicals	24388.295
GTP	Sigma-Aldrich	G8877
PBS	Thermo Fisher Scientific	14190169
Carboxypeptidase A (CPA)	Sigma-Aldrich	C9268
SDS	VWR Chemicals	442444H
DTT	Sigma-Aldrich	D9779
Bromophenol blue	Sigma-Aldrich	1.08122
Tris (Trizma base)	Sigma-Aldrich	T1503
HCl	VWR Chemicals	20252.290
TWEEN 20	Sigma-Aldrich	P1379
Skimmed milk powder	REGILAIT	
Clarity Western ECL	Bio-rad	1705061
Taxol	Sigma-Aldrich	T7191
GMPCPP	Jena Biosciences	NU-405S
DMSO	Sigma-Aldrich	D8418
Triton X-100	Sigma-Aldrich	T9284
HEPES	Sigma-Aldrich	H3375
KCl	Sigma-Aldrich	P9541
ATP	Sigma-Aldrich	A2383
Imidazole	Sigma-Aldrich	792527
Benzonase	Sigma-Aldrich	E1014
3C-protease	CF ProtProd of Czech Infrastructure for Integrative Structural Biology (CIISB)	
Sulfuric acid	VWR Chemicals	102765 G
Hydrogen Peroxide	Sigma-Aldrich	386790
Dichlorodimethylsilane	Sigma-Aldrich	440272
Trichloroethylene	VWR Chemicals	28735.292
Pluronic® F-127	Sigma-Aldrich	P2443
Glucose	Sigma-Aldrich	G7021
Casein	Sigma-Aldrich	C6905
Glucose Oxidase	Sigma-Aldrich	G2133
Catalase from bovine liver	Sigma-Aldrich	C40
Glutaraldehyde	Sigma-Aldrich	G5882
MES hydrate	Sigma-Aldrich	M8250
NaCl	Sigma-Aldrich	S9625
Paraformaldehyde	Electron Microscopy Sciences	15713
NaBH_4_	Thermo Fisher Scientific	429131000
ProLong Gold anti-fade reagent	Thermo Fisher Scientific	P36934
**Software**		
Fiji (ImageJ)	Schindelin et al, 2012	Version: 1.54p
GraphPad Prism	GraphPad Software	Version: Prism 11
NIS-Elements	Nikon	6.0.2
ZEN	Zeiss	3.13
Adobe Photoshop	Adobe	27.8.0
Adobe Illustrator	Adobe	30.6
**Other**		

### Mouse lines and genotyping

All animal experiments were conducted in accordance with the European Community (2010/63/UE) on the protection of animals used for scientific purposes. The use of these animals was approved by the ethics committee of the Institut Curie CEEA-IC #118 (authorisations no. 04395.03 and 37315-2022051117455434 v2 given by the National Authority) in compliance with the international guidelines. Both male and female mice aged between 2 and 8 months were used in this research.

*Ttll1*^*−/−*^*/Ttll7*^*−/−*^ (Genova et al, 2023), *Atat1*^*−/−*^ (Kalebic et al, 2013), and *Tubb3*^*−/−*^ (Latremoliere et al, 2018) mice have been previously described.

For genotyping, ear fragments were collected from mice during weaning. DNA was isolated from these tissues using a lysis buffer composed of 100 mM Tris–HCl pH 8.0, 200 mM NaCl, 5 mM EDTA, and 1% Triton X-100, supplemented with 0.1 mg/mL proteinase K. Samples were incubated at 56 °C for at least 4 h for digestion. Proteinase K was subsequently inactivated by heating the lysates at 95 °C for 10 min. The details of the PCR primers, amplification conditions, and expected product sizes for each mouse line are provided in Appendix Table S2.

### Tubulin purification from murine brains

Tubulin with specific posttranslational modifications was purified from mouse brains using temperature-dependent microtubule polymerisation-depolymerisation cycles. The detailed protocol is described in (Souphron et al, 2019). After sacrificing the mouse, the skulls were opened immediately to extract the brain. Homogenised the brains using Ultra-Turrax^®^ blender in ice-cold lysis buffer consisting of BRB80 (80 mM K-PIPES pH 6.0, 1 mM K-EGTA, 1 mM MgCl_2_), 1 mM β-mercaptoethanol, 1 mM PMSF, and 1× protease inhibitor cocktail composed of 20 μg/ml leupeptin, 20 μg/ml aprotinin and 20 μg/ml 4-(2-aminoethyl)-benzene sulfonyl fluoride. For every gramme of brain tissue, 2 ml of ice-cold lysis buffer was added.

After homogenisation, lysates are centrifuged at 112,000 × *g* for 30 min at 4 °C to obtain the first supernatant (SN1). The volume of SN1 was measured, and 1/200 of its volume of 0.2 M GTP and 1/2 volume of pre-warmed 100% glycerol were added. The mixture was incubated for 30 min at 30 °C for polymerisation and then centrifuged at 112,000 × *g* for 20 min at 30 °C. The supernatant was discarded, and the pellet (P2) was depolymerised by adding 1/10 of the SN1 volume of ice-cold BRB80. The pellet was left in this buffer for 5 min, followed by pipetting up and down for 20 min. The depolymerised microtubules were then centrifuged at 112,000 × *g* for 20 min at 4 °C. To the supernatant (SN3), pre-warmed 1 M PIPES and 100% glycerol were each added at a volume equal to that of SN3, along with 1/100 of the SN3 volume of 0.2 M GTP. The mixture was incubated for 30 min at 30 °C for polymerisation and then centrifuged at 112,000 × *g* for 20 min at 30 °C. For the second depolymerisation, the pellet was resuspended in 1/40 volume of the SN1 of ice-cold BRB80, and the same steps as the first depolymerisation were followed. The resulting supernatant (SN5) was collected, and 1/200 of its volume of 200 mM GTP and half its volume of pre-warmed 100% glycerol were added. This mixture was incubated for 30 min at 30 °C for polymerisation, followed by centrifugation at 112,000 × *g* for 20 min at 30 °C. For the final depolymerisation, 1/40 volume of the SN1 of ice-cold BRB80 was added to the pellet (P6) and incubated on ice for 5 min. The mixture was then pipetted up and down for 10 min, followed by centrifuging at 112,000 × *g* for 20 min at 4 °C. The concentration of tubulin in supernatant was determined using a NanoDrop ND-1000 spectrophotometer (Thermo Scientific; absorbance at 280 nm; MW = 110 kDa; ε = 115,000 M^−^¹cm^−^¹). The tubulin solution was diluted to 4 mg/mL, aliquoted, snap-frozen in liquid nitrogen, and stored at −80 °C.

Analyses of the PTMs and isotypes of brain tubulin showed the absence of the PTM or isotype for which the corresponding genes were knocked out. The levels of other PTMs were similar to their wild-type levels, except for acetylation, which was enriched in *Ttll1*^*−/−*^*Ttll7*^*−/−*^ brain tubulin as shown before (Genova et al, 2023). We also detected some decrease in β3-tubulin levels in *Ttll1*^*−/−*^*Ttll7*^*−/−*^ brain tubulin, as well as lower tyrosination signals in tubulin from *Atat1*^*−/−*^ and *Tubb3*^*−/−*^ brain, which might be related to slight variations in the loading of our gels, given that signals for two linked PTMs detyrosination and ∆2-tubulin were unchanged.

### Tubulin purification from HeLa S3 cells in suspension culture

Tyrosinated and detyrosinated tubulin were purified from HeLa S3 cells in suspension culture. HeLa S3 cells were grown in spinner bottles containing 1 L of DMEM supplemented with 10% foetal bovine serum, 200 mM L-glutamine, and 1× penicillin–streptomycin on a stirrer table in a cell culture incubator for 1 week. The detailed protocol for amplifying cells for growing them in suspension culture is described in (Souphron et al, 2019).

The cell suspension culture was collected in 1 L centrifuge bottles and centrifuged at 250 × *g* for 15 min at room temperature. The pellet was resuspended in a minimal volume of PBS and transferred into a 50-mL Falcon tube. The cells were then centrifuged again at 250 × *g* for 8 min at 4 °C. The supernatant was discarded, and the volume of cells was estimated, and an equal volume of ice-cold lysis buffer was added as described above. The cells were lysed using a French press at medium pressure. The lysate was then centrifuged at 112,000 × *g* for 30 min at 4 °C to obtain the first supernatant (SN1). The volume of SN1 was recorded, and it was transferred using a syringe and a long needle to another tube, avoiding the floating layer on the supernatant. To SN1, 1/200 of its volume of 200 mM GTP and 1/2 volume of pre-warmed 100% glycerol were added, and polymerisation was performed as previously described. The pellet (P2) was depolymerised by adding 1/60 of the SN1 volume of ice-cold BRB80. After centrifugation, the supernatant (SN3) was transferred to an Eppendorf tube for Carboxypeptidase A (CPA) treatment for detyrosination. SN3 was split into two equal samples ( + CPA and –CPA). To the portion for detyrosination, 1/300 of its volume of CPA (Sigma C9268; 5.56 U/mL) was added. Both samples were incubated at 30 °C for 5 min. The second polymerisation was conducted as previously described, followed by a second depolymerisation, to which 1/100 of the SN1 volume of ice-cold BRB80 was added, following the same steps as described above for the purification of tubulin from mouse brains. The third polymerisation was identical for both samples. For the final depolymerisation, 1/300 volume of the SN1 of ice-cold BRB80 was added to the pellet, and all subsequent steps, including dilution and storage, were performed similarly as described above.

### Immunoblotting

Purified tubulin samples were mixed with Laemmli buffer (4% SDS, 90 mM DTT, 80 mM Tris–HCl pH 6.8, 10% glycerol, Bromophenol blue) and denatured for 5 min at 95 °C. In all, 60–80 ng of purified tubulin per sample was run on a special type of 10% SDS-PAGE gel that allows for the separation of α- and β-tubulin (Magiera and Janke, 2013). Proteins were transferred onto a nitrocellulose membrane using the Bio-Rad Trans-Blot^®^ Turbo system. Membranes were incubated in 5% fat-free dry milk in PBS containing 0.1% Tween-20 (PBST) for 1 h at room temperature for blocking. Primary antibodies were diluted in 2.5% milk in PBST and incubated with the membrane at RT for 1.5 h. After three 5-min washes with PBST, membranes were incubated with secondary HRP-labelled antibodies diluted in PBST for 45 min at room temperature. Following three additional 5-min washes with PBST, membranes were developed by incubating with Clarity™ Western ECL Substrate for 1 min, and the chemiluminescence signal was detected using the Vilber Fusion FX imaging system. All antibodies and dilutions used are listed in Appendix Table S3.

### Preparation of stabilised microtubules

For microtubule-MAP binding experiments, Taxol-stabilised microtubules were prepared following a previously described protocol (Braun et al, 2011; Nitzsche et al, 2010). The polymerisation mixture was prepared by combining 25% DMSO, 20 mM MgCl_2_, and 5 mM GTP in BRB80 on ice. 1.25 μl of this mixture was added to 5 μl of 4 mg/mL purified tubulin, and the sample was incubated on ice for 5 min. It was then incubated at 37 °C for 30 min for polymerisation. After polymerisation, 100 μL of BRB80T (10 μM Taxol in BRB80) was added to the microtubule mix and gently mixed. The reaction was then centrifuged at 18,000 ×* g* for 30 min at room temperature, the supernatant was discarded, and the pellet was resuspended in 100 μL BRB80T using a cut-off pipette tip to prevent the breaking of long microtubules.

For Tau and MAP2 binding assays, microtubules were polymerised using the slowly hydrolysing GTP analogue GMPCPP (Guanosine-5′-[(α,β)-methyleno]triphosphate; Jena Bioscience NU-405S). Purified tubulin was mixed with 1 mM GMPCPP and 1 mM MgCl_2_ to a final concentration of 2 μM in BRB80, and incubated for 7-8 h at 37 °C. After polymerisation, the microtubules were centrifuged at 18,000 × *g* for 30 min at room temperature. The supernatant was discarded, and the pellet was resuspended in 120 μL of BRB80T using a cut-off pipette tip.

### Generation of expression vectors and preparation of lysates from mammalian cells

The preparation of cell lysates containing MAPs from HEK-293 (Human embryonic kidney; ATCC CRL-1573) cells has been described in detail in our previously published paper (Jijumon et al, 2024). We cloned the open reading frames of MAPs into the mammalian expression vector pTRIP (Gentili et al, 2015) in frame with the fluorescent protein of our choice using the Sequence and Ligation Independent Cloning (SLIC) method (Jeong et al, 2012). Most clones have been described in our previous work (Jijumon et al, 2022). For SNPH, the human splice isoforms characterised in a previous study (Caino et al, 2017) differ by the presence of an additional stretch of 44 amino acids that is absent from both murine isoforms identified here. For MAP1A, our clone amplified from murine brain cDNA differs in several codons from the available published sequence NM_032393 with 60 bp insertion at pos. 7614 and 141 bp lacking at pos. 8314.

In total, 10^6^ HEK-293 cells were seeded on a 22-cm^2^ dish for transfection and grown in a 37 °C cell culture incubator. Expression plasmids encoding GFP-tagged MAPs were transfected using jetPEI (Polyplus #101000020) when the cells reached ~80% confluency. For each dish, 5 μg of DNA and 10 μl of jetPEI were mixed in serum-free DMEM containing 2 mM L-glutamine and 1× PenStrep.

After verifying GFP expression levels in HEK-293 cells using a fluorescence microscope, the cells were detached from the dish with trypsin, collected in DMEM, and centrifuged at 453 × *g* for 10 min at 4 °C. The supernatant was discarded, and for each dish of cells, 20–30 µL of freshly prepared ice-cold lysis buffer (HEPES buffer (50 mM K-HEPES pH 7.3, 1 mM EGTA and 2 mM MgCl_2_) containing 0.05% Triton-X100 and protease inhibitors (10 μg/ml leupeptin, aprotinin and 4-(2-aminoethyl)- benzene sulfonyl fluoride)) was added. The cell pellets were resuspended on ice for 10 min, and the samples were sonicated with three short pulses using a 6.5-mm probe output control set at 1 and duty cycle set at 10% (Branson Sonifier 450, model no. B450-WP). The samples were centrifuged at 33,800 ×* g* for 25 min at 4 °C, supernatants were collected, aliquoted into 5–10 μL samples and snap-frozen in liquid nitrogen, and stored at −80 °C.

### Purification of EML proteins

Full-length mouse EML proteins C-terminally tagged with mNeonGreen-3C-protease-cleavage-site followed by a histidine tag were expressed in SF9 insect cells using the open-source FlexiBAC baculovirus vector system (Lemaitre et al, 2019). Cells were lysed in 50 mM HEPES buffer pH 7.3, containing 300 mM KCl, 2 mM MgCl_2_, 5% glycerol, 0.1% Tween-20, 1 mM EGTA, 1 mM DTT, 0.1 mM ATP, 30 mM imidazole, Protease Inhibitor Cocktail (complete, EDTA-free, Roche), 25 units/ml benzonase. The cell lysate was centrifuged at 30,000 × *g* for 1 h at 4 °C. The supernatant was collected and incubated with Ni-NTA beads at 4 °C for 1 h on a rotator. Then loaded the bead-extract mixture onto the column. The column was washed with lysis buffer first and then with 50 mM HEPES buffer pH 7.4, containing 300 mM KCl, 2 mM MgCl_2_, 5% glycerol, 0.1% Tween-20, 2 mM EGTA, 1 mM DTT, 0.1 mM ATP, and 60 mM imidazole. Then we added 3 ml of 50 mM HEPES buffer pH 7.4, containing 300 mM KCl, 2 mM MgCl_2_, 5% glycerol, 0.1% Tween-20, 2 mM EGTA, 1 mM DTT, 0.1 mM ATP, 60 mM imidazole, and 3C protease to the column and incubated overnight for cleavage of the histidine tag. We then collected the flow through which contains the overexpressed protein of interest the next day and concentrated the protein in an Amicon centrifugal filter 50 K (Millipore) to a final volume of 500 μL. Protein was aliquoted to smaller aliquots, flash frozen, and stored at −80 °C.

### Preparation of microscopy chambers and microtubule–MAP binding assay

To prepare the microscopy chambers, we need two types of glass coverslips (22 × 22 mm^2^ and 18 × 18 mm^2^, Marienfeld High Precision). We cleaned the coverslips in Piranha solution, which was prepared by mixing 30% hydrogen peroxide (H_2_O_2_) and concentrated sulfuric acid (H_2_SO_4_) in a 1:2.5 ratio by volume. We silanised the coverslips by immersing them in a solution of 200 μL dichlorodimethylsilane (DDS) mixed in 350 mL trichloroethylene for 1 h at room temperature.

To prepare the flow chamber for microscopy, we attached two cleaned and silanised coverslips using parafilm strips, which had been heated for 20 s at 62 °C and lightly compressed, to form parallel flow chambers of about 3 mm width. Then we incubated each channel with 30 μg/ml monoclonal anti-β-tubulin antibody (Sigma-Aldrich T7816) for 10 min at room temperature to attach the microtubules to the coverslip surface. Then, we blocked the coverslip surface with 1% Pluronic-F127 in BRB80 for 45 min. We washed the channels with BRB80T (10 μM Taxol in BRB80) and introduced stabilised microtubules into the channel. For all assays, two different types of modified microtubules were added sequentially to each channel. We injected the first type of microtubules and incubated them in the channel for ~1 min, after which unbound microtubules were removed by flushing BRB80T. The microtubules were then imaged at several positions in the measurement chamber using interference reflection microscopy (IRM) using a 50:50 UVFS plate beamsplitter (ThorLabs BSW29R). The positions were saved using NIS-Elements imaging software (Nikon). We repeated the same steps for the second type of microtubules at the previously saved positions (Fig. 2B).

We equilibrated the channel with assay buffer (50 mM HEPES pH 7.3, 75 mM KCl, 20 mM glucose, 1 mM ATP, 10 mM DTT, 20 μg/mL casein, 220 μg/mL glucose oxidase, and 20 μg/mL catalase). We diluted MAP lysate in the assay buffer, flushed it into the channel with microtubules, and recorded MAP binding using Total Internal Reflection Fluorescence (TIRF) microscopy.

TIRF microscopy was performed using an inverted widefield microscope Nikon Eclipse Ti-E equipped with a motorised XY stage, Perfect Focus System (PFS), Nikon CFI SR Apo TIRF 100× Oil, NA 1.49, WD 0.12 mm objective and sCMOS Hamamatsu ORCA 4.0 V2 camera (Hamamatsu Photonics) or an inverted widefield Nikon Eclipse Ti-E equipped microscope equipped with motorised XY stage, PFS, Nikon Apo TIRF 60× Oil, NA 1.49, WD 0.12 mm objective and PRIME BSI sCMOS camera (Teledyne Photometrics). Movies were recorded with an exposure time of 100 ms or 50 ms for 5 min at a rate of 0.5 fps.

### Image analysis and statistical analysis

We analysed MAP binding to microtubules using Fiji (ImageJ) software (Schindelin et al, 2012). To compare the binding of MAPs to different PTM variants of microtubules, we calculated the density of fluorescent signal of the MAP-GFP on the respective microtubules. For that, we drew a segmented line of 5 pixels in width along the entire microtubule and measured the area (A) and the Raw Integrated Density (RID) of the fluorescent signal. We then increased the width of the line to 12 pixels and measured the same. We calculated the density of the background signal using the following equation:

$${{\rm{Background}}}({{\rm{B}}})=({{{\rm{RID}}}}_{12{{\rm{px}}}}-{{{\rm{RID}}}}_{5{{\rm{px}}}})/({{{\rm{A}}}}_{12{{\rm{px}}}}-{{{\rm{A}}}}_{5{{\rm{px}}}})$$

We then subtracted the background and determined the background-corrected density of MAP fluorescence signal (D) using the equation:

$${{\rm{D}}}=({{{\rm{RID}}}}_{5{{\rm{px}}}}-{{\rm{B}}}* {{{\rm{A}}}}_{5{{\rm{px}}}})/{{{\rm{A}}}}_{5{{\rm{px}}}}$$

Each experimental trial involved either wild-type microtubules from mouse brain tissue or wild-type HeLa cells, paired with one type of differentially modified microtubule. To facilitate comparisons, we standardised the mean D value of the wild-type microtubules to 1. Subsequently, the D values of the modified microtubules were normalised relative to this wild-type baseline, and the results were represented graphically. For that, we plotted these normalised values as scatter plots in Prism 10 software (GraphPad). Performed statistical analysis using an unpaired *t* test in Prism to measure *P* values.

### Immunostaining and imaging

Thirty thousand MDCK (ATTC CRL-2935) cells were seeded on coverslips in 24-well plates containing DMEM complete medium (supplemented with 10% FBS, 2 mM L-glutamine, and 1× penicillin–streptomycin). Cells were co-transfected with two different plasmids: one encoding VASH2 and the other encoding either EML1, EML2, EML3, or EML4. Co-transfection was performed using JetPEI (Polyplus#101000020) according to the manufacturer’s instructions, using 1 µg of each plasmid and a total of 4 µl of JetPEI per well. After 17 h, cells were fixed using a protocol designed to preserve the cytoskeleton while permeabilising the membrane.

Cells shown in Fig. 6 were pre-permeabilised for 2 min at 37 °C in 0.3% glutaraldehyde and 0.25% Triton X-100 in pre-warmed Cytoskeleton Buffer (10 mM MES (Sigma #M8250) pH 6.1, 150 mM NaCl, 5 mM EGTA, 5 mM glucose, 5 mM MgCl_2_), followed by fixation for 10 min at room temperature in 4% paraformaldehyde and 2% glutaraldehyde in Cytoskeleton Buffer. Cells shown in Fig. EV7 were permeabilised and fixed at the same time with 0.3% glutaraldehyde, 0.2% Triton X100 and 4% PFA in pre-warmed Cytoskeleton Buffer for 10 min at room temperature. To quench background fluorescence, cells were incubated for 7 min at room temperature in 0.1% NaBH_4_ in PBS. The cells were then blocked for 30 min in 5% bovine serum albumin (BSA) in PBS, followed by primary and secondary antibody incubations for 1 h each in 5% BSA in PBS. After each antibody incubation, we washed the cells three times with PBST (PBS + 0.1% Tween-20) at 5-min intervals. Coverslips were then mounted using ProLong Gold anti-fade reagent (Thermo Fisher Scientific). Imaging was performed using a Zeiss LSM980 confocal microscope with a 63× oil immersion objective, operated via Zen software, and the images were processed using Fiji and Adobe Photoshop. The antibodies used for immunostaining are listed in Appendix Table S4.

## Supplementary information

Appendix

Peer Review File

Source data Fig. 1

Source data Fig. 6

Figure EV3 Source Data

Figure EV4 Source Data

Figure EV5 Source Data

Figure EV6 Source Data

Appendix Figures Source Data

Expanded View Figures

## Data Availability

This study includes no data deposited in external repositories. The source data of this paper are collected in the following database record: biostudies:S-SCDT-10_1038-S44318-026-00908-8.
