# Supplementary material for: Microtubule posttranslational modifications provide unique recognition patterns for associated proteins
Source: EMBO J. 2026 Sep 12;45(20):7274–306. doi: 10.1038/s44318-026-00908-8 (PMC13672295; doi:10.1038/s44318-026-00908-8)
Supplement: Supplementary file 1 [file 44318_2026_908_MOESM10_ESM.pdf]

Expanded View Figures

Figure EV1. Amino acid sequence alignment of murine EML1-4 proteins.

Amino acid sequences of murine EML1, EML2, EML3, and EML4 proteins were aligned to identify conserved regions. Dark-coloured regions indicate conserved residues shared among the EML family members. Amino acid numbers refer to the consensus of the alignment. Above the aligned sequences: The orange-coloured box marks the amino-terminal (N-terminal) domains, while the blue-boxed region represents the carboxy-terminal (C-terminal) domains we used for cloning truncated and chimeric constructs used in Figs. EV3–EV6. The red-hatched box indicates the C-terminal domain unique to EML4 that was deleted in the construct used in Fig. EV6. *A previously determined domain structure of human EML1 (Richards et al, 2014) adapted to the current alignment is shown below the aligned sequences.

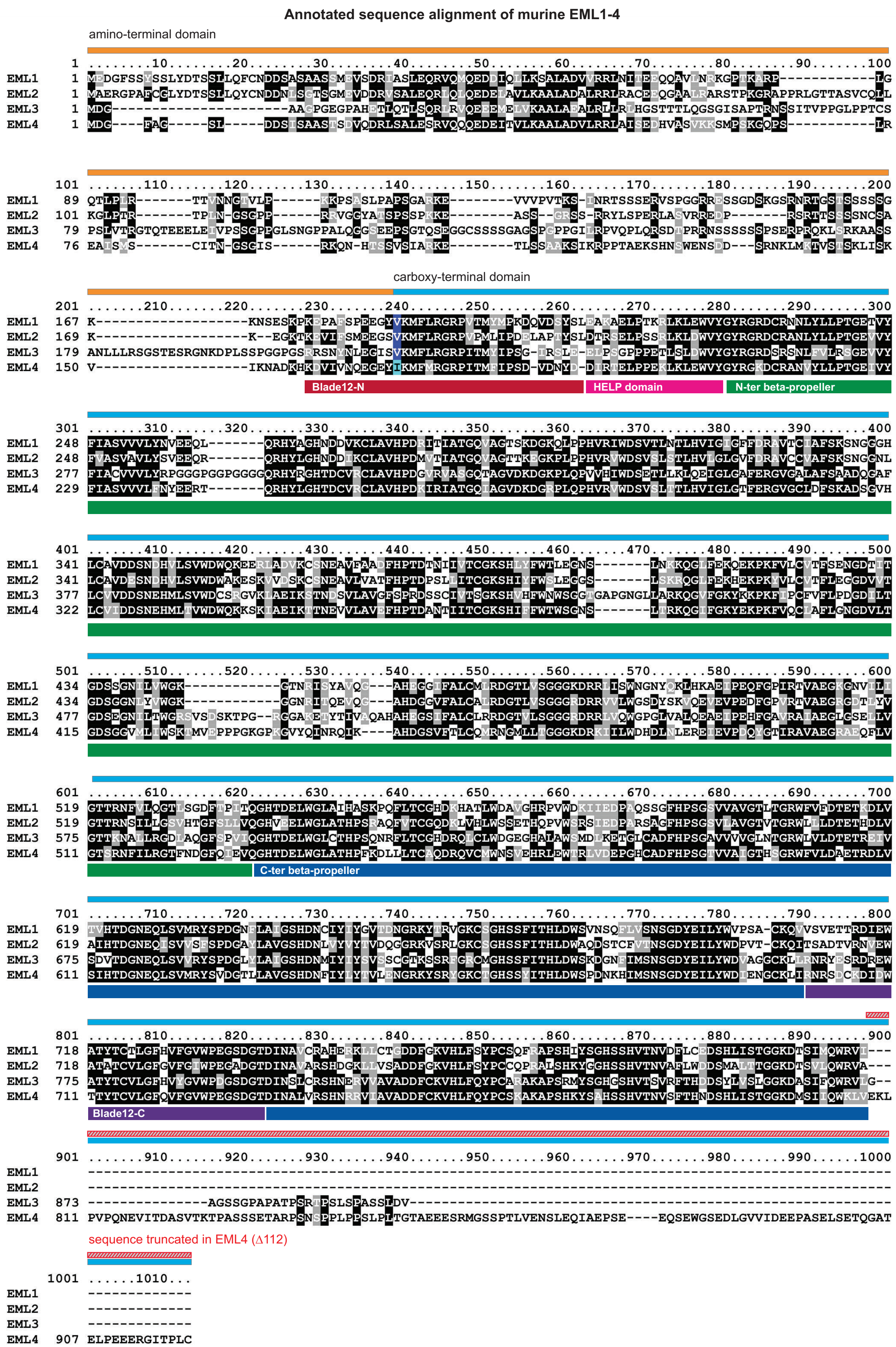
Annotated sequence alignment of murine EML1-4
amino-terminal domain
carboxy-terminal domain
Blade12-N
HELP domain
N-ter beta-propeller
C-ter beta-propeller
Blade12-C
sequence truncated in EML4 (Δ112)
EML1
EML2
EML3
EML4

 © The Author(s)

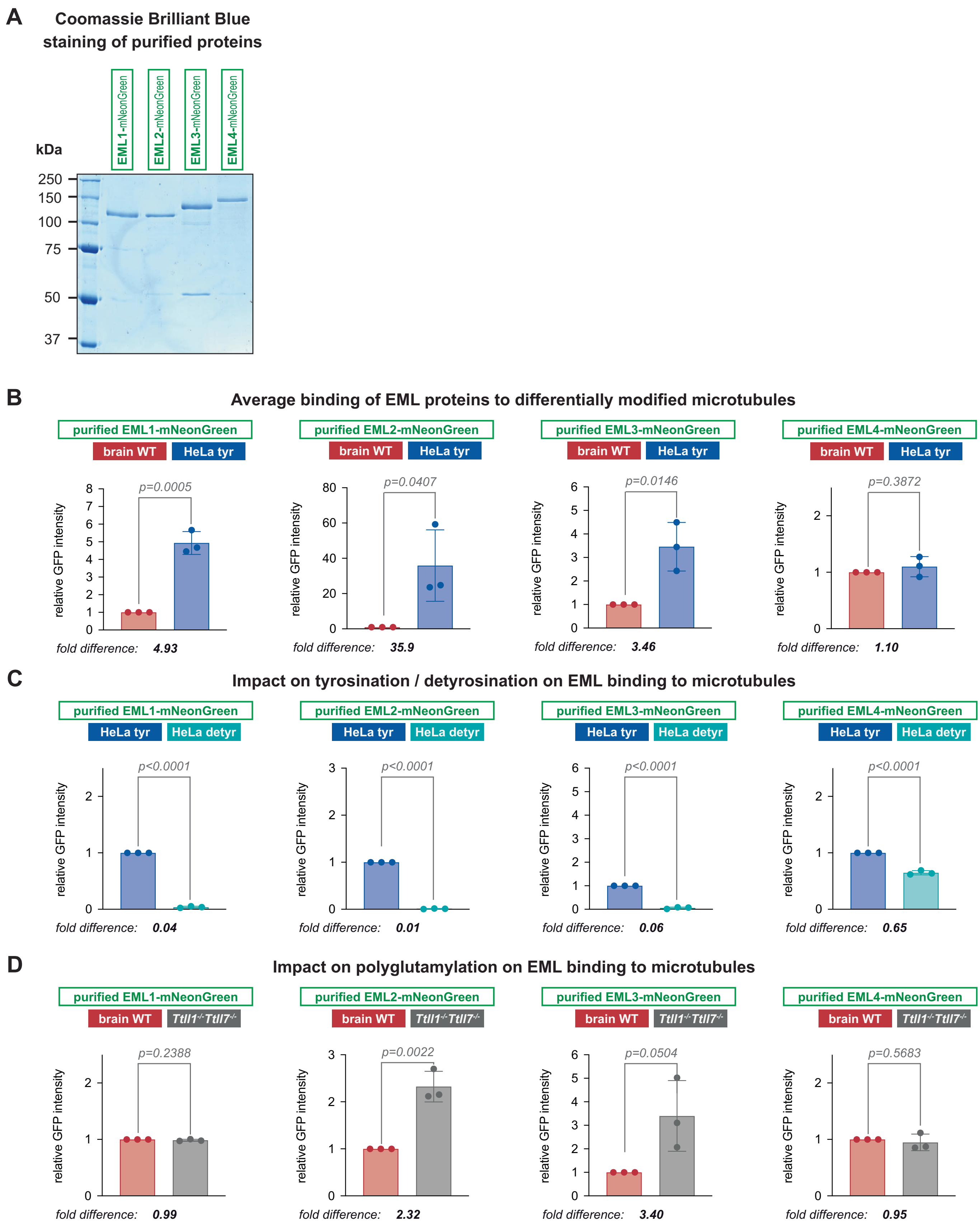
A
Coomassie Brilliant Blue staining of purified proteins
kDa
EML1-mNeonGreen
EML2-mNeonGreen
EML3-mNeonGreen
EML4-mNeonGreen
250
150
100
75
50
37
B
Average binding of EML proteins to differentially modified microtubules
purified EML1-mNeonGreen
brain WT
HeLa tyr
relative GFP intensity
p=0.0005
fold difference: 4.93
purified EML2-mNeonGreen
p=0.0407
fold difference: 35.9
purified EML3-mNeonGreen
p=0.0146
fold difference: 3.46
purified EML4-mNeonGreen
p=0.3872
fold difference: 1.10
C
Impact on tyrosination / detyrosination on EML binding to microtubules
HeLa tyr
HeLa detyr
purified EML1-mNeonGreen
p<0.0001
fold difference: 0.04
purified EML2-mNeonGreen
p<0.0001
fold difference: 0.01
purified EML3-mNeonGreen
p<0.0001
fold difference: 0.06
purified EML4-mNeonGreen
p<0.0001
fold difference: 0.65
D
Impact on polyglutamylation on EML binding to microtubules
brain WT
Ttll1-/-Ttll7-/-
purified EML1-mNeonGreen
p=0.2388
fold difference: 0.99
purified EML2-mNeonGreen
p=0.0022
fold difference: 2.32
purified EML3-mNeonGreen
p=0.0504
fold difference: 3.40
purified EML4-mNeonGreen
p=0.5683
fold difference: 0.95

© The Author(s)

Figure EV2. Comparative analysis of EML1-4 microtubule binding using purified proteins.

(**A**) Purified EML-mNeonGreen proteins were run on SDS-PAGE gels and stained with Coomassie Brilliant Blue. (**B–D**) Average of quantifications of microtubule binding of purified EML1-4-mNeonGreen. The complete datasets are represented for EML1 (plots from Appendix Fig. S26), EML2 (plots from Appendix Fig. S27), EML3 (plots from Appendix Fig. S28) and EML4 (plots from Appendix Fig. S29). (**B**) differential binding of EML proteins between wild-type brain (red) and HeLa (dark blue) microtubules. (**C**) Differential binding of EMLs to tyrosinated (dark blue) and detyrosinated (light blue) HeLa microtubules. (**D**) Differential binding of EMLs to polyglutamylated (red) and low-glutamylated (grey; *Ttll1*$^{-/-}$*Ttll7*$^{-/-}$) brain microtubules. Each data point represents the mean value from an independent assay shown in Appendix Figs. S26–29. Statistical significance was determined using unpaired *t* tests; *P* values are displayed above each comparison, and fold differences are shown below.

 © The Author(s)

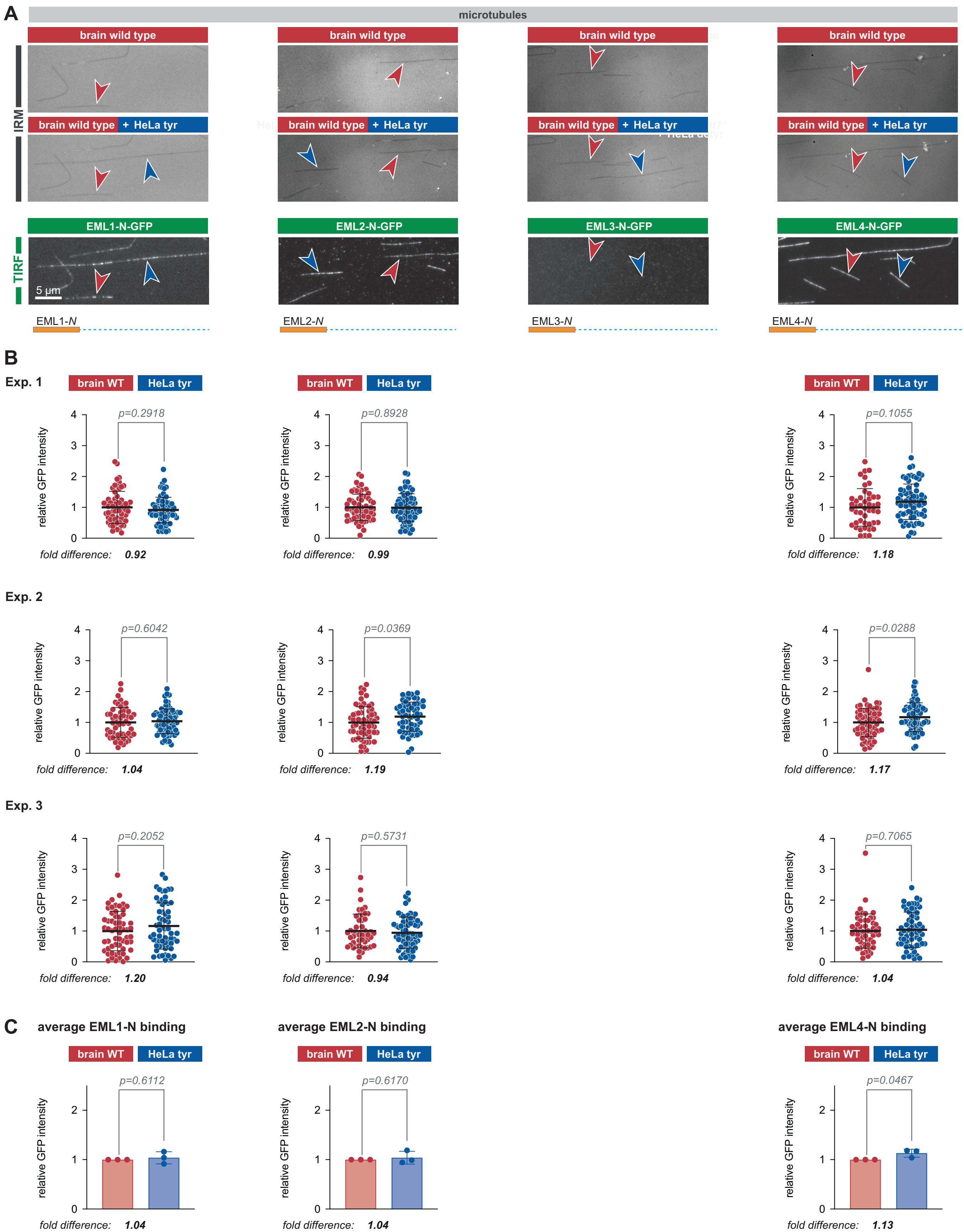
A
microtubules
brain wild type
IRM
brain wild type + HeLa tyr
EML1-N-GFP
EML2-N-GFP
EML3-N-GFP
EML4-N-GFP
TIRF
5 µm
EML1-N
EML2-N
EML3-N
EML4-N
B
Exp. 1
brain WT
HeLa tyr
relative GFP intensity
p=0.2918
fold difference: 0.92
p=0.8928
fold difference: 0.99
p=0.1055
fold difference: 1.18
Exp. 2
p=0.6042
fold difference: 1.04
p=0.0369
fold difference: 1.19
p=0.0288
fold difference: 1.17
Exp. 3
p=0.2052
fold difference: 1.20
p=0.5731
fold difference: 0.94
p=0.7065
fold difference: 1.04
C
average EML1-N binding
p=0.6112
fold difference: 1.04
average EML2-N binding
p=0.6170
fold difference: 1.04
average EML4-N binding
p=0.0467
fold difference: 1.13

Figure EV3. Binding of truncated EML1-4 amino termini to brain and HeLa microtubules.

(**A**) TIRF microscopy-based assay showing the amino-terminal domains of EML1-4 (EML1-*N*, 2-*N*, 3-*N*, 4-*N*; Fig. 5A) from cell lysates to Taxol-stabilised microtubules polymerised from either wild-type brain or wild-type HeLa tubulin. IRM images depict microtubules formed from brain and HeLa tubulin added sequentially, with corresponding snapshots and TIRF images displaying MAP binding. (**B**) Quantification of binding intensity from three independent assays. Each dot represents a single microtubule. EML3-N binding was too weak to be clearly visualised and therefore could not be quantified. (**C**) Average binding of the amino-terminal domains of EML1-4 to brain versus HeLa microtubules. Each data point represents the mean value from one assay. (**B**, **C**) Red denotes wild-type brain microtubules; dark blue denotes wild-type HeLa microtubules. Statistical significance was assessed using unpaired *t* tests; *P* values are indicated above each comparison, and fold changes are shown below. All experiments were performed in triplicate using independently purified tubulin and EML-*N* lysates prepared from three separate cell batches. Source data are available online for this figure.

 © The Author(s)

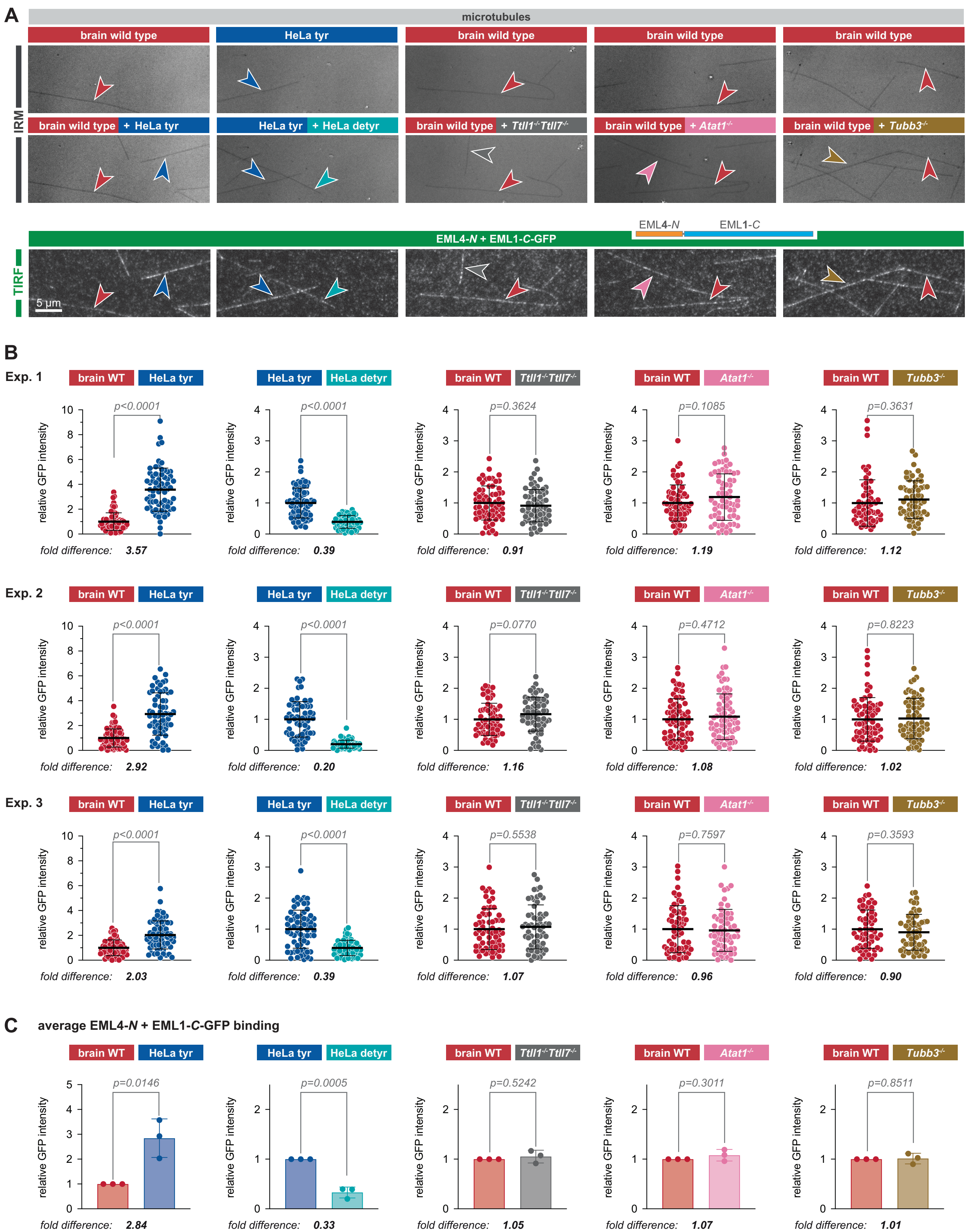
A
microtubules
brain wild type
HeLa tyr
brain wild type
brain wild type
brain wild type
IRM
brain wild type + HeLa tyr
HeLa tyr + HeLa detyr
brain wild type + Ttll1-/-Ttll7-/-
brain wild type + Atat1-/-
brain wild type + Tubb3-/-
EML4-N
EML1-C
EML4-N + EML1-C-GFP
TIRF
5 µm
B
Exp. 1
brain WT HeLa tyr
HeLa tyr HeLa detyr
brain WT Ttll1-/-Ttll7-/-
brain WT Atat1-/-
brain WT Tubb3-/-
relative GFP intensity
p<0.0001
p<0.0001
p=0.3624
p=0.1085
p=0.3631
fold difference: 3.57
fold difference: 0.39
fold difference: 0.91
fold difference: 1.19
fold difference: 1.12
Exp. 2
p<0.0001
p<0.0001
p=0.0770
p=0.4712
p=0.8223
fold difference: 2.92
fold difference: 0.20
fold difference: 1.16
fold difference: 1.08
fold difference: 1.02
Exp. 3
p<0.0001
p<0.0001
p=0.5538
p=0.7597
p=0.3593
fold difference: 2.03
fold difference: 0.39
fold difference: 1.07
fold difference: 0.96
fold difference: 0.90
C
average EML4-N + EML1-C-GFP binding
p=0.0146
p=0.0005
p=0.5242
p=0.3011
p=0.8511
fold difference: 2.84
fold difference: 0.33
fold difference: 1.05
fold difference: 1.07
fold difference: 1.01

Figure EV4. Binding of a chimaera of amino- and carboxy-terminal domains of EML4 and EML1 to microtubules with distinct tubulin PTMs and isotype compositions.

(**A**) TIRF microscopy-based assay showing the binding of a chimeric construct comprising the amino-terminal sequence of EML4 (EML4-*N*) fused to the carboxy-terminal sequence of EML1 (EML1-*C*; Fig. 5A) from cell lysates to Taxol-stabilised microtubules polymerised from tubulin sources with different PTMs and isotype compositions. IRM images show microtubules added sequentially, with corresponding snapshots and TIRF images displaying binding of the chimeric construct. (**B**) Quantification of EML4-*N* + EML1-*C*-GFP chimaera intensities in three independent assays. Fluorescence values are normalised to wild-type microtubules (either wild-type brain or wild-type HeLa). Each data point represents a single microtubule. Bars represent mean ± standard deviation. (**C**) Average binding of the chimeric EML4-*N* + EML1-*C*-GFP construct to different microtubule types. Each data point represents the mean value from one independent assay. (**B**, **C**) Red indicates wild-type brain microtubules; dark blue, wild-type HeLa; light blue, detyrosinated HeLa; grey, $Ttll1^{-/-}Ttll7^{-/-}$; pink, $Atat1^{-/-}$; and golden brown, $Tubb3^{-/-}$ mouse brain-derived microtubules. Statistical significance was determined using unpaired *t* tests; *P* values are shown above each comparison, and fold differences are indicated below. All experiments were performed in triplicate using independently purified tubulin and chimeric construct lysates prepared from three separate cell batches. Source data are available online for this figure.

 © The Author(s)

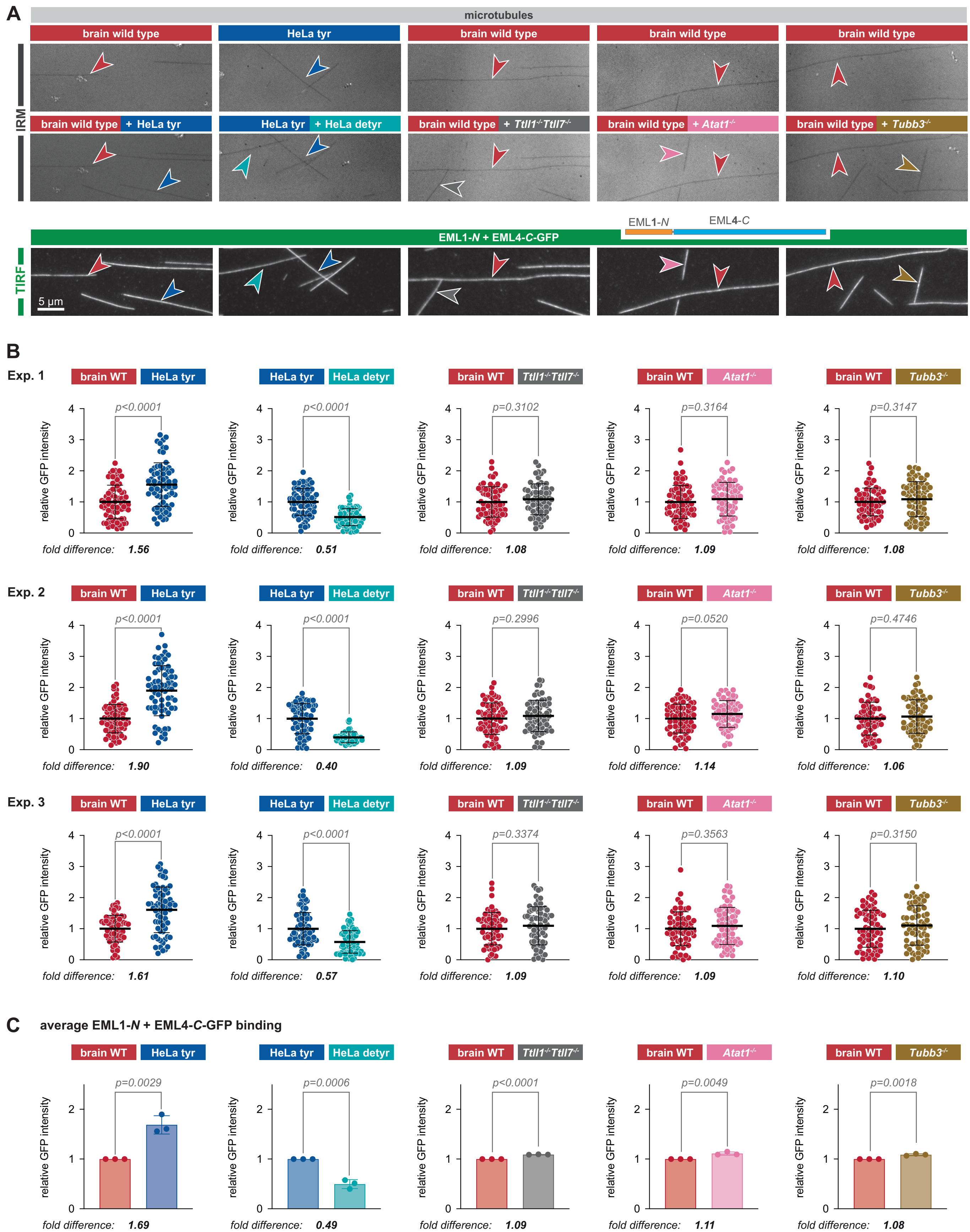
A
microtubules
brain wild type
HeLa tyr
IRM
brain wild type + HeLa tyr
HeLa tyr + HeLa detyr
brain wild type + Ttll1-/-Ttll7-/-
brain wild type + Atat1-/-
brain wild type + Tubb3-/-
EML1-N
EML4-C
EML1-N + EML4-C-GFP
TIRF
5 µm
B
Exp. 1
brain WT HeLa tyr
HeLa tyr HeLa detyr
brain WT Ttll1-/-Ttll7-/-
brain WT Atat1-/-
brain WT Tubb3-/-
relative GFP intensity
p<0.0001
p<0.0001
p=0.3102
p=0.3164
p=0.3147
fold difference: 1.56
fold difference: 0.51
fold difference: 1.08
fold difference: 1.09
fold difference: 1.08
Exp. 2
p<0.0001
p<0.0001
p=0.2996
p=0.0520
p=0.4746
fold difference: 1.90
fold difference: 0.40
fold difference: 1.09
fold difference: 1.14
fold difference: 1.06
Exp. 3
p<0.0001
p<0.0001
p=0.3374
p=0.3563
p=0.3150
fold difference: 1.61
fold difference: 0.57
fold difference: 1.09
fold difference: 1.09
fold difference: 1.10
C
average EML1-N + EML4-C-GFP binding
p=0.0029
p=0.0006
p<0.0001
p=0.0049
p=0.0018
fold difference: 1.69
fold difference: 0.49
fold difference: 1.09
fold difference: 1.11
fold difference: 1.08

Figure EV5. Binding of a chimaera of amino- and carboxy-terminal domains of EML1 and EML4 to microtubules with distinct tubulin PTMs and isotype compositions.

(**A**) TIRF microscopy-based assay showing the binding of a chimeric construct comprising the amino-terminal sequence of EML1 (EML1-*N*) fused to the carboxy-terminal sequence of EML4 (EML4-*C*; Fig. 5A) from cell lysates to Taxol-stabilised microtubules polymerised from tubulin sources with different PTMs and isotype compositions. IRM images show microtubules added sequentially, with corresponding snapshots and TIRF images displaying binding of the chimeric construct. (**B**) Quantification of EML1-*N* + EML4-*C*-GFP chimaera intensities in three independent assays. Fluorescence values are normalised to wild-type microtubules (either wild-type brain or wild-type HeLa). Each data point represents a single microtubule. Bars represent mean ± standard deviation. (**C**) Average binding of the chimeric EML1-*N* + EML4-*C*-GFP to different microtubule types. Each data point represents the mean value from one independent assay. (**B**, **C**) Red indicates wild-type brain microtubules; dark blue, wild-type HeLa; light blue, detyrosinated HeLa; grey, *Ttll1*$^{-/-}$*Ttll7*$^{-/-}$; pink, *Atat1*$^{-/-}$ and golden brown, *Tubb3*$^{-/-}$ mouse brain-derived microtubules. Statistical significance was determined using unpaired *t* tests; *P* values are shown above each comparison, and fold differences are indicated below. All experiments were performed in triplicate using independently purified tubulin and chimeric construct lysates prepared from three separate cell batches. Source data are available online for this figure.

 © The Author(s)

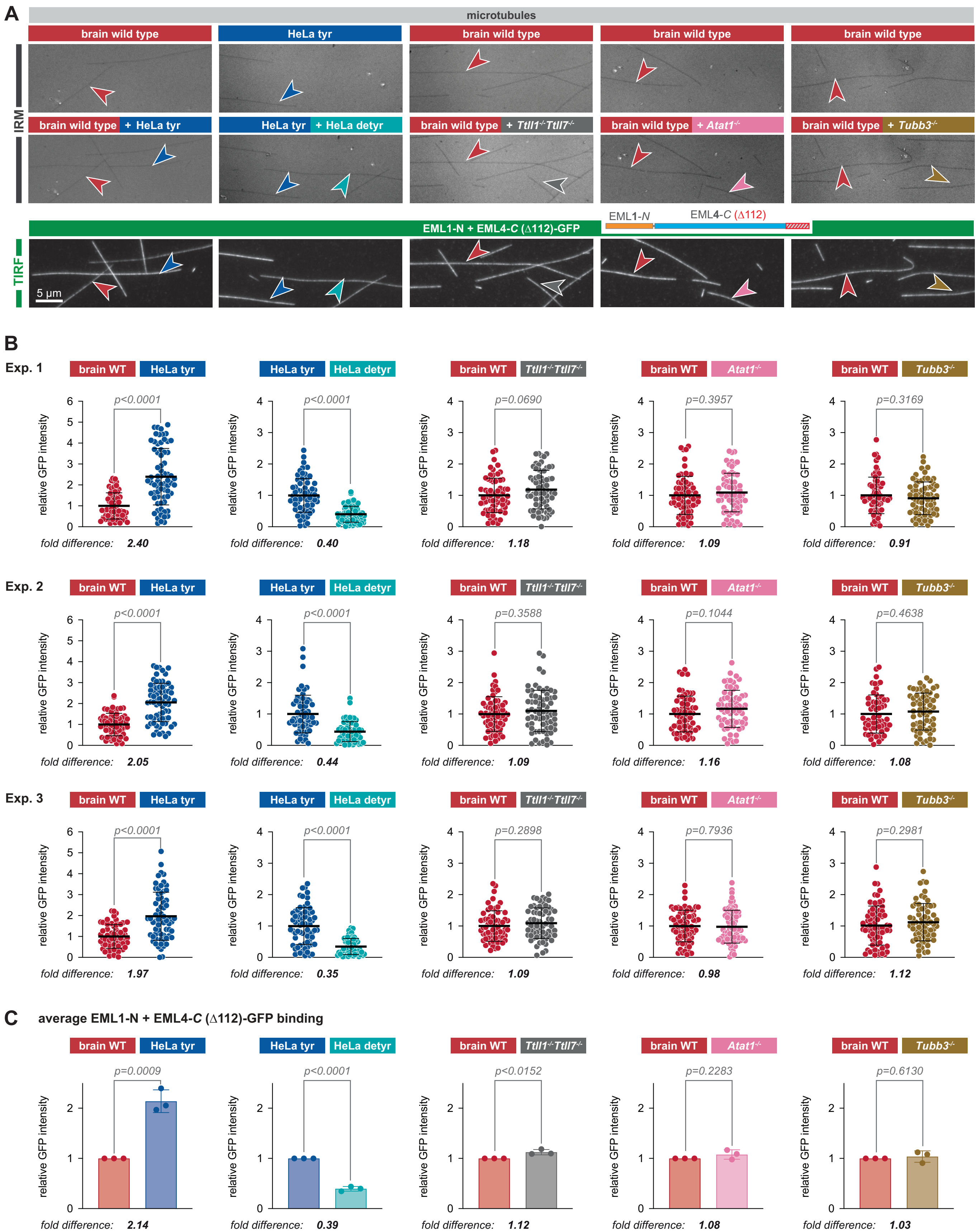
A
microtubules
brain wild type
HeLa tyr
brain wild type
brain wild type
brain wild type
IRM
brain wild type + HeLa tyr
HeLa tyr + HeLa detyr
brain wild type + Ttll1-/-Ttll7-/-
brain wild type + Atat1-/-
brain wild type + Tubb3-/-
EML1-N + EML4-C (Δ112)-GFP
EML1-N
EML4-C (Δ112)
TIRF
5 µm
B
Exp. 1
brain WT HeLa tyr
HeLa tyr HeLa detyr
brain WT Ttll1-/-Ttll7-/-
brain WT Atat1-/-
brain WT Tubb3-/-
relative GFP intensity
p<0.0001
p<0.0001
p=0.0690
p=0.3957
p=0.3169
fold difference: 2.40
fold difference: 0.40
fold difference: 1.18
fold difference: 1.09
fold difference: 0.91
Exp. 2
p<0.0001
p<0.0001
p=0.3588
p=0.1044
p=0.4638
fold difference: 2.05
fold difference: 0.44
fold difference: 1.09
fold difference: 1.16
fold difference: 1.08
Exp. 3
p<0.0001
p<0.0001
p=0.2898
p=0.7936
p=0.2981
fold difference: 1.97
fold difference: 0.35
fold difference: 1.09
fold difference: 0.98
fold difference: 1.12
C
average EML1-N + EML4-C (Δ112)-GFP binding
p=0.0009
p<0.0001
p<0.0152
p=0.2283
p=0.6130
fold difference: 2.14
fold difference: 0.39
fold difference: 1.12
fold difference: 1.08
fold difference: 1.03

Figure EV6. Binding of a chimaera of the amino-terminal domains of EML1 and the truncated carboxy-terminal domain of EML4 to microtubules with distinct tubulin PTMs and isotype compositions.

(**A**) TIRF microscopy-based assay showing the binding of a chimeric construct comprising the amino-terminal sequence of EML1 (EML1-*N*) fused to the carboxy-terminal sequence of EML4 lacking the unique 112 carboxy-terminal amino-acid stretch that is absent in EML1 (EML4-*C*(Δ112); Fig. 5A) from cell lysates to Taxol-stabilised microtubules polymerised from tubulin sources with different PTMs and isotype compositions. IRM images show microtubules added sequentially, with corresponding snapshots and TIRF images displaying binding of the chimeric construct. (**B**) Quantification of EML1-*N* + EML4-*C*(Δ112)-GFP chimaera intensities in three independent assays. Fluorescence values are normalised to wild-type microtubules (either wild-type brain or wild-type HeLa). Each data point represents a single microtubule. Bars represent mean ± standard deviation. (**C**) Average binding of the chimeric EML1-*N* + EML4-*C*(Δ112)-GFP construct to different microtubule types. Each data point represents the mean value from one independent assay. (**B**, **C**) Red indicates wild-type brain microtubules; dark blue, wild-type HeLa; light blue, detyrosinated HeLa; grey, *Ttll1*$^{-/-}$*Ttll7*$^{-/-}$; pink, *Atat1*$^{-/-}$ and golden brown, *Tubb3*$^{-/-}$ mouse brain-derived microtubules. Statistical significance was determined using unpaired *t* tests; *P* values are shown above each comparison, and fold differences are indicated below. All experiments were performed in triplicate using independently purified tubulin and chimeric construct lysates prepared from three separate cell batches. Source data are available online for this figure.

 © The Author(s)

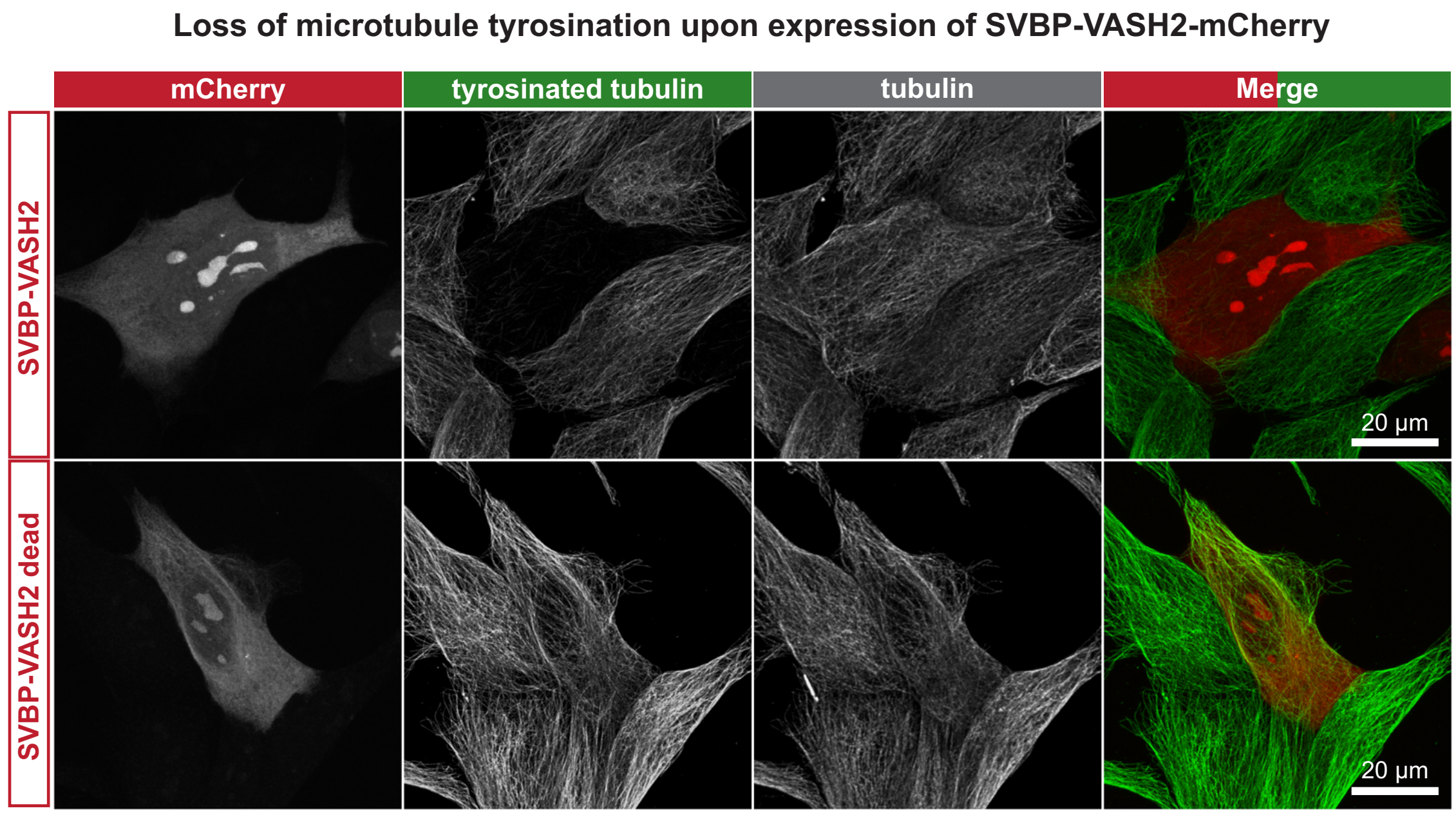

Figure EV7. Loss of tyrosination in cells expressing Vash2-Svbp.

Immunofluorescence staining of MDCK cells transiently transfected with either mCherry-tagged active Vash2-Svbp or with the catalytically inactive mutant of this enzyme. Cells were fixed and stained with anti-tyrosinated tubulin antibody (YL1/2). Note that cells expressing active, but not inactive Vash2-Svbp-mCherry, entirely lose YL1/2 labelling of microtubules, indicating full detyrosination. Scale bars: 20 µm.

© The Author(s)
