# Supplementary material for: Microtubule posttranslational modifications provide unique recognition patterns for associated proteins
Source: EMBO J. 2026 Sep 12;45(20):7274–306. doi: 10.1038/s44318-026-00908-8 (PMC13672295; doi:10.1038/s44318-026-00908-8)
Supplement: Supplementary file 2 [file 44318_2026_908_MOESM1_ESM.pdf]

APPENDIX TO:

Microtubule posttranslational modifications provide unique recognition patterns for associated proteins

Arya Krishnan, Eva Desvigne-Hansch, Veronique Henriot, Alica Dodoková, Sinda Khanfir, Mariya Genova, Lenka Grycova, Maria M. Magiera, Marcus Braun, Zdenek Lansky, Carsten Janke

<u>Includes:</u>

Appendix Tables S1-S4 .... pages 2-9

Appendix Figures S1-S29 .... pages 10-67

Appendix References .... pages 68-72

Appendix Table S1: MAPs and potential MAPs analysed in this study

Protein name	Accession number	Size (Aa)	GFP tag	Primer sequence (forward and reverse, 5'-3')	Back-bone	Species	Reference	In this study	Our names
ATIP3 (Angiotensin II type 2 (AT(2)) receptor interacting protein 3)	NP_001001924.2	270	N	human ATIP3 plasmid (Rodrigues-Ferreira et al, 2009)	pEGFP-C1	homo sapiens	(Di Benedetto et al, 2006; Rodrigues-Ferreira et al, 2009)	Appendix Fig. S2	X1666 GFP-ATIP3
CCDC66 (Coiled-coil Domain containing protein 66)	NM_17711	935	N	cggcatggacgagctgtacaagGGAAGC GGAGCTATGAACCTGGGAGATGGTTTAAAGCTTG; ggtcgacactagtggatccTTAAAACGAAGAACTAAAATTATCTTCTTGGTTTGC	pTRIP	mus musculus	(Conkar et al, 2017)	***no microtubule binding in our assays***	X1627 pSB186-1
CFAP20 (Cilia flagella associated protein 20)	XM_036153763.1	193	C	agaattattccgctagcATGTTCAAGAACACGTTCCAGAGTG; accatGGTGGCgctagcTTGTTTGGC CTTGTTCTGAACTGG	pTRIP	mus musculus	(Mendes Maia et al, 2014)	Appendix Fig. S2	X1422 pSB091-1
CKAP2 (Cytoskeleton associated protein 2)	BC145666.1	664	C	agaattattccgctagcATGGCAGAGTCCAGGAAACGCTTC; accatGGTGGCgctagcTTCCTCTTCCGCTACGTCGGTCTC	pTRIP	mus musculus	(Jin et al, 2004; Maouche-Chretien et al, 1998)	Appendix Fig. S2	X1757 pVH024-2
CKAP2L (Cytoskeleton associated protein 2-like)	BC053443.1	745	C	agaattattccgctagcATGGTGGGGCCCGGCCCCACTGC; accatGGTGGCgctagcTGATTCAAGGACTTCAAAGCCCAGTGTC	pTRIP	mus musculus	(Jakobsen et al, 2011; Yumoto et al, 2013)	Appendix Fig. S2	X1821 pVH070-1
CLIP170 (Cytoplasmic linker protein 170)	NM_001359221.2	1315	N	cggagtcgacACCATGAGTATGCTAAAACCCAGCGGCC; ccgcggatccTtAGAAGGTCTCGTCGTCATTGCAGTTGG	pTRIP	mus musculus	(Rickard & Kreis, 1990)	Appendix Fig. S5	X1521 pFM025-1
CSAP (Centriole, cilia and spindle-associated protein)	NM_028536.1	252	C	agaattattccgctagcATGTCCCCGGGCAGCGGGGTG; accatGGTGGCgctagcCGCCCTCGCTGAGTAGCACCG	pTRIP	mus musculus	(Ohta et al, 2015)	Appendix Fig. S6	X1536 pFM027-6
DCX (Doublecortin)	AY560329.1	365	C	agaattattccgctagcATGGAACTTGATTTTGGACATTTTGACGAAC; accatGGTGGCgctagcCATGGAATCGCCAAGTGAATCAGAGTC	pTRIP	mus musculus	(Francis et al, 1999; Gleeson et al, 1999)	Appendix Fig. S2	X1817 pVH069-1

EML1 (Echinoderm Microtubule associated protein like 1)	BC053094	814	C	agaattattccgctagcATGGAGGACGGCTTCTCCAGC; accatGGTGGCgctagcAATGACTCGCCACTGCATGATGC	pTRIP	mus musculus	(Eudy et al, 1997)	Appendix Fig. S7, S26	X1520 pFM024-2
EML2 (Echinoderm Microtubule associated protein like 2)	NM_001347582 (ATG pos. 275)	814	C	agaattattccgctagcATGGCGGAGCGCGGCCCGGCCTTC; accatGGTGGCgctagcGGCCACCCGCCACTGCAGCAC	pTRIP	mus musculus	Our cloning of murine homolog of long human EML2 isoform (Hotta et al, 2022; Lepley et al, 1999)	Appendix Fig. S8, S27	X2134 pVH319-1
EML3 (Echinoderm Microtubule associated protein like 3)	BC023878	897	C	agaattattccgctagcATGGACGGGGCTGCGGGGCCCGG; accatGGTGGCgctagcCACGTCCAGGGAGGAGGCAGGGG	pTRIP	mus musculus	(Tegha-Dunghu et al, 2008)	Appendix Fig. S9, S28	X1500 pSB119-7
EML4 (Echinoderm Microtubule associated protein like 4)	NM_001114362	919	C	agaattattccgctagcATGGACGGTTTCGCCGGCAGCC; accatGGTGGCgctagcGCACAGGGGTGTTATGCCTCGC	pTRIP	mus musculus	(Pollmann et al, 2006)	Appendix Fig. S10, S29	X1535 pFM026-1
GLFND1(fibronectin type 3 and SPRY domain containing protein)	NM_183178	496	N	cggcatggacgagctgtacaagGGAAGCGGAGCTATGGAAGACCAGAGGGAGGCGCTAAG; ggtcgacactagtggatcctTAGGTGAGGCTGGTATTGGAACTGC	pTRIP	mus musculus	homolog of human GLFND (Manabe et al, 2002)	Appendix Fig. S3	X1468 pFM011-3
GLFND2 (fibronectin type III and SPRY domain containing 1-like)	NM_001195284	497	N	cggcatggacgagctgtacaagGGAAGCGGAGCTATGGACTCGCAGAAAGAAGCTCTAC; ggtcgacactagtggatccTTACTGAGTGACATTGTTCAGGCTGC	pTRIP	mus musculus	homolog of human GLFND (Manabe et al, 2002)	Appendix Fig. S3	X1485 pSB098-1
HICE1/ HAUS8 (augmin-like complex, subunit 8)	NM_001163042.1	373	C	agaattattccgctagcATGGCGGATTCCTCAGAGCGGGAC; accatGGTGGCgctagcAGAGGGACTATCATCCCTGCAGGC	pTRIP	mus musculus	(Wu et al, 2008)	***no microtubule binding in our assays***	X1722 pVH005-1
JPT1 (Jupiter microtubule associated homologue 1)	NM_008258	154	N	cggcatggacgagctgtacaagGGAAGCGGAGCTATGACCACTACCACTACCTTTAAGGG; ggtcgacactagtggatcctTAACCCAAGACCAGGCTGGACTTG	pTRIP	mus musculus	(Karpova et al, 2006)	***no microtubule binding in our assays***	X1720 pVH003-4
JPT2 (Jupiter microtubule associated homolog 2)	NM_198937.2	190	N	cggcatggacgagctgtacaagGGAAGCGGAGCTATGTTCCAGGGCGCGGACAGCCAAG;	pTRIP	mus musculus	(Su et al, 2025)	***no microtubule binding in our assays***	X1721 pVH004-5

				ggtcgacactagtggatccTtAATAGAAGGAGAGGCTGGATTTGCC					
MACF1*(C 1023)* (Microtubule actin crosslinking factor 1 C-terminal 1023 amino acids)	AF150755	1023	C	agaattattccgctagcATGGAACTACATTCCCAGCTGAG; accatGGTGGCgctagcTCGCTTGGGACCTGGAGTCCTG	pTRIP	mus musculus	(Bernier et al, 1996; Leung et al, 1999)	Appendix Fig. S11	X1396 pSB057-1
MACF2*(C 1079)* (Microtubule actin crosslinking factor 2; Dystonin) C-terminal 1079 amino acids	NM_001276764	1079	C	agaattattccgctagcATGGAAGTCTGCACCGCCTTTG; accatGGTGGCgctagcTCTCTTCGAAGACTTGTCTAATTTG	pTRIP	mus musculus	(Bernier et al, 1995; Tamai et al, 1993)	Appendix Fig. S3	X1395 pSB056-1
MAP1A (Microtubule associated protein 1a)	our clone similar to NM_032393 with 60 bp insertion at pos. 7614 and 141 bp lacking at pos. 8314	2987	N	cgaattctgcagtcgacATGGCGACCGAAGCCGGGACCGCG; ctagatccggtggatcccgggctaGAACTCAATCTTGCAGGCTGGAAAGG	pTRIP	mus musculus	(Garner et al, 1990)	Appendix Fig. S12	X2211 pVH378-1
MAP1B (Microtubule associated protein 1B)	NM_008634.2	2464	N	murine MAP1B expression plasmid Addgene #44396 (Scales et al, 2009)	pTRIP	mus musculus	(Garner et al, 1990; Scales et al, 2009)	Appendix Fig. S13	X1891 GFP-mMAP1B
MAP2C (Microtubule associated protein 2 isoforms C)	BC051410	466	N	cggcatggacgagctgtacaagGGAAGCGGAGCTATGGCCGACGAGCGGAAAGATG; ggtcgacactagtggatccTtACAAGCCCTGCTTAGCAAGCG	pTRIP	mus musculus	(Garner et al, 1988)	Appendix Fig. S14	X1513 pFM018-1
MAP4_X16 (Microtubule associated protein 4 isoform X16 (new: 22))	NM_001428293	1012	N	cggcatggacgagctgtacaagGGAAGCGGAGCTATGGCCGACCTCAGTCTTGTGG; ggtcgacactagtggatccTtAATTTGTCTCCTGGATCTGGCTG	pTRIP	mus musculus	(Chapin et al, 1995)	Appendix Fig. S15	X1515 pFM019-1
MAP4_X20 (Microtubule associated protein 4 isoform X20 (new: 26))	NM_001428298	981	N	cggcatggacgagctgtacaagGGAAGCGGAGCTATGGCCGACCTCAGTCTTGTGG; ggtcgacactagtggatccTtAATTTGTCTCCTGGATCTGGCTG	pTRIP	mus musculus	(Chapin et al, 1995)	Appendix Fig. S16	X1516 pFM020-1

MAP6 (Microtubule associated protein 6)	NM_010837	906	C	agaattattccgctagcATGGCGTGGCCGTGCATCACAAG; accatGGTGGCgctagcAGGGGATCCCTCAATATATTCCGTATGG	pTRIP	mus musculus	(Bosc et al, 1996)	Appendix Fig. S17	X1723 pVH006-1
MAP7 (Microtubule associated protein 7)	NM_001428647.1	760	C	agaattattccgctagcATGCCTGGGTCAGCTACAGCGATCCG; accatGGTGGCgctagcTATAACTTCTGCGGTCTGTTGTGTCTGC	pTRIP	mus musculus	(Masson & Kreis, 1993)	Appendix Fig. S18	X1498 pSB123-1-1
MAP7D1 (MAP7 domain containing protein 1)	NM_001379092	809	C	agaattattccgctagcATGGAGAGTGGCCCGCGTGTGGAG; accatGGTGGCgctagcAAGAACTTCTGTGACCTGTGGAGGCTG	pTRIP	mus musculus	(Hooikaas et al, 2019; Kikuchi et al, 2022)	Appendix Fig. S19	X1502 pSB127-1
MAP7D2 (MAP7 domain containing protein 2)	NM_001081124	781	C	agaattattccgctagcATGGAGCGCAGCGGTGGGAACG; accatGGTGGCgctagcACAGAAGGT ATTCAGGGTAGTTTC	pTRIP	mus musculus	(Hooikaas et al, 2019; Kikuchi et al, 2022)	Appendix Fig. S3	X1517 pFM021-2
MAP7D3 (MAP7 domain containing protein 3)	NM_177293	876	C	agaattattccgctagcATGGCGGACCCCACTTTTGCTGCTAC; accatGGTGGCgctagcCACATCACTCTCTTCTGCCTCTTTTCC	pTRIP	mus musculus	(Hooikaas et al, 2019; Kikuchi et al, 2022; Sun et al, 2011)	Appendix Fig. S3	X1499 pSB125-2
MAP8 (Microtubule associated protein 8; MAP1C)	NP_766601	969	C	agaattattccgctagcATGGCGGCGCCGGAGGCTGTGGAG; accatGGTGGCgctagcGAACTCCACCTTGCAGGCGGGGAAG	pTRIP	mus musculus	(Orban-Nemeth et al, 2005; Wong et al, 2004)	Appendix Fig. S4	X1525 pSB137-1
MAP9 (Microtubule associated protein 9; ASAP)	NM_001081230	646	C	agaattattccgctagcATGTCCGATGAAATCTTCAGCACAAC; accatGGTGGCgctagcAAATACTTTTGAGGGCGCAGTTCTGC	pTRIP	mus musculus	(Saffin et al, 2005)	Appendix Fig. S4	X1538 pSB147-1
MAP10 (Microtubule associated protein 10)	NM_028908	891	C	agaattattccgctagcATGGCGACCACGGCGGCCGGGCGG; accatGGTGGCgctagcCACTGTGTATCCTGGAAGTTTATTTATTACTAACTCG	pTRIP	mus musculus	(Fong et al, 2013)	Appendix Fig. S4	X1478 pSB099-1
MAP11 (Microtubule associated protein 11)	our clone similar to XP_029389600.1	490		agaattattccgctagcATGGAGTCCCAATGCGACTACTCG; accatGGTGGCgctagcCTTGACGGGCTCCACCACCAGGAC	pTRIP	mus musculus	(Jijumon et al, 2022; Perez et al, 2019)	***no microtubule binding in our assays***	X1589 pSB174-3
MTCL1 (Microtubule cross-linking factor 1)	NM_172963	1946	C	mouse MTCL1 plasmid (Sato et al, 2013)	pGFP-C1	mus musculus	(Sato et al, 2013; Sato et al, 2014)	Appendix Fig. S20	X2012 pGFP-mMTCL1

NPHP1 (Nephrocystin 1)	NM_001291012.1	687	C	agaattattccgctagcATGCTCGCCCGGCGGCCGCGGGAC; accatGGTGGCgctagcGCCTGAGTTCTTTCTTATTTCACCCAAGAAG	pTRIP	mus musculus	(Dafinger et al, 2011; Datta et al, 2021)	***no microtubule binding in our assays***	X1878 pVH113-1
POC5 (centriolar protein)	NM_026173.3	558	N	cggcatggacgagctgtacaagGGAAGCGGAGCTATGTCATCTGATGAGGACAAGTG; ggtcgacactagtggatcctTAGTCAACCACTTTTATCGACTG	pTRIP	mus musculus	(Azimzadeh et al, 2009)	***no microtubule binding in our assays***	X1875 pVH110-1
PRC1 (Protein regulator of cytokinesis 1)	NM_145150	606	C	agaattattccgctagcATGAGGAGAAGTGAGGTGCTAGCAG; accatGGTGGCgctagcGGACTGGATGTTGGTTGAATTGAGGATTC	pTRIP	mus musculus	(Bieling et al, 2010; Mollinari et al, 2002)	Appendix Fig. S4	X1533 pSB143-1
SAXO1 (Stabilizer of axonemal microtubules 1)	NM_001081096	476	C	cggactcgagACCATGGGGAAGAGGACTTGCATCTGCAAC; ccgcggatccGGCTGACACTTCTAGTTCCTTCTGACC	pEYFP	mus musculus	(Dacheux et al, 2015)	***no microtubule binding in our assays***	X1114 pLV011-2
SNPH_A (Syntaphilin isoforms A, variant 1)	NM_198214	528	C	agaattattccgctagcATGGCCATGTCCCTGCAGGGAAGTAG; accatGGTGGCgctagcGAGCTGAGAGCCACCGGCTGTGCTG	pTRIP	mus musculus	(Kang et al, 2008)	Appendix Fig. S21	X1454 pFM006-3
SNPH_C (Syntaphilin isoforms C, variant 3)	NM_001291077	459	C	agaattattccgctagcATGGCCATGTCCCTGCAGGGAAGTAG; accatGGTGGCgctagcGAGCTGAGAGCCACCGGCTGTGCTG	pTRIP	mus musculus	(Kang et al, 2008)	Appendix Fig. S22	X1453 pFM006-1
SPAG5 (Sperm associated antigen 5)	NM_017407	934	C	agaattattccgctagcATGTGGAGGGTGAAAACACTGAATCTC; accatGGTGGCgctagcCTCGGGTGAAAGCACTCTTAACACTTC	pTRIP	mus musculus	(Mack & Compton, 2001)	***no microtubule binding in our assays***	X1887 pVH118-1
SPEF1 (Sperm flagellar protein 1)	NM_027641	234	C	agaattattccgctagcATGGCCATGTCCCTGCAGGGAAGTAG; accatGGTGGCgctagcCCGCTGCTTGCGCTCGGCCTGCTG	pTRIP	mus musculus	(Dougherty et al, 2005)	Appendix Fig. S4	X1762 pVH027-4
Tau*(0N-3R)* (Tau isoform with 3 microtubule binding repeats)	NM_001285456	341	N	cggcatggacgagctgtacaagGGAAGCGGAGCTATGGCTGACCCTCGCC; ggtcgacactagtggatccTtACAAACCCTGCTTGGCCAAGG	pTRIP	mus musculus	(Cleveland et al, 1977a; Cleveland et al, 1977b; Weingarten et al, 1975)	Appendix Fig. S23	X1511 pFM015-4
TRIM46 (Tripartite motif-containing protein 46)	NM_183037.2	759	N	mouse TRIM46 Addgene #176402 (van Beuningen et al, 2015)	pGW1-GFP	mus musculus	(van Beuningen et al, 2015)	Appendix Fig. S24	X2016 pGW-GFP-TRIM46-

TRAK1 (Trafficking kinesin binding protein 1)	NM_175114	939	N	cggcatggacgagctgtacaagGGAAGCGGAGCTATGGCATTGGCAATTCAGCTCAGGC; ggtcgacactagtggatccTtACCGCAAGCTAGTCTGTTTGGGG	pTRIP	mus musculus	(Canty et al, 2023; Henrichs et al, 2020)	***no microtubule binding in our assays***	X1495 pSB120-1
TRAK2 (Trafficking kinesin binding protein 2)	NM_172406	913	N	cggcatggacgagctgtacaagGGAAGCGGAGCTATGAGTCAATCCCAGAATGCCATTTTCAAATC; ggtcgacactagtggatccTtAATCTTCCTTCAGGATACCCATTTTGGG	pTRIP	mus musculus	(Canty et al, 2023; Henrichs et al, 2020)	***no microtubule binding in our assays***	X1452 pFM005-1

Appendix Table S2: Primers and PCR conditions for genotyping.

<table>
<tr><th rowspan="2">mouse strain</th><th rowspan="2">PCR N°</th><th rowspan="2">PCR primers</th><th rowspan="2">PCR product size</th><th colspan="2">denaturation</th><th colspan="2">annealing</th><th colspan="2">elongation</th><th rowspan="2">N° of cycles</th></tr>
<tr><th>time</th><th>temp</th><th>time</th><th>temp</th><th>time</th><th>temp</th></tr>
<tr><td rowspan="2">Ttll1$^{-/-}$</td><td>PCR1</td><td>ACTCGTTCTGTGGCACCCTGGC; TGTGGTCCGCTCAGGTGCCTCC</td><td>WT: 330 bp</td><td rowspan="8">15 s</td><td rowspan="8">94°C</td><td rowspan="8">15 s</td><td rowspan="8">60°C</td><td rowspan="8">40 s</td><td rowspan="8">68°C</td><td rowspan="8">30</td></tr>
<tr><td>PCR2</td><td>CAATGTGCTTGGCGGTTCAGGATCCC; GAACTCGACACCACCTGCAACCAACC</td><td>KO: 520 bp (WT: 1,500 bp)</td></tr>
<tr><td rowspan="2">Ttll7$^{-/-}$</td><td>PCR1</td><td>CGACCGAGAACCTAGCTACTGCTCATT; CGCTATGAAATAACCCTGATGCTGAAG</td><td>WT: 320 bp</td></tr>
<tr><td>PCR2</td><td>TTCCTCATGACTTCACACTCCTCTGTG; CGCTATGAAATAACCCTGATGCTGAAG</td><td>KO: 330 bp; (WT: 1,000 bp)</td></tr>
<tr><td rowspan="2">Atat1$^{-/-}$</td><td>PCR1</td><td>CCTCTCCCACTATTGTCTCTCATTATTG; GCAGGTGTACATGCAGATAGAGTACTC</td><td>WT: 230 bp</td></tr>
<tr><td>PCR2</td><td>TATGCCCTTGATGGTGTGTCCCTG; GCAGGTGTACATGCAGATAGAGTACTC</td><td>KO: 370 bp</td></tr>
<tr><td rowspan="2">Tubb3$^{-/-}$</td><td>PCR1</td><td>CCAGCTTTGCTTCTCACCAGCTC; CGCTTACTGAGCAAATCTTCGTCAGC</td><td>WT: 400bp</td></tr>
<tr><td>PCR2</td><td>GACTAGCAGGTGCCAGTTCTTAGTC; CGCTTACTGAGCAAATCTTCGTCAGC</td><td>KO: 300bp (WT: 2000 bp)</td></tr>
</table>

Appendix Table S3: Antibodies for immunoblot analyses

Antibody	Reference	Description	Dilution
12G10	Developed by J. Frankel and M. Nelson, obtained from the Developmental Studies Hybridoma Bank, developed under the auspices of the NICHD, and maintained by the University of Iowa	α-tubulin, mouse monoclonal	1:500
polyE	AdipoGen #AG-25B-0030	C-terminal polyglutamylate chains ≥3E, rabbit polyclonal (Rogowski et al, 2010)	1:5,000
6-11B-1	Sigma-Aldrich #T6793	K40 acetylation on α-tubulin, mouse monoclonal IgG2b (Piperno & Fuller, 1985)	1:2,000
Tuj1	Biolegend #801202	Anti-β3-tubulin (TubB3), mouse monoclonal IgG2a (Lee et al, 1990)	1:500
YL1/2	Merck-Millipore #MAB1864	tyrosinated α-tubulin C-terminus, rat monoclonal (Kilmartin et al, 1982; Wehland et al, 1984)	1:1,000
anti-detyrosinated tubulin	Merck-Millipore #AB3201	α-tubulin C-terminus lacking the last amino acid, rabbit polyclonal (Gundersen et al, 1984; Paturle-Lafanechere et al, 1994)	1:1,000
anti-Δ2-tubulin	AdipoGen #AG-25B-0044	α-tubulin C-terminus lacking the last two amino acids, rabbit polyclonal (Paturle-Lafanechere et al, 1994)	1:1,000
HRP-anti-mouse	Bethyl Laboratories #A90-516P	HRP-conjugated goat anti mouse	1:10,000
HRP-anti-rabbit	Bethyl Laboratories #A120-201P	HRP-conjugated goat anti rabbit	1:10,000
HRP-anti-rat	Bethyl Laboratories # A110-236P	HRP-conjugated goat anti rat	1:10,000

Appendix Table S4: Antibodies for immunostaining

Antibody	Reference	Description	Dilution
12G10	Developed by J. Frankel and M. Nelson, obtained from the Developmental Studies Hybridoma Bank, developed under the auspices of the NICHD, and maintained by the University of Iowa	α-tubulin, mouse monoclonal	1:1,000
YL1/2	Merck-Millipore #MAB1864	tyrosinated α-tubulin C-terminus, rat monoclonal (Kilmartin et al, 1982; Wehland et al, 1984)	1:4,000
anti-rat alexa 488	Molecular probes #A11006	Alexa 488-conjugated goat anti rat	1:1,000
anti-mouse alexa 405	Molecular probes #A31553	Alexa 405-conjugated goat anti mouse	1:1,000

Krishnan et al. Appendix Figure S1

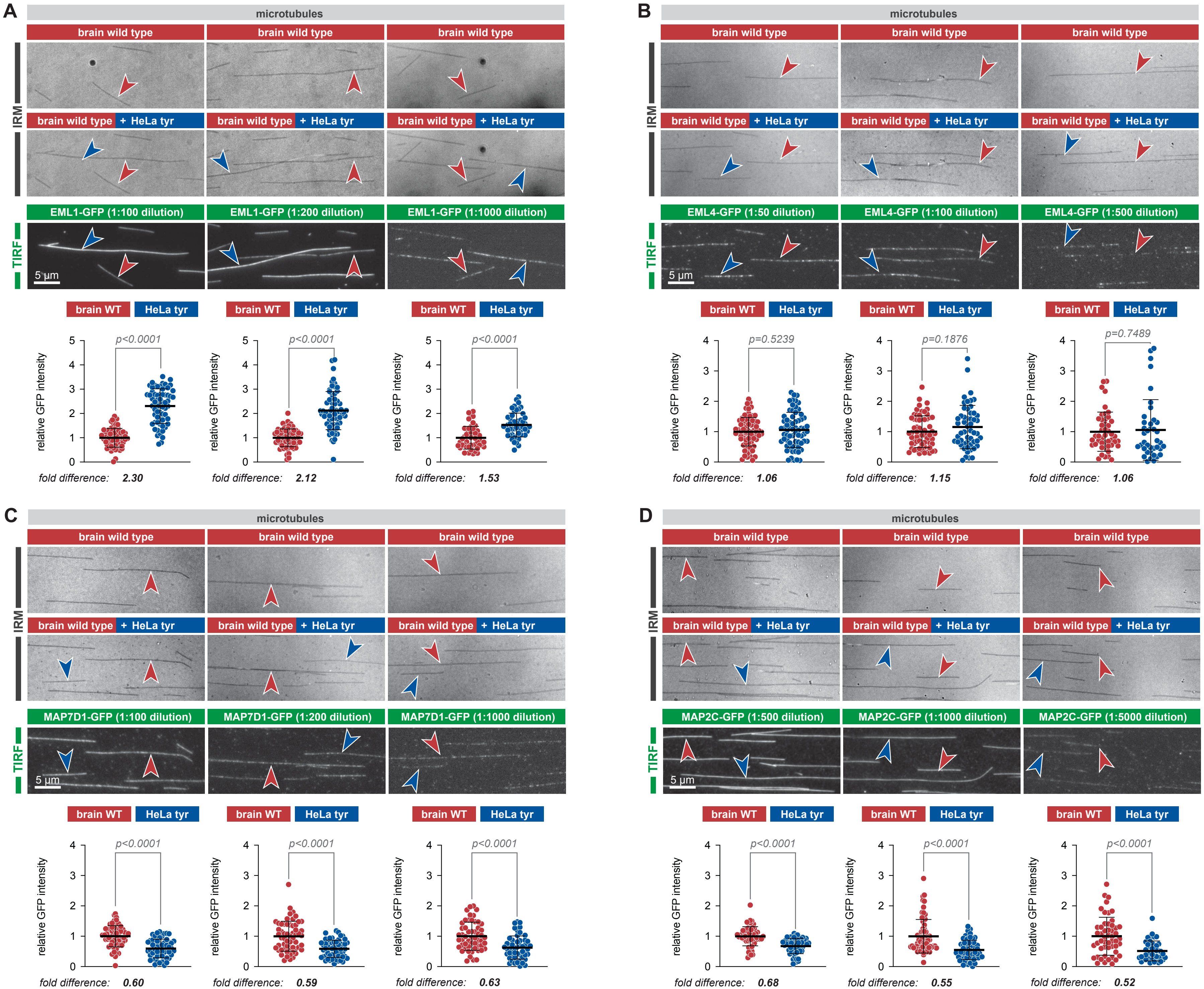

↑ Appendix Figure S1: MAPs retain their binding preferences across different lysate dilutions.

A-C) TIRF microscopy-based assay showing the binding of **A)** EML; **B)** EML4; **C)** MAP7D1 from cell lysates to Taxol-stabilised microtubules; or **D)** MAP2C to GMPCPP-stabilised microtubules polymerised from either wild-type brain or wild-type HeLa tubulin, using different dilutions of the cell extract. IRM images depict sequentially added brain and HeLa microtubules, with corresponding snapshots and TIRF images showing MAP-GFP binding. Quantification of binding intensity is shown alongside; each dot represents one microtubule. Red denotes wild-type brain microtubules; dark blue denotes wild-type HeLa microtubules. Statistical significance was assessed using unpaired t-tests; p-values are indicated above each comparison, and fold differences are shown below. All tested MAPs retain their binding preferences across all dilutions, with comparable fold-change values.

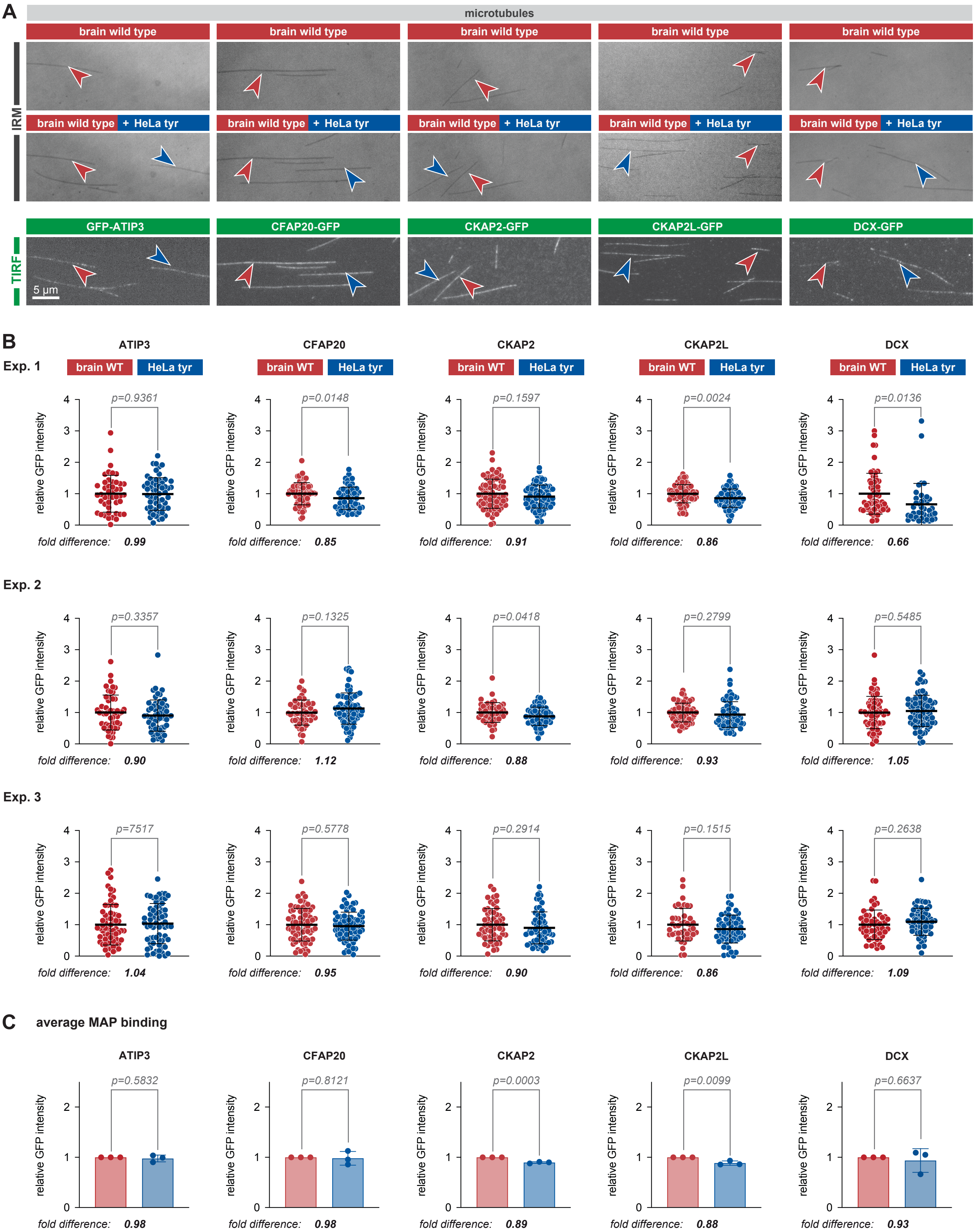

A
microtubules
brain wild type
brain wild type + HeLa tyr
IRM
GFP-ATIP3
CFAP20-GFP
CKAP2-GFP
CKAP2L-GFP
DCX-GFP
TIRF
5 µm
B
ATIP3
CFAP20
CKAP2
CKAP2L
DCX
Exp. 1
brain WT
HeLa tyr
relative GFP intensity
p=0.9361
p=0.0148
p=0.1597
p=0.0024
p=0.0136
fold difference: 0.99
fold difference: 0.85
fold difference: 0.91
fold difference: 0.86
fold difference: 0.66
Exp. 2
p=0.3357
p=0.1325
p=0.0418
p=0.2799
p=0.5485
fold difference: 0.90
fold difference: 1.12
fold difference: 0.88
fold difference: 0.93
fold difference: 1.05
Exp. 3
p=7517
p=0.5778
p=0.2914
p=0.1515
p=0.2638
fold difference: 1.04
fold difference: 0.95
fold difference: 0.90
fold difference: 0.86
fold difference: 1.09
C
average MAP binding
p=0.5832
p=0.8121
p=0.0003
p=0.0099
p=0.6637
fold difference: 0.98
fold difference: 0.98
fold difference: 0.89
fold difference: 0.88
fold difference: 0.93

🡡 Appendix Figure S2: Identification of MAPs that equally bind to brain or HeLa microtubules (1/3).

A) TIRF microscopy-based assay showing the binding of MAPs ATIP3, CFAP20, CKAP2, CKAP2L and DCX from cell lysates to Taxol-stabilised microtubules polymerised from either wild-type brain or HeLa tubulin. IRM images depict microtubules formed from brain and HeLa tubulin added sequentially, with corresponding snapshots and TIRF images displaying MAP binding.

B) Quantification of MAP-GFP intensities in three independent assays. Fluorescence values are normalised to wild-type microtubules (either wild-type brain or wild-type HeLa). Each data point represents a single microtubule. Bars represent mean ± standard deviation.

C) Average values of MAP binding to brain versus HeLa microtubules. Each data point represents the mean value from one assay. Red denotes wild-type brain microtubules; dark blue denotes wild-type HeLa microtubules. **(B,C)** Statistical significance was assessed using unpaired t-tests; p-values are indicated above each comparison, and fold changes are shown below. All experiments were conducted in triplicate using independently purified tubulin and separately prepared cell lysates from two independent batches.

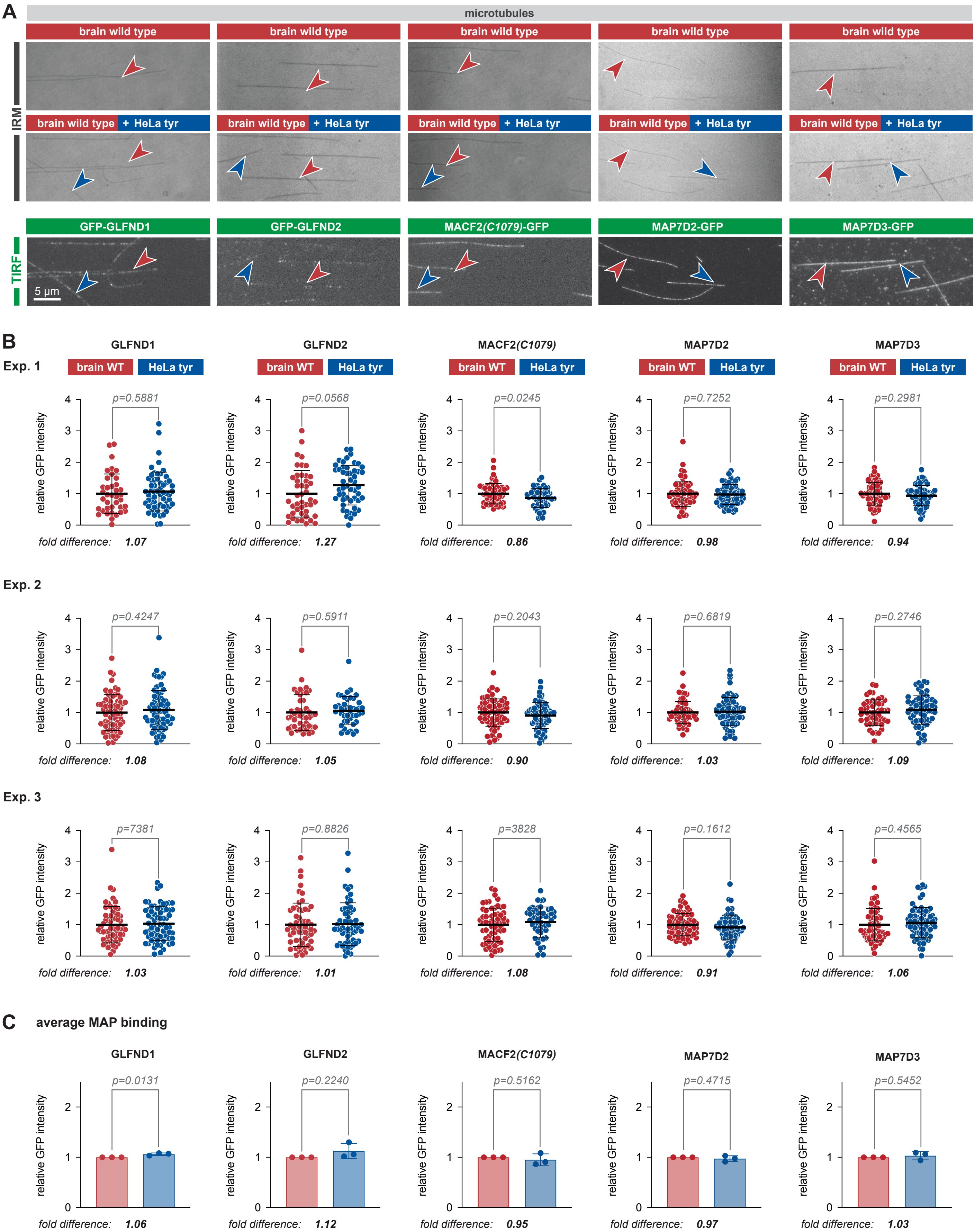
A
microtubules
brain wild type
IRM
brain wild type + HeLa tyr
TIRF
GFP-GLFND1
GFP-GLFND2
MACF2(C1079)-GFP
MAP7D2-GFP
MAP7D3-GFP
5 µm
B
GLFND1
GLFND2
MACF2(C1079)
MAP7D2
MAP7D3
Exp. 1
brain WT
HeLa tyr
relative GFP intensity
p=0.5881
p=0.0568
p=0.0245
p=0.7252
p=0.2981
fold difference: 1.07
fold difference: 1.27
fold difference: 0.86
fold difference: 0.98
fold difference: 0.94
Exp. 2
p=0.4247
p=0.5911
p=0.2043
p=0.6819
p=0.2746
fold difference: 1.08
fold difference: 1.05
fold difference: 0.90
fold difference: 1.03
fold difference: 1.09
Exp. 3
p=7381
p=0.8826
p=3828
p=0.1612
p=0.4565
fold difference: 1.03
fold difference: 1.01
fold difference: 1.08
fold difference: 0.91
fold difference: 1.06
C
average MAP binding
p=0.0131
p=0.2240
p=0.5162
p=0.4715
p=0.5452
fold difference: 1.06
fold difference: 1.12
fold difference: 0.95
fold difference: 0.97
fold difference: 1.03

↑ Appendix Figure S3: Identification of MAPs that equally bind to brain or HeLa microtubules (2/3).

A) TIRF microscopy-based assay showing the binding of MAPs GLFND1, GLFND2, MACF2*(C1079)*, MAP7D2, and MAP7D3 from cell lysates to Taxol stabilised microtubules polymerised from either wild-type brain or wild-type HeLa tubulin. IRM images depict microtubules formed from brain and HeLa tubulin added sequentially, with corresponding snapshots and TIRF images displaying MAP binding.

B) Quantification of MAP-GFP intensities in three independent assays. Fluorescence values are normalised to wild-type microtubules (either wild-type brain or wild-type HeLa). Each data point represents a single microtubule. Bars represent mean ± standard deviation.

C) Average values of MAP binding to brain versus HeLa microtubules. Each data point represents the mean value from one assay. Red denotes wild-type brain microtubules; dark blue denotes wild-type HeLa microtubules. **(B,C)** Statistical significance was assessed using unpaired t-tests; p-values are indicated above each comparison, and fold changes are shown below. All experiments were conducted in triplicate using independently purified tubulin and separately prepared cell lysates from two independent batches.

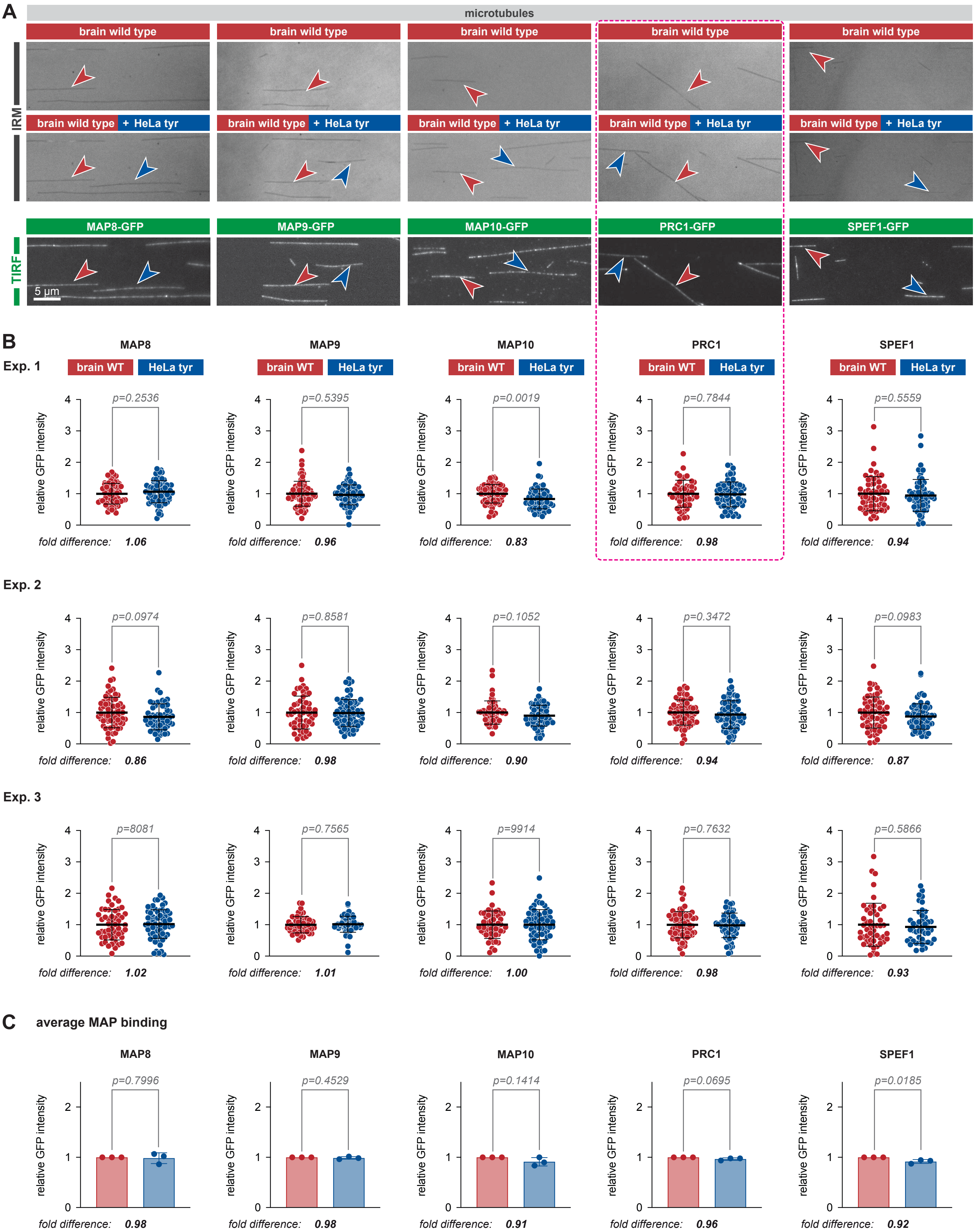
A
microtubules
brain wild type
brain wild type + HeLa tyr
IRM
MAP8-GFP
MAP9-GFP
MAP10-GFP
PRC1-GFP
SPEF1-GFP
TIRF
5 µm
B
MAP8
MAP9
MAP10
PRC1
SPEF1
Exp. 1
brain WT
HeLa tyr
relative GFP intensity
p=0.2536
p=0.5395
p=0.0019
p=0.7844
p=0.5559
fold difference: 1.06
fold difference: 0.96
fold difference: 0.83
fold difference: 0.98
fold difference: 0.94
Exp. 2
p=0.0974
p=0.8581
p=0.1052
p=0.3472
p=0.0983
fold difference: 0.86
fold difference: 0.98
fold difference: 0.90
fold difference: 0.94
fold difference: 0.87
Exp. 3
p=8081
p=0.7565
p=9914
p=0.7632
p=0.5866
fold difference: 1.02
fold difference: 1.01
fold difference: 1.00
fold difference: 0.98
fold difference: 0.93
C average MAP binding
MAP8
MAP9
MAP10
PRC1
SPEF1
p=0.7996
p=0.4529
p=0.1414
p=0.0695
p=0.0185
fold difference: 0.98
fold difference: 0.98
fold difference: 0.91
fold difference: 0.96
fold difference: 0.92

↑ Appendix Figure S4: Identification of MAPs that equally bind to brain or HeLa microtubules (3/3).

A) TIRF microscopy-based assay showing the binding of MAPs MAP8, MAP9, MAP10, PRC1 and SPEF1 from cell lysates to Taxol stabilised microtubules polymerised from either wild-type brain or wild-type HeLa tubulin. IRM images depict microtubules formed from brain and HeLa tubulin added sequentially, with corresponding snapshots and TIRF images displaying MAP binding.

B) Quantification of MAP-GFP intensities in three independent assays. Fluorescence values are normalised to wild-type microtubules (either wild-type brain or wild-type HeLa). Each data point represents a single microtubule. Bars represent mean ± standard deviation.

C) Average values of MAP binding to brain versus HeLa microtubules. Each data point represents the mean value from one assay. Red denotes wild-type brain microtubules; dark blue denotes wild-type HeLa microtubules. **(A,B)** Figure elements for PRC1 (dotted box) are also shown in Fig. 2B,C. **(B,C)** Statistical significance was assessed using unpaired t-tests; p-values are indicated above each comparison, and fold changes are shown below. All experiments were conducted in triplicate using independently purified tubulin and separately.

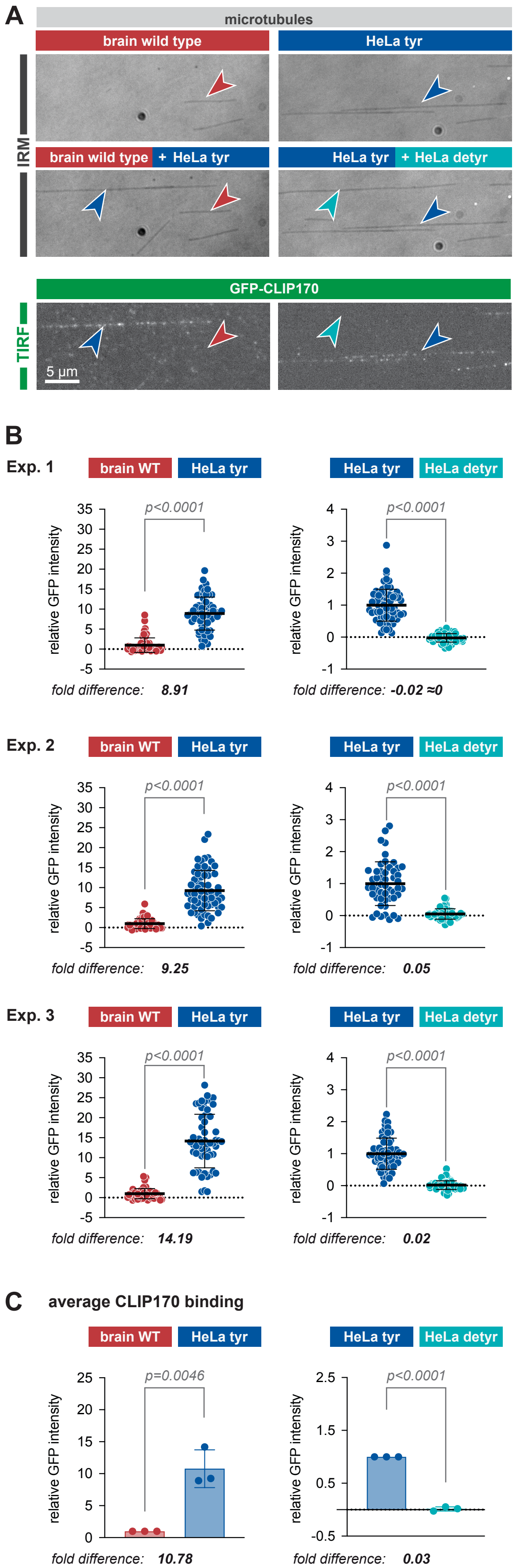
A
microtubules
brain wild type
HeLa tyr
IRM
brain wild type + HeLa tyr
HeLa tyr + HeLa detyr
GFP-CLIP170
TIRF
5 µm
B
Exp. 1
brain WT
HeLa tyr
HeLa tyr
HeLa detyr
relative GFP intensity
p<0.0001
fold difference: 8.91
fold difference: -0.02 ≈0
Exp. 2
fold difference: 9.25
fold difference: 0.05
Exp. 3
fold difference: 14.19
fold difference: 0.02
C
average CLIP170 binding
p=0.0046
p<0.0001
fold difference: 10.78
fold difference: 0.03

↑ Appendix Figure S5: CLIP170 binding to microtubules with distinct tubulin PTMs and isotype compositions.

A) TIRF microscopy-based assay showing CLIP170 binding from cell lysates to Taxol-stabilised microtubules polymerised from tubulin sources with different PTMs and isotype compositions. IRM images show microtubules added sequentially, with corresponding snapshots and TIRF images displaying CLIP170 binding.

B) Quantification of GFP-CLIP170 intensities in three independent assays. Fluorescence values are normalised to wild-type microtubules (either wild-type brain or wild-type HeLa). Each data point represents a single microtubule. Bars represent mean ± standard deviation.

C) Average CLIP170 binding to different microtubule types. Each data point represents the mean value from one independent assay. Red indicates wild-type brain microtubules; dark blue, wild-type HeLa; light blue, detyrosinated HeLa microtubules. **(B,C)** Red indicates wild-type brain microtubules; dark blue, wild-type HeLa; light blue, detyrosinated HeLa microtubules. Statistical significance was determined using unpaired t-tests; p-values are shown above each comparison, and fold differences are indicated below. Due to the very weak binding of CLIP170 to detyrosinated HeLa microtubules, some background-subtracted values were negative. These values were considered as zero for fold-difference calculations. All experiments were performed in triplicate using independently purified tubulin and CLIP170 lysates prepared from three separate cell batches.

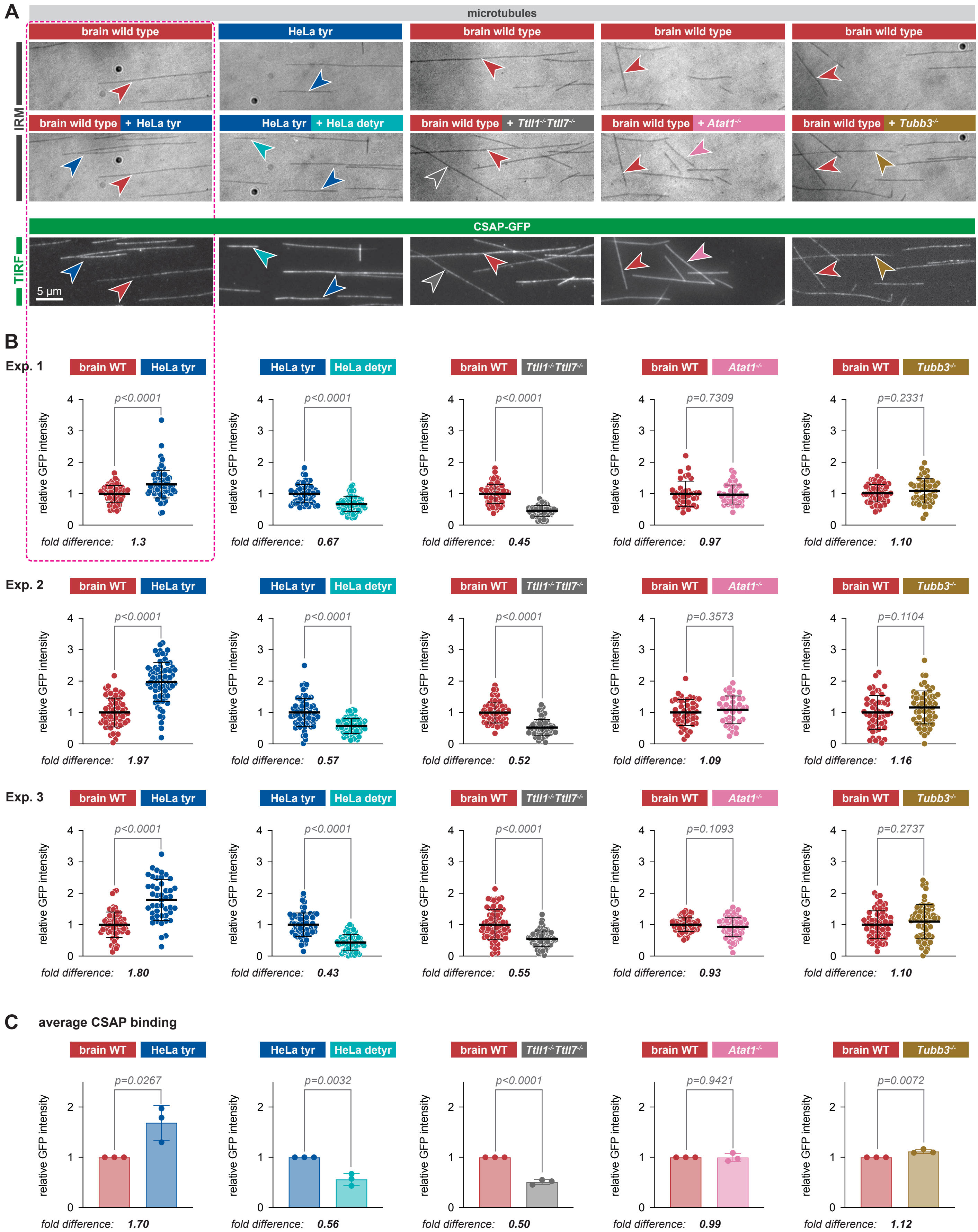
A
microtubules
brain wild type
HeLa tyr
brain wild type
brain wild type
brain wild type
IRM
brain wild type + HeLa tyr
HeLa tyr + HeLa detyr
brain wild type + Ttll1-/-Ttll7-/-
brain wild type + Atat1-/-
brain wild type + Tubb3-/-
CSAP-GFP
TIRF
5 µm
B
Exp. 1
brain WT HeLa tyr
p<0.0001
fold difference: 1.3
HeLa tyr HeLa detyr
p<0.0001
fold difference: 0.67
brain WT Ttll1-/-Ttll7-/-
p<0.0001
fold difference: 0.45
brain WT Atat1-/-
p=0.7309
fold difference: 0.97
brain WT Tubb3-/-
p=0.2331
fold difference: 1.10
relative GFP intensity
Exp. 2
p<0.0001
fold difference: 1.97
p<0.0001
fold difference: 0.57
p<0.0001
fold difference: 0.52
p=0.3573
fold difference: 1.09
p=0.1104
fold difference: 1.16
Exp. 3
p<0.0001
fold difference: 1.80
p<0.0001
fold difference: 0.43
p<0.0001
fold difference: 0.55
p=0.1093
fold difference: 0.93
p=0.2737
fold difference: 1.10
C
average CSAP binding
p=0.0267
fold difference: 1.70
p=0.0032
fold difference: 0.56
p<0.0001
fold difference: 0.50
p=0.9421
fold difference: 0.99
p=0.0072
fold difference: 1.12

↑ Appendix Figure S6: CSAP binding to microtubules with distinct tubulin PTMs and isotype compositions.

A) TIRF microscopy-based assay showing CSAP binding from cell lysates to Taxol-stabilised microtubules polymerised from tubulin sources with different PTMs and isotype compositions. IRM images show microtubules added sequentially, with corresponding snapshots and TIRF images displaying CSAP binding.

B) Quantification of CSAP-GFP intensities in three independent assays. Fluorescence values are normalised to wild-type microtubules (either wild-type brain or wild-type HeLa). Each data point represents a single microtubule. Bars represent mean ± standard deviation.

C) Average CSAP binding to different microtubule types. Each data point represents the mean value from one independent assay. **(A,B)** Figure elements in dotted box are also shown in Fig. 2B,C. **(B,C)** Red indicates wild-type brain microtubules; dark blue, wild-type HeLa; light blue, detyrosinated HeLa; grey, *Ttll1*$^{-/-}$*Ttll7*$^{-/-}$; pink, *Atat1*$^{-/-}$; and golden brown, *Tubb3*$^{-/-}$ mouse brain-derived microtubules. Statistical significance was determined using unpaired t-tests; p-values are shown above each comparison, and fold differences are indicated below. All experiments were performed in triplicate using independently purified tubulin and CSAP lysates prepared from three separate cell batches.

Krishnan et al. Appendix Figure S7

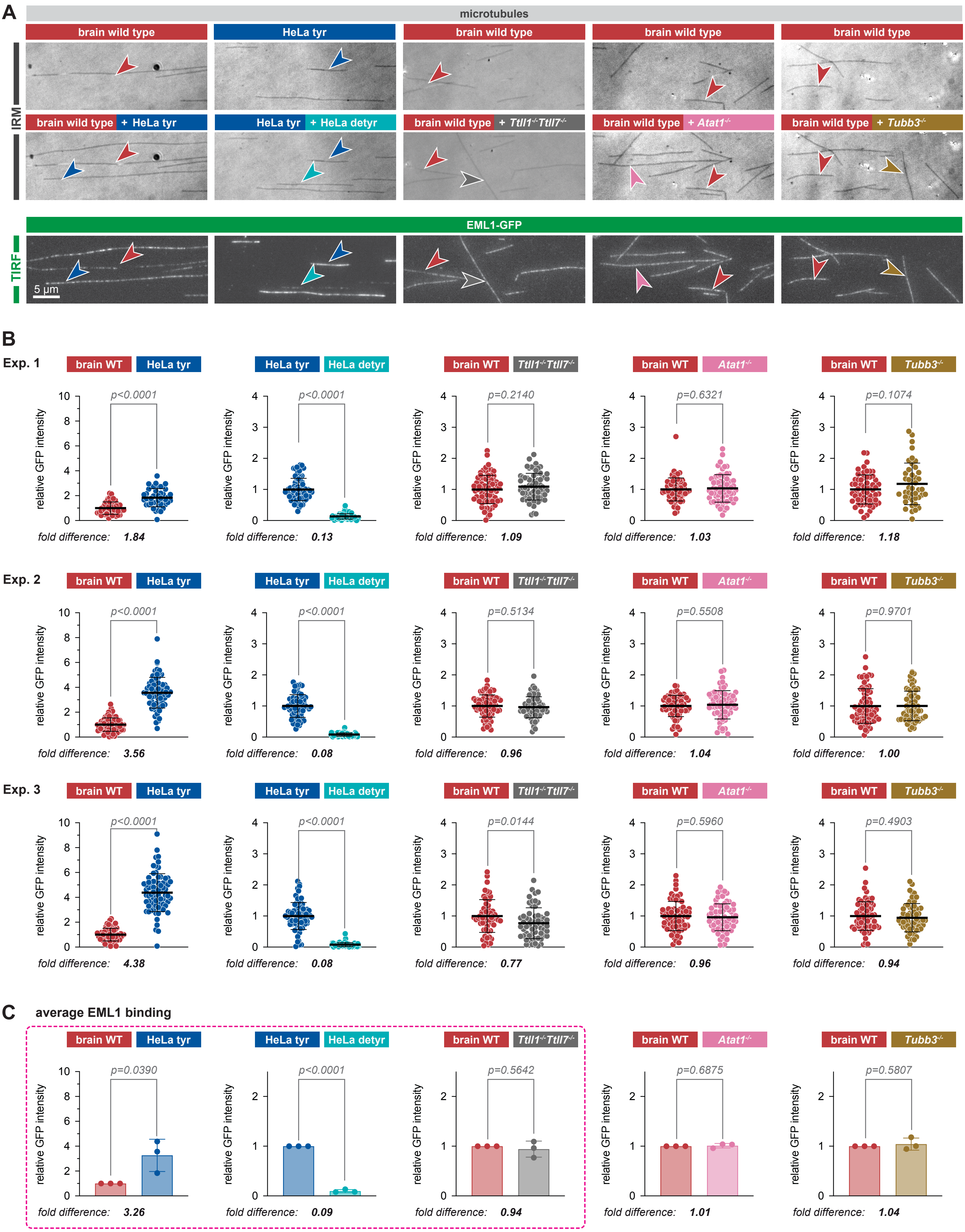

↑ Appendix Figure S7: EML1 binding to microtubules with distinct tubulin PTMs and isotype compositions.

A) TIRF microscopy-based assay showing EML1 binding from cell lysates to Taxol-stabilised microtubules polymerised from tubulin sources with different PTMs and isotype compositions. IRM images show microtubules added sequentially, with corresponding snapshots and TIRF images displaying EML1 binding.

B) Quantification of EML1-GFP intensities in three independent assays. Fluorescence values are normalised to wild-type microtubules (either wild-type brain or wild-type HeLa). Each data point represents a single microtubule. Bars represent mean ± standard deviation.

C) Average EML1 binding to different microtubule types. Each data point represents the mean value from one independent assay. Figure elements (dotted box) are also shown in Fig. 5B-D. **(B,C)** Red indicates wild-type brain microtubules; dark blue, wild-type HeLa; light blue, detyrosinated HeLa; grey, *Ttll1*$^{-/-}$*Ttll7*$^{-/-}$; pink, *Atat1*$^{-/-}$; and golden brown, *Tubb3*$^{-/-}$ mouse brain-derived microtubules. Statistical significance was determined using unpaired t-tests; p-values are shown above each comparison, and fold differences are indicated below. All experiments were performed in triplicate using independently purified tubulin and EML1 lysates prepared from three separate cell batches.

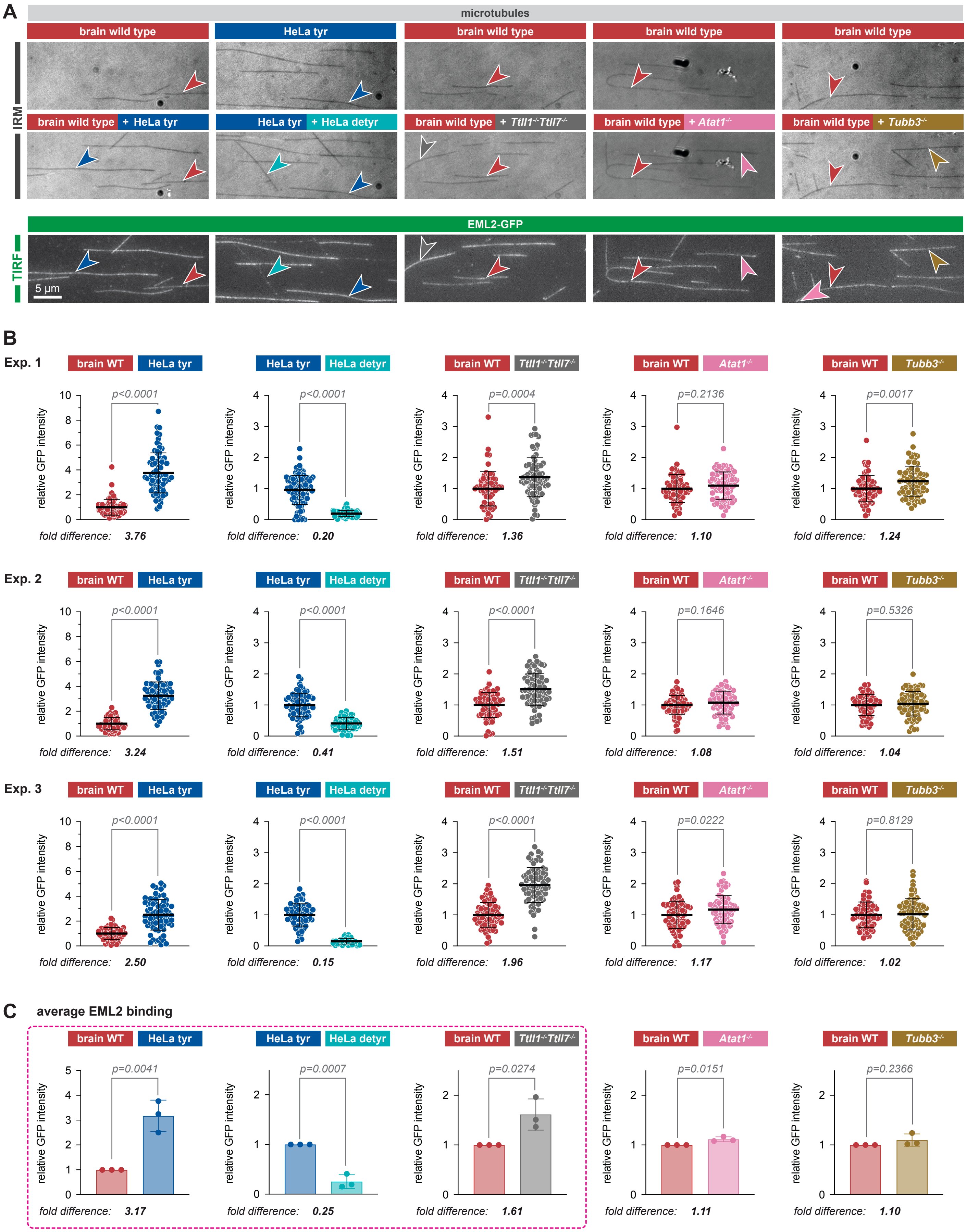
A
microtubules
brain wild type
HeLa tyr
brain wild type
brain wild type
brain wild type
IRM
brain wild type + HeLa tyr
HeLa tyr + HeLa detyr
brain wild type + Ttll1-/-Ttll7-/-
brain wild type + Atat1-/-
brain wild type + Tubb3-/-
EML2-GFP
TIRF
5 µm
B
Exp. 1
brain WT HeLa tyr
p<0.0001
fold difference: 3.76
HeLa tyr HeLa detyr
p<0.0001
fold difference: 0.20
brain WT Ttll1-/-Ttll7-/-
p=0.0004
fold difference: 1.36
brain WT Atat1-/-
p=0.2136
fold difference: 1.10
brain WT Tubb3-/-
p=0.0017
fold difference: 1.24
Exp. 2
brain WT HeLa tyr
p<0.0001
fold difference: 3.24
HeLa tyr HeLa detyr
p<0.0001
fold difference: 0.41
brain WT Ttll1-/-Ttll7-/-
p<0.0001
fold difference: 1.51
brain WT Atat1-/-
p=0.1646
fold difference: 1.08
brain WT Tubb3-/-
p=0.5326
fold difference: 1.04
Exp. 3
brain WT HeLa tyr
p<0.0001
fold difference: 2.50
HeLa tyr HeLa detyr
p<0.0001
fold difference: 0.15
brain WT Ttll1-/-Ttll7-/-
p<0.0001
fold difference: 1.96
brain WT Atat1-/-
p=0.0222
fold difference: 1.17
brain WT Tubb3-/-
p=0.8129
fold difference: 1.02
relative GFP intensity
C average EML2 binding
brain WT HeLa tyr
p=0.0041
fold difference: 3.17
HeLa tyr HeLa detyr
p=0.0007
fold difference: 0.25
brain WT Ttll1-/-Ttll7-/-
p=0.0274
fold difference: 1.61
brain WT Atat1-/-
p=0.0151
fold difference: 1.11
brain WT Tubb3-/-
p=0.2366
fold difference: 1.10

↑ Appendix Figure S8: EML2 binding to microtubules with distinct tubulin PTMs and isotype compositions.

A) TIRF microscopy-based assay showing EML2 binding from cell lysates to Taxol-stabilised microtubules polymerised from tubulin sources with different PTMs and isotype compositions. IRM images show microtubules added sequentially, with corresponding snapshots and TIRF images displaying EML2 binding.

B) Quantification of EML2-GFP intensities in three independent assays. Fluorescence values are normalised to wild-type microtubules (either wild-type brain or wild-type HeLa). Each data point represents a single microtubule. Bars represent mean ± standard deviation.

C) Average EML2 binding to different microtubule types. Each data point represents the mean value from one independent assay. Figure elements (dotted box) are also shown in Fig. 5B-D. **(B,C)** Red indicates wild-type brain microtubules; dark blue, wild-type HeLa; light blue, detyrosinated HeLa; grey, *Ttll1*$^{-/-}$*Ttll7*$^{-/-}$; pink, *Atat1*$^{-/-}$; and golden brown, *Tubb3*$^{-/-}$ mouse brain-derived microtubules. Statistical significance was determined using unpaired t-tests; p-values are shown above each comparison, and fold differences are indicated below. All experiments were performed in triplicate using independently purified tubulin and EML2 lysates prepared from three separate cell batches.

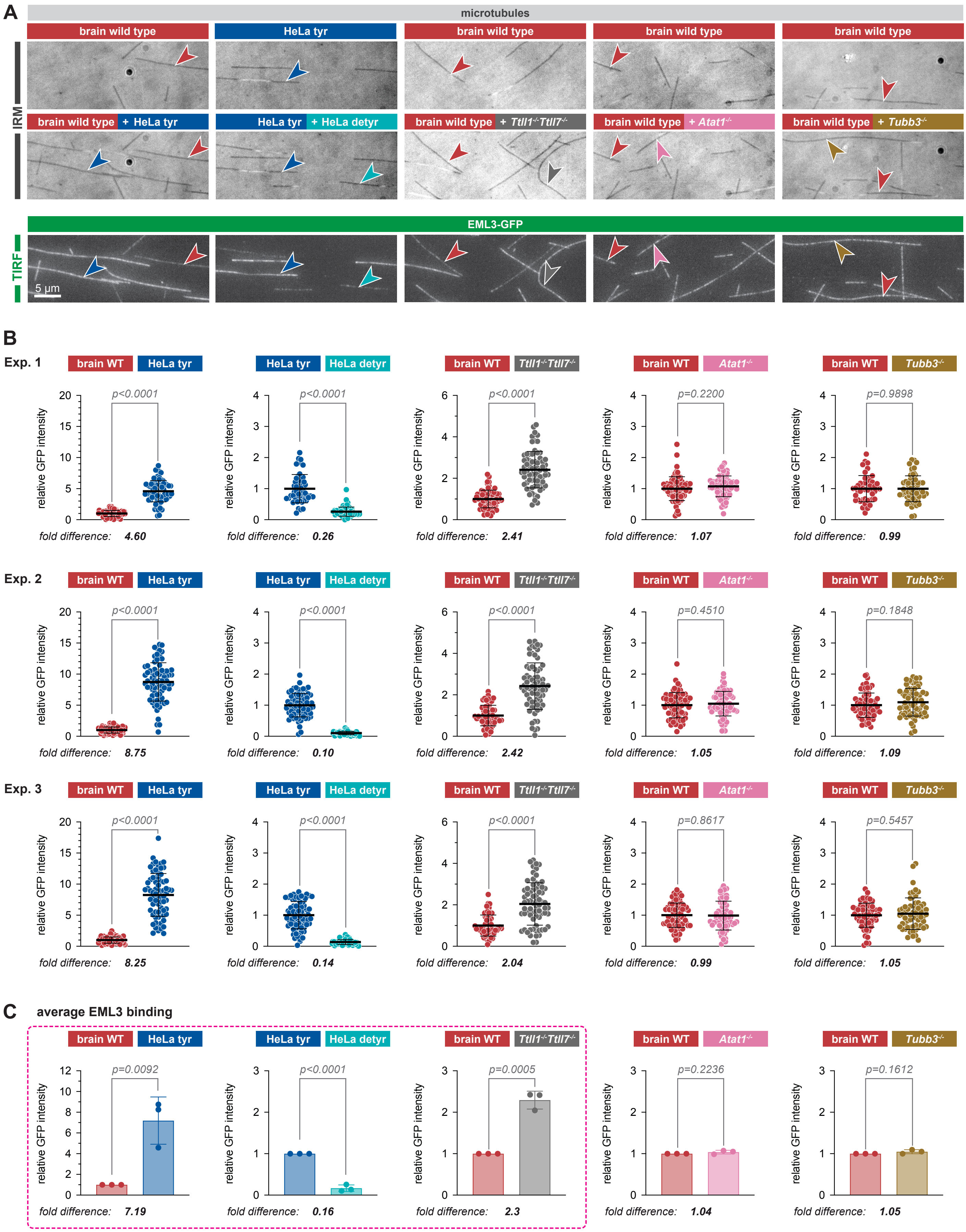
A
microtubules
brain wild type
HeLa tyr
brain wild type
brain wild type
brain wild type
IRM
brain wild type + HeLa tyr
HeLa tyr + HeLa detyr
brain wild type + Ttll1-/-Ttll7-/-
brain wild type + Atat1-/-
brain wild type + Tubb3-/-
EML3-GFP
TIRF
5 µm
B
Exp. 1
brain WT HeLa tyr
p<0.0001
fold difference: 4.60
HeLa tyr HeLa detyr
p<0.0001
fold difference: 0.26
brain WT Ttll1-/-Ttll7-/-
p<0.0001
fold difference: 2.41
brain WT Atat1-/-
p=0.2200
fold difference: 1.07
brain WT Tubb3-/-
p=0.9898
fold difference: 0.99
relative GFP intensity
Exp. 2
p<0.0001
fold difference: 8.75
p<0.0001
fold difference: 0.10
p<0.0001
fold difference: 2.42
p=0.4510
fold difference: 1.05
p=0.1848
fold difference: 1.09
Exp. 3
p<0.0001
fold difference: 8.25
p<0.0001
fold difference: 0.14
p<0.0001
fold difference: 2.04
p=0.8617
fold difference: 0.99
p=0.5457
fold difference: 1.05
C
average EML3 binding
p=0.0092
fold difference: 7.19
p<0.0001
fold difference: 0.16
p=0.0005
fold difference: 2.3
p=0.2236
fold difference: 1.04
p=0.1612
fold difference: 1.05

↑ Appendix Figure S9: EML3 binding to microtubules with distinct tubulin PTMs and isotype compositions.

A) TIRF microscopy-based assay showing EML3 binding from cell lysates to Taxol-stabilised microtubules polymerised from tubulin sources with different PTMs and isotype compositions. IRM images show microtubules added sequentially, with corresponding snapshots and TIRF images displaying EML3 binding.

B) Quantification of EML3-GFP intensities in three independent assays. Fluorescence values are normalised to wild-type microtubules (either wild-type brain or wild-type HeLa). Each data point represents a single microtubule. Bars represent mean ± standard deviation.

C) Average EML3 binding to different microtubule types. Each data point represents the mean value from one independent assay. Figure elements (dotted box) are also shown in Fig. 5B-D. **(B,C)** Red indicates wild-type brain microtubules; dark blue, wild-type HeLa; light blue, detyrosinated HeLa; grey, *Ttll1*$^{-/-}$*Ttll7*$^{-/-}$; pink, *Atat1*$^{-/-}$; and golden brown, *Tubb3*$^{-/-}$ mouse brain-derived microtubules. Statistical significance was determined using unpaired t-tests; p-values are shown above each comparison, and fold differences are indicated below. All experiments were performed in triplicate using independently purified tubulin and EML3 lysates prepared from three separate cell batches.

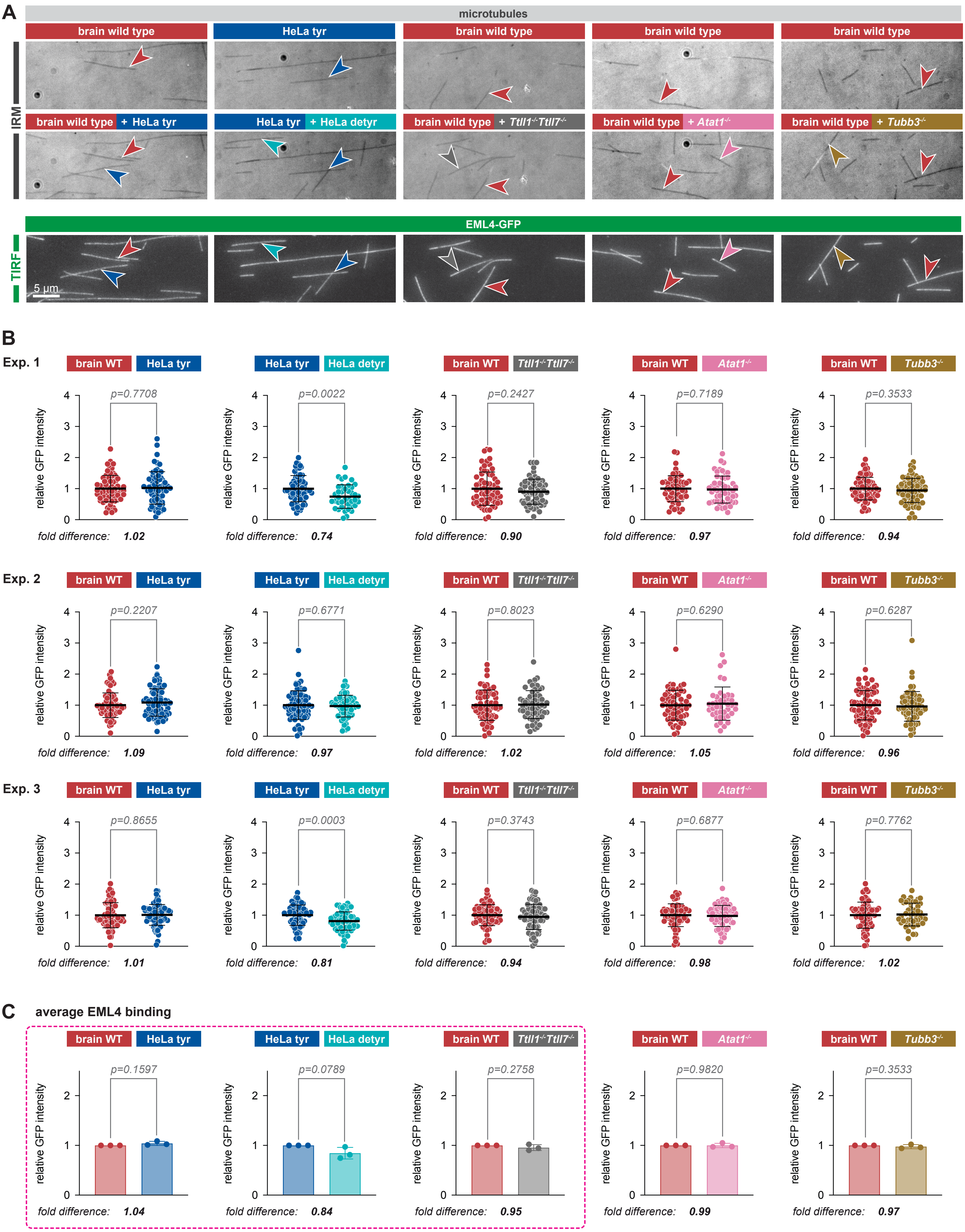
A
microtubules
brain wild type
HeLa tyr
brain wild type
brain wild type
brain wild type
IRM
brain wild type + HeLa tyr
HeLa tyr + HeLa detyr
brain wild type + Ttll1-/-Ttll7-/-
brain wild type + Atat1-/-
brain wild type + Tubb3-/-
EML4-GFP
TIRF
5 µm
B
Exp. 1
brain WT HeLa tyr p=0.7708 fold difference: 1.02
HeLa tyr HeLa detyr p=0.0022 fold difference: 0.74
brain WT Ttll1-/-Ttll7-/- p=0.2427 fold difference: 0.90
brain WT Atat1-/- p=0.7189 fold difference: 0.97
brain WT Tubb3-/- p=0.3533 fold difference: 0.94
Exp. 2
brain WT HeLa tyr p=0.2207 fold difference: 1.09
HeLa tyr HeLa detyr p=0.6771 fold difference: 0.97
brain WT Ttll1-/-Ttll7-/- p=0.8023 fold difference: 1.02
brain WT Atat1-/- p=0.6290 fold difference: 1.05
brain WT Tubb3-/- p=0.6287 fold difference: 0.96
Exp. 3
brain WT HeLa tyr p=0.8655 fold difference: 1.01
HeLa tyr HeLa detyr p=0.0003 fold difference: 0.81
brain WT Ttll1-/-Ttll7-/- p=0.3743 fold difference: 0.94
brain WT Atat1-/- p=0.6877 fold difference: 0.98
brain WT Tubb3-/- p=0.7762 fold difference: 1.02
relative GFP intensity
C average EML4 binding
brain WT HeLa tyr p=0.1597 fold difference: 1.04
HeLa tyr HeLa detyr p=0.0789 fold difference: 0.84
brain WT Ttll1-/-Ttll7-/- p=0.2758 fold difference: 0.95
brain WT Atat1-/- p=0.9820 fold difference: 0.99
brain WT Tubb3-/- p=0.3533 fold difference: 0.97

↑ Appendix Figure S10: EML4 binding to microtubules with distinct tubulin PTMs and isotype compositions.

A) TIRF microscopy-based assay showing EML4 binding from cell lysates to Taxol-stabilised microtubules polymerised from tubulin sources with different PTMs and isotype compositions. IRM images show microtubules added sequentially, with corresponding snapshots and TIRF images displaying EML4 binding.

B) Quantification of EML4-GFP intensities in three independent assays. Fluorescence values are normalised to wild-type microtubules (either wild-type brain or wild-type HeLa). Each data point represents a single microtubule. Bars represent mean ± standard deviation.

C) Average EML4 binding to different microtubule types. Each data point represents the mean value from one independent assay. Figure elements (dotted box) are also shown in Fig. 5B-D. **(B,C)** Red indicates wild-type brain microtubules; dark blue, wild-type HeLa; light blue, detyrosinated HeLa; grey, *Ttll1*$^{-/-}$*Ttll7*$^{-/-}$; pink, *Atat1*$^{-/-}$; and golden brown, *Tubb3*$^{-/-}$ mouse brain-derived microtubules. Statistical significance was determined using unpaired t-tests; p-values are shown above each comparison, and fold differences are indicated below. All experiments were performed in triplicate using independently purified tubulin and EML4 lysates prepared from three separate cell batches.

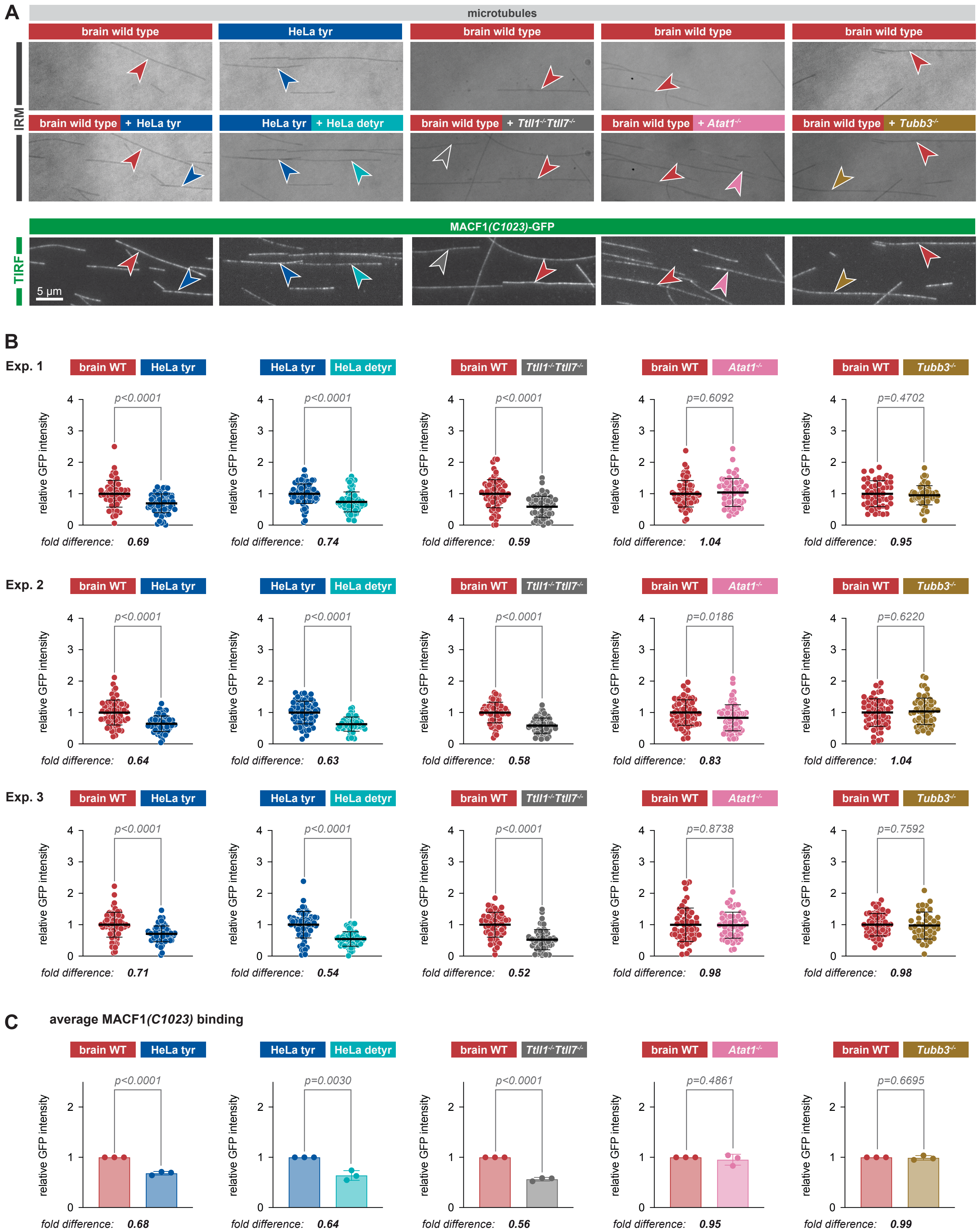
A
microtubules
brain wild type
HeLa tyr
brain wild type
brain wild type
brain wild type
IRM
brain wild type + HeLa tyr
HeLa tyr + HeLa detyr
brain wild type + Ttll1-/-Ttll7-/-
brain wild type + Atat1-/-
brain wild type + Tubb3-/-
MACF1(C1023)-GFP
TIRF
5 µm
B
Exp. 1
brain WT HeLa tyr p<0.0001 fold difference: 0.69
HeLa tyr HeLa detyr p<0.0001 fold difference: 0.74
brain WT Ttll1-/-Ttll7-/- p<0.0001 fold difference: 0.59
brain WT Atat1-/- p=0.6092 fold difference: 1.04
brain WT Tubb3-/- p=0.4702 fold difference: 0.95
Exp. 2
brain WT HeLa tyr p<0.0001 fold difference: 0.64
HeLa tyr HeLa detyr p<0.0001 fold difference: 0.63
brain WT Ttll1-/-Ttll7-/- p<0.0001 fold difference: 0.58
brain WT Atat1-/- p=0.0186 fold difference: 0.83
brain WT Tubb3-/- p=0.6220 fold difference: 1.04
Exp. 3
brain WT HeLa tyr p<0.0001 fold difference: 0.71
HeLa tyr HeLa detyr p<0.0001 fold difference: 0.54
brain WT Ttll1-/-Ttll7-/- p<0.0001 fold difference: 0.52
brain WT Atat1-/- p=0.8738 fold difference: 0.98
brain WT Tubb3-/- p=0.7592 fold difference: 0.98
relative GFP intensity
C
average MACF1(C1023) binding
brain WT HeLa tyr p<0.0001 fold difference: 0.68
HeLa tyr HeLa detyr p=0.0030 fold difference: 0.64
brain WT Ttll1-/-Ttll7-/- p<0.0001 fold difference: 0.56
brain WT Atat1-/- p=0.4861 fold difference: 0.95
brain WT Tubb3-/- p=0.6695 fold difference: 0.99
relative GFP intensity

↑ Appendix Figure S11: MACF1*(C1023)* binding to microtubules with distinct tubulin PTMs and isotype compositions.

A) TIRF microscopy-based assay showing MACF1*(C1023)* binding from cell lysates to Taxol-stabilised microtubules polymerised from tubulin sources with different PTMs and isotype compositions. IRM images show microtubules added sequentially, with corresponding snapshots and TIRF images displaying MACF1*(C1023)* binding.

B) Quantification of MACF1*(C1023)*-GFP intensities in three independent assays. Fluorescence values are normalised to wild-type microtubules (either wild-type brain or wild-type HeLa). Each data point represents a single microtubule. Bars represent mean ± standard deviation.

C) Average MACF1*(C1023)* binding to different microtubule types. Each data point represents the mean value from one independent assay. **(B,C)** Red indicates wild-type brain microtubules; dark blue, wild-type HeLa; light blue, detyrosinated HeLa; grey, $Ttll1^{-/-}Ttll7^{-/-}$; pink, $Atat1^{-/-}$; and golden brown, $Tubb3^{-/-}$ mouse brain-derived microtubules. Statistical significance was determined using unpaired t-tests; p-values are shown above each comparison, and fold differences are indicated below. All experiments were performed in triplicate using independently purified tubulin and MACF1*(C1023)* lysates prepared from three separate cell batches.

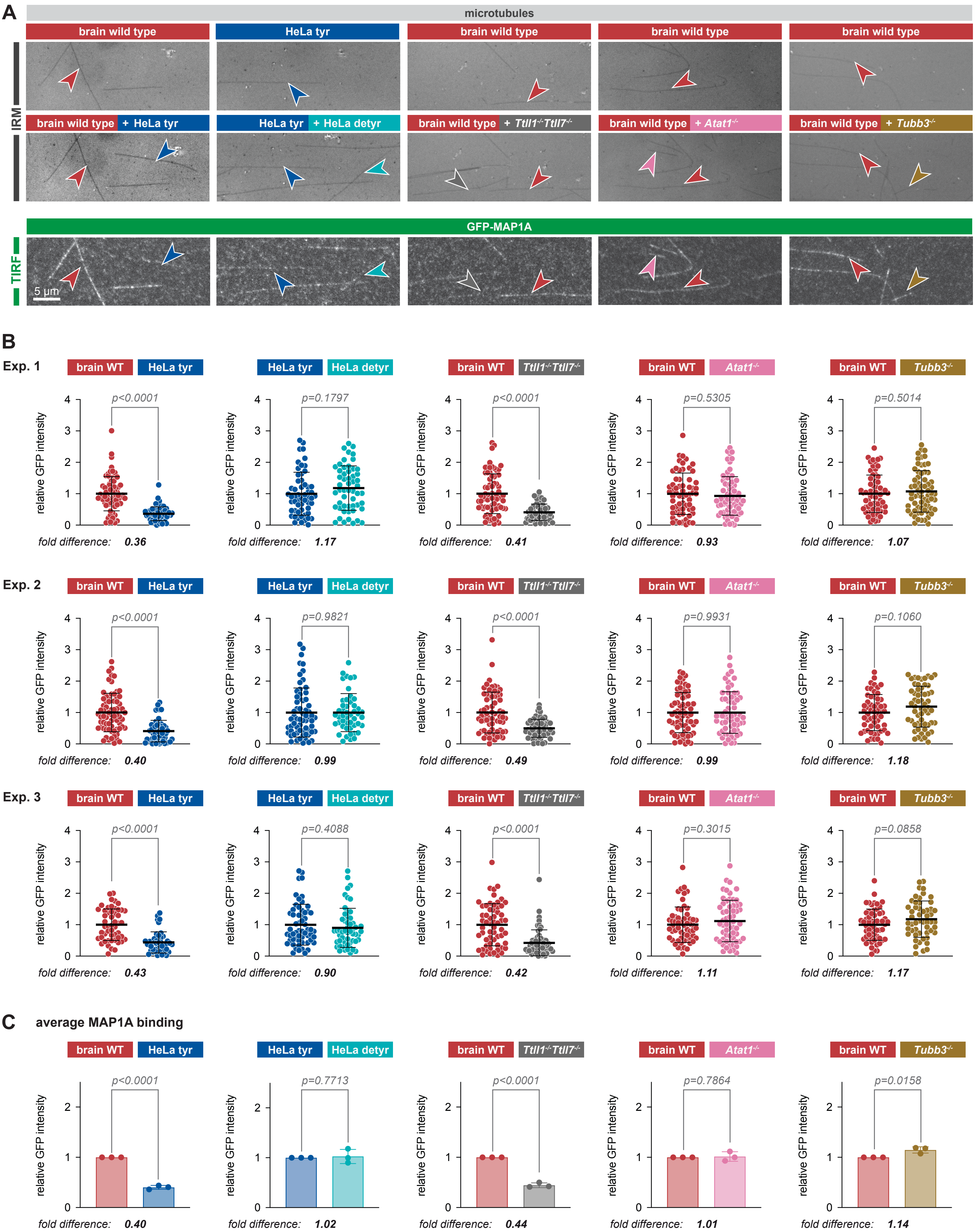
A
microtubules
brain wild type
HeLa tyr
brain wild type
brain wild type
brain wild type
IRM
brain wild type + HeLa tyr
HeLa tyr + HeLa detyr
brain wild type + Ttll1-/-Ttll7-/-
brain wild type + Atat1-/-
brain wild type + Tubb3-/-
GFP-MAP1A
TIRF
5 µm
B
Exp. 1
brain WT HeLa tyr
p<0.0001
fold difference: 0.36
HeLa tyr HeLa detyr
p=0.1797
fold difference: 1.17
brain WT Ttll1-/-Ttll7-/-
p<0.0001
fold difference: 0.41
brain WT Atat1-/-
p=0.5305
fold difference: 0.93
brain WT Tubb3-/-
p=0.5014
fold difference: 1.07
relative GFP intensity
Exp. 2
p<0.0001
fold difference: 0.40
p=0.9821
fold difference: 0.99
p<0.0001
fold difference: 0.49
p=0.9931
fold difference: 0.99
p=0.1060
fold difference: 1.18
Exp. 3
p<0.0001
fold difference: 0.43
p=0.4088
fold difference: 0.90
p<0.0001
fold difference: 0.42
p=0.3015
fold difference: 1.11
p=0.0858
fold difference: 1.17
C
average MAP1A binding
brain WT HeLa tyr
p<0.0001
fold difference: 0.40
HeLa tyr HeLa detyr
p=0.7713
fold difference: 1.02
brain WT Ttll1-/-Ttll7-/-
p<0.0001
fold difference: 0.44
brain WT Atat1-/-
p=0.7864
fold difference: 1.01
brain WT Tubb3-/-
p=0.0158
fold difference: 1.14

↑ Appendix Figure S12: MAP1A binding to microtubules with distinct tubulin PTMs and isotype compositions.

A) TIRF microscopy-based assay showing MAP1A binding from cell lysates to Taxol-stabilised microtubules polymerised from tubulin sources with different PTMs and isotype compositions. IRM images show microtubules added sequentially, with corresponding snapshots and TIRF images displaying MAP1A binding.

B) Quantification of GFP-MAP1A intensities in three independent assays. Fluorescence values are normalised to wild-type microtubules (either wild-type brain or wild-type HeLa). Each data point represents a single microtubule. Bars represent mean ± standard deviation.

C) Average MAP1A binding to different microtubule types. Each data point represents the mean value from one independent assay. **(B,C)** Red indicates wild-type brain microtubules; dark blue, wild-type HeLa; light blue, detyrosinated HeLa; grey, *Ttll1*$^{-/-}$*Ttll7*$^{-/-}$; pink, *Atat1*$^{-/-}$; and golden brown, *Tubb3*$^{-/-}$ mouse brain-derived microtubules. Statistical significance was determined using unpaired t-tests; p-values are shown above each comparison, and fold differences are indicated below. All experiments were performed in triplicate using independently purified tubulin and MAP1A lysates prepared from three separate cell batches.

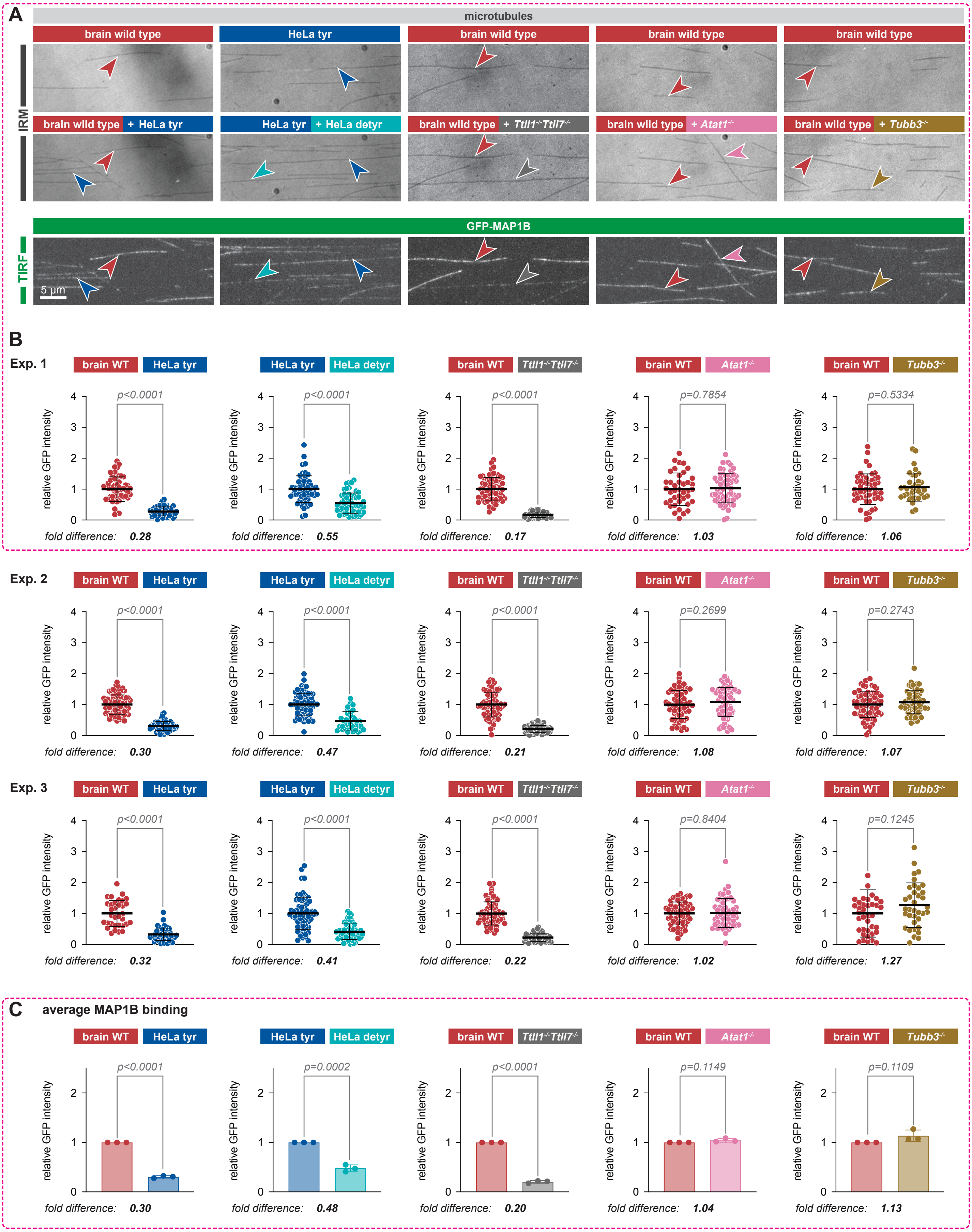
A
microtubules
brain wild type
HeLa tyr
IRM
brain wild type + HeLa tyr
HeLa tyr + HeLa detyr
brain wild type + Ttll1-/-Ttll7-/-
brain wild type + Atat1-/-
brain wild type + Tubb3-/-
GFP-MAP1B
TIRF
5 µm
B
Exp. 1
brain WT
HeLa tyr
HeLa detyr
Ttll1-/-Ttll7-/-
Atat1-/-
Tubb3-/-
relative GFP intensity
p<0.0001
p=0.7854
p=0.5334
fold difference: 0.28
fold difference: 0.55
fold difference: 0.17
fold difference: 1.03
fold difference: 1.06
Exp. 2
p=0.2699
p=0.2743
fold difference: 0.30
fold difference: 0.47
fold difference: 0.21
fold difference: 1.08
fold difference: 1.07
Exp. 3
p=0.8404
p=0.1245
fold difference: 0.32
fold difference: 0.41
fold difference: 0.22
fold difference: 1.02
fold difference: 1.27
C
average MAP1B binding
p=0.0002
p=0.1149
p=0.1109
fold difference: 0.30
fold difference: 0.48
fold difference: 0.20
fold difference: 1.04
fold difference: 1.13

↑ Appendix Figure S13: MAP1B binding to microtubules with distinct tubulin PTMs and isotype compositions.

A) TIRF microscopy-based assay showing MAP1B binding from cell lysates to Taxol-stabilised microtubules polymerised from tubulin sources with different PTMs and isotype compositions. IRM images show microtubules added sequentially, with corresponding snapshots and TIRF images displaying MAP1B binding.

B) Quantification of GFP-MAP1B intensities in three independent assays. Fluorescence values are normalised to wild-type microtubules (either wild-type brain or wild-type HeLa). Each data point represents a single microtubule. Bars represent mean ± standard deviation.

C) Average MAP1B binding to different microtubule types. Each data point represents the mean value from one independent assay. **(A,B,C)** Figure elements (dotted boxes) are also shown in Fig. 3A-C). **(B,C)** Red indicates wild-type brain microtubules; dark blue, wild-type HeLa; light blue, detyrosinated HeLa; grey, *Ttll1*$^{-/-}$*Ttll7*$^{-/-}$; pink, *Atat1*$^{-/-}$; and golden brown, *Tubb3*$^{-/-}$ mouse brain-derived microtubules. Statistical significance was determined using unpaired t-tests; p-values are shown above each comparison, and fold differences are indicated below. All experiments were performed in triplicate using independently purified tubulin and MAP1B lysates prepared from three separate cell batches.

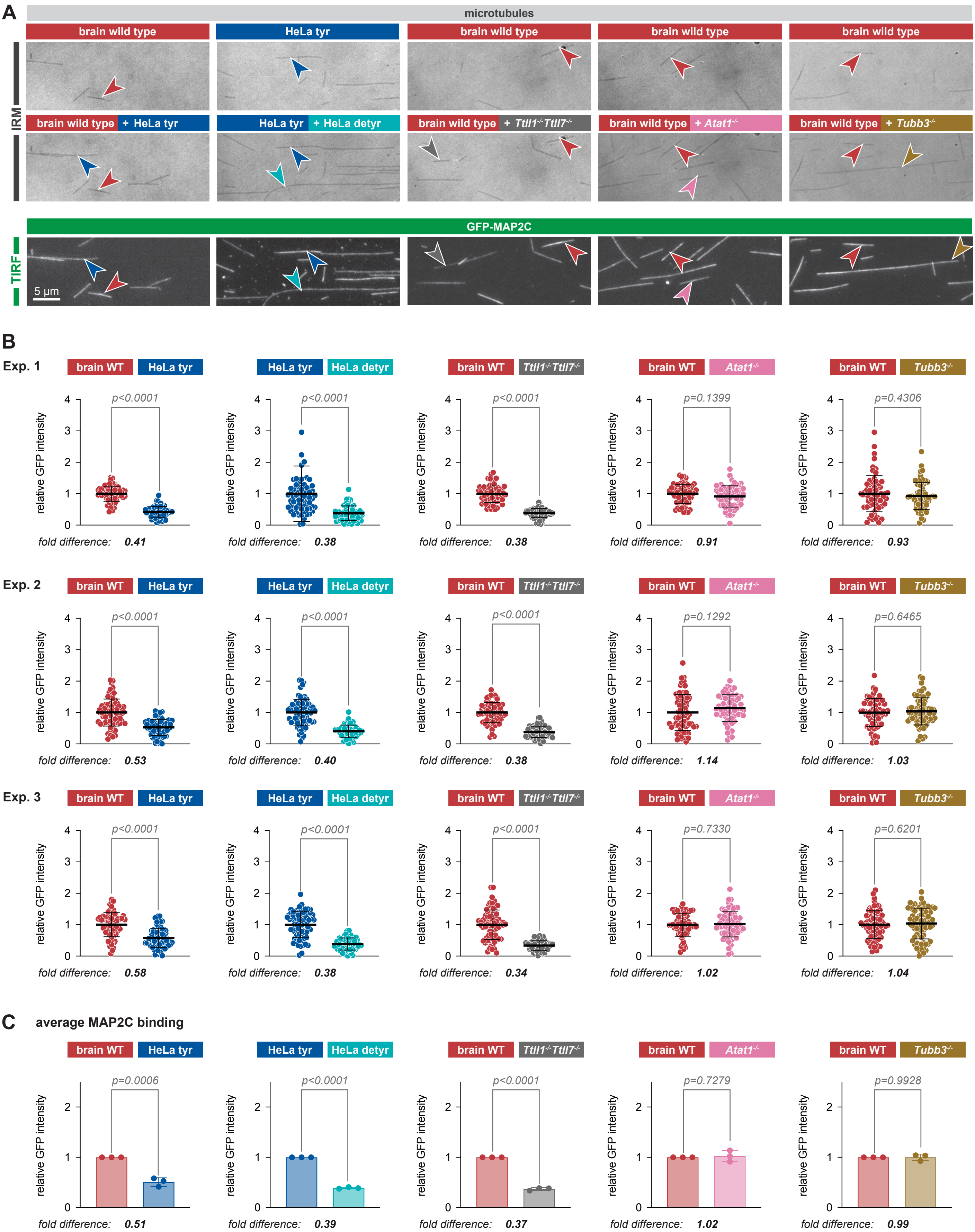
A
microtubules
brain wild type
HeLa tyr
IRM
brain wild type + HeLa tyr
HeLa tyr + HeLa detyr
brain wild type + Ttll1-/-Ttll7-/-
brain wild type + Atat1-/-
brain wild type + Tubb3-/-
GFP-MAP2C
TIRF
5 µm
B
Exp. 1
brain WT
HeLa tyr
HeLa detyr
Ttll1-/-Ttll7-/-
Atat1-/-
Tubb3-/-
relative GFP intensity
p<0.0001
p=0.1399
p=0.4306
fold difference: 0.41
fold difference: 0.38
fold difference: 0.38
fold difference: 0.91
fold difference: 0.93
Exp. 2
p<0.0001
p=0.1292
p=0.6465
fold difference: 0.53
fold difference: 0.40
fold difference: 0.38
fold difference: 1.14
fold difference: 1.03
Exp. 3
p<0.0001
p=0.7330
p=0.6201
fold difference: 0.58
fold difference: 0.38
fold difference: 0.34
fold difference: 1.02
fold difference: 1.04
C
average MAP2C binding
p=0.0006
p<0.0001
p=0.7279
p=0.9928
fold difference: 0.51
fold difference: 0.39
fold difference: 0.37
fold difference: 1.02
fold difference: 0.99

↑ Appendix Figure S14: MAP2C binding to microtubules with distinct tubulin PTMs and isotype compositions.

A) TIRF microscopy-based assay showing MAP2C binding from cell lysates to GMPCPP-stabilised microtubules polymerised from tubulin sources with different PTMs and isotype compositions. IRM images show microtubules added sequentially, with corresponding snapshots and TIRF images displaying MAP2C binding.

B) Quantification of GFP-MAP2C intensities in three independent assays. Fluorescence values are normalised to wild-type microtubules (either wild-type brain or wild-type HeLa). Each data point represents a single microtubule. Bars represent mean ± standard deviation.

C) Average MAP2C binding to different microtubule types. Each data point represents the mean value from one independent assay. **(B,C)** Red indicates wild-type brain microtubules; dark blue, wild-type HeLa; light blue, detyrosinated HeLa; grey, *Ttll1*$^{-/-}$*Ttll7*$^{-/-}$; pink, *Atat1*$^{-/-}$ and golden brown, *Tubb3*$^{-/-}$ mouse brain-derived microtubules. Statistical significance was determined using unpaired t-tests; p-values are shown above each comparison, and fold differences are indicated below. All experiments were performed in triplicate using independently purified tubulin and MAP2C lysates prepared from three separate cell batches.

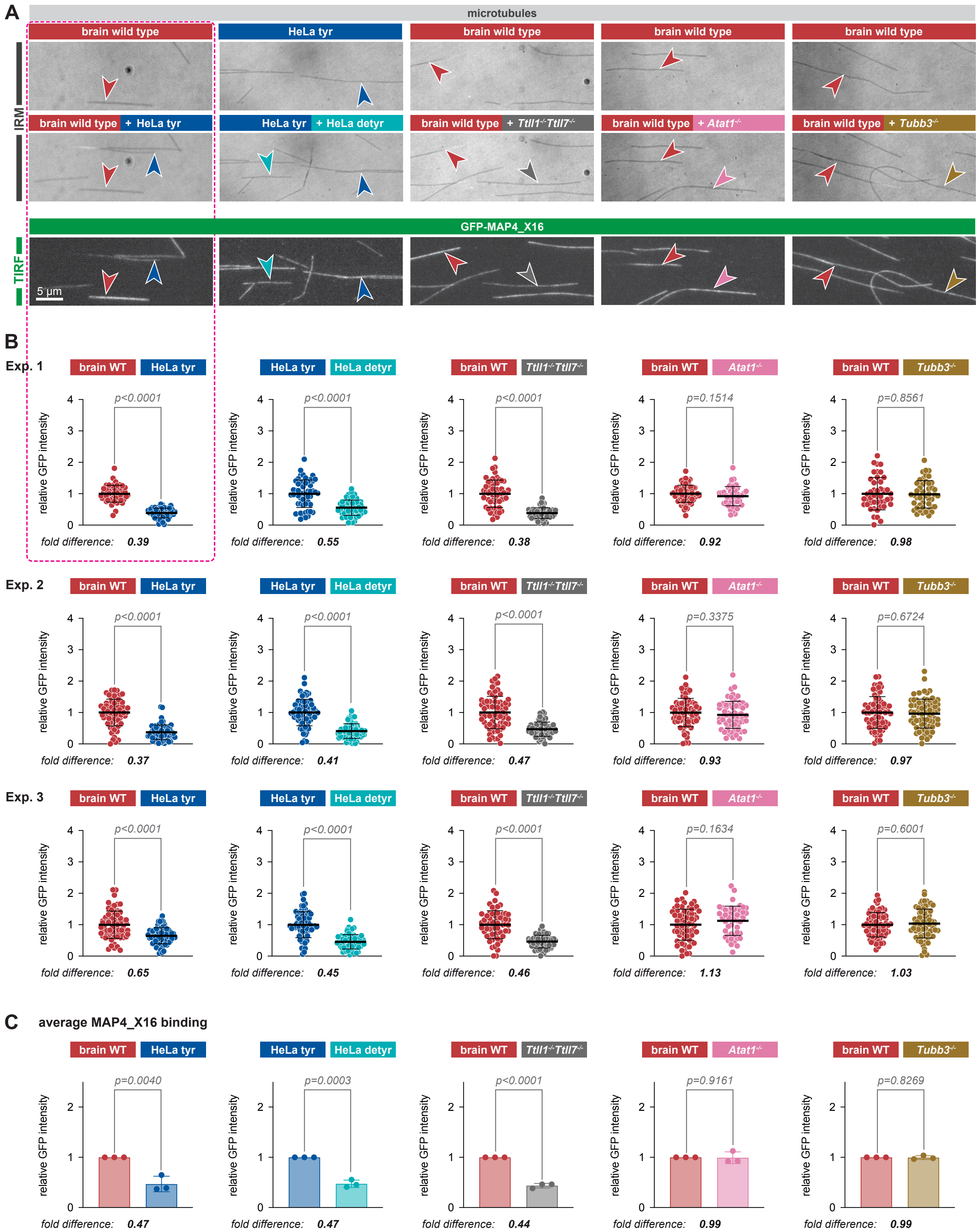
A
microtubules
brain wild type
HeLa tyr
brain wild type
brain wild type
brain wild type
IRM
brain wild type + HeLa tyr
HeLa tyr + HeLa detyr
brain wild type + Ttll1-/-Ttll7-/-
brain wild type + Atat1-/-
brain wild type + Tubb3-/-
GFP-MAP4_X16
TIRF
5 µm
B
Exp. 1
brain WT HeLa tyr
HeLa tyr HeLa detyr
brain WT Ttll1-/-Ttll7-/-
brain WT Atat1-/-
brain WT Tubb3-/-
relative GFP intensity
p<0.0001
p<0.0001
p<0.0001
p=0.1514
p=0.8561
fold difference: 0.39
fold difference: 0.55
fold difference: 0.38
fold difference: 0.92
fold difference: 0.98
Exp. 2
p<0.0001
p<0.0001
p<0.0001
p=0.3375
p=0.6724
fold difference: 0.37
fold difference: 0.41
fold difference: 0.47
fold difference: 0.93
fold difference: 0.97
Exp. 3
p<0.0001
p<0.0001
p<0.0001
p=0.1634
p=0.6001
fold difference: 0.65
fold difference: 0.45
fold difference: 0.46
fold difference: 1.13
fold difference: 1.03
C
average MAP4_X16 binding
p=0.0040
p=0.0003
p<0.0001
p=0.9161
p=0.8269
fold difference: 0.47
fold difference: 0.47
fold difference: 0.44
fold difference: 0.99
fold difference: 0.99

↑ Appendix Figure S15: MAP4_X16 binding to microtubules with distinct tubulin PTMs and isotype compositions.

A) TIRF microscopy-based assay showing MAP4_X16 binding from cell lysates to Taxol-stabilised microtubules polymerised from tubulin sources with different PTMs and isotype compositions. IRM images show microtubules added sequentially, with corresponding snapshots and TIRF images displaying MAP4_X16 binding.

B) Quantification of GFP-MAP4_X16 intensities in three independent assays. Fluorescence values are normalised to wild-type microtubules (either wild-type brain or wild-type HeLa). Each data point represents a single microtubule. Bars represent mean ± standard deviation.

C) Average MAP4_X16 binding to different microtubule types. Each data point represents the mean value from one independent assay. **(A,B)** Figure elements in dotted box are also shown in Fig. 2B,C. **(B,C)** Red indicates wild-type brain microtubules; dark blue, wild-type HeLa; light blue, detyrosinated HeLa; grey, *Ttll1*$^{-/-}$*Ttll7*$^{-/-}$; pink, *Atat1*$^{-/-}$ and golden brown, *Tubb3*$^{-/-}$ mouse brain-derived microtubules. Statistical significance was determined using unpaired t-tests; p-values are shown above each comparison, and fold differences are indicated below. All experiments were performed in triplicate using independently purified tubulin and MAP4_X16 lysates prepared from three separate cell batches.

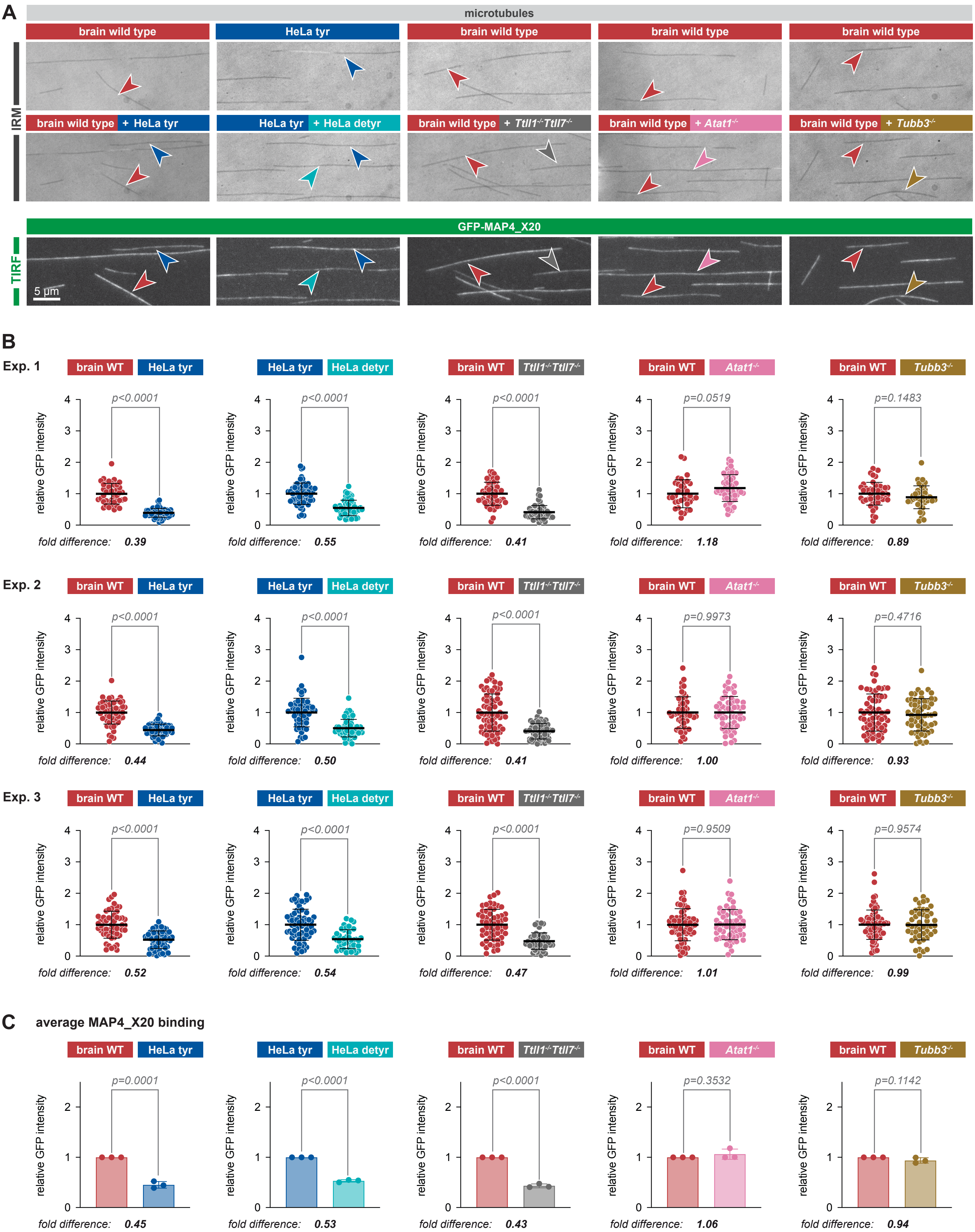
A
microtubules
brain wild type
HeLa tyr
brain wild type + HeLa tyr
HeLa tyr + HeLa detyr
brain wild type + Ttll1-/-Ttll7-/-
brain wild type + Atat1-/-
brain wild type + Tubb3-/-
IRM
GFP-MAP4_X20
TIRF
5 µm
B
Exp. 1
brain WT
HeLa tyr
HeLa detyr
Ttll1-/-Ttll7-/-
Atat1-/-
Tubb3-/-
relative GFP intensity
p<0.0001
p=0.0519
p=0.1483
fold difference: 0.39
fold difference: 0.55
fold difference: 0.41
fold difference: 1.18
fold difference: 0.89
Exp. 2
p=0.9973
p=0.4716
fold difference: 0.44
fold difference: 0.50
fold difference: 0.41
fold difference: 1.00
fold difference: 0.93
Exp. 3
p=0.9509
p=0.9574
fold difference: 0.52
fold difference: 0.54
fold difference: 0.47
fold difference: 1.01
fold difference: 0.99
C
average MAP4_X20 binding
p=0.0001
p<0.0001
p=0.3532
p=0.1142
fold difference: 0.45
fold difference: 0.53
fold difference: 0.43
fold difference: 1.06
fold difference: 0.94

↑ Appendix Figure S16: MAP4_X20 binding to microtubules with distinct tubulin PTMs and isotype compositions.

A) TIRF microscopy-based assay showing MAP4_X20 binding from cell lysates to Taxol-stabilised microtubules polymerised from tubulin sources with different PTMs and isotype compositions. IRM images show microtubules added sequentially, with corresponding snapshots and TIRF images displaying MAP4_X20 binding.

B) Quantification of GFP-MAP4_X20 intensities in three independent assays. Fluorescence values are normalised to wild-type microtubules (either wild-type brain or wild-type HeLa). Each data point represents a single microtubule. Bars represent mean ± standard deviation.

C) Average MAP4_X20 binding to different microtubule types. Each data point represents the mean value from one independent assay. **(B,C)** Red indicates wild-type brain microtubules; dark blue, wild-type HeLa; light blue, detyrosinated HeLa; grey, grey, *Ttll1*$^{-/-}$*Ttll7*$^{-/-}$; pink, *Atat1*$^{-/-}$; and golden brown, *Tubb3*$^{-/-}$ mouse brain-derived microtubules. Statistical significance was determined using unpaired t-tests; p-values are shown above each comparison, and fold differences are indicated below. All experiments were performed in triplicate using independently purified tubulin and MAP4_X20 lysates prepared from three separate cell batches.

Krishnan et al. Appendix Figure S17

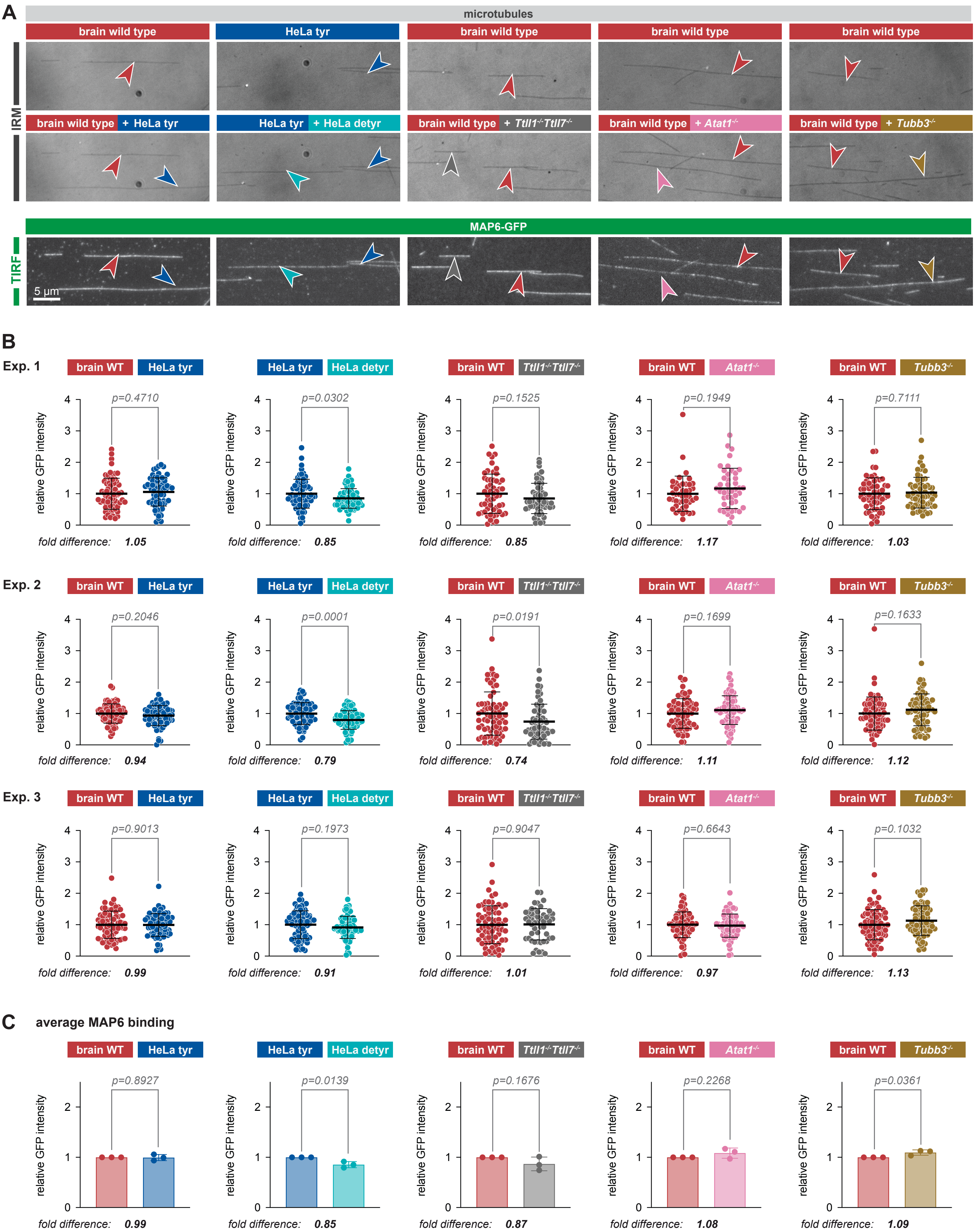

↑ Appendix Figure S17: MAP6 binding to microtubules with distinct tubulin PTMs and isotype compositions.

A) TIRF microscopy-based assay showing MAP6 binding from cell lysates to Taxol-stabilised microtubules polymerised from tubulin sources with different PTMs and isotype compositions. IRM images show microtubules added sequentially, with corresponding snapshots and TIRF images displaying MAP6 binding.

B) Quantification of MAP6-GFP intensities in three independent assays. Fluorescence values are normalised to wild-type microtubules (either wild-type brain or wild-type HeLa). Each data point represents a single microtubule. Bars represent mean ± standard deviation.

C) Average MAP6 binding to different microtubule types. Each data point represents the mean value from one independent assay. **(B,C)** Red indicates wild-type brain microtubules; dark blue, wild-type HeLa; light blue, detyrosinated HeLa; grey, *Ttll1*$^{-/-}$*Ttll7*$^{-/-}$; pink, *Atat1*$^{-/-}$ and golden brown, *Tubb3*$^{-/-}$ mouse brain-derived microtubules. Statistical significance was determined using unpaired t-tests; p-values are shown above each comparison, and fold differences are indicated below. All experiments were performed in triplicate using independently purified tubulin and MAP6 lysates prepared from three separate cell batches.

Krishnan et al. Appendix Figure S18

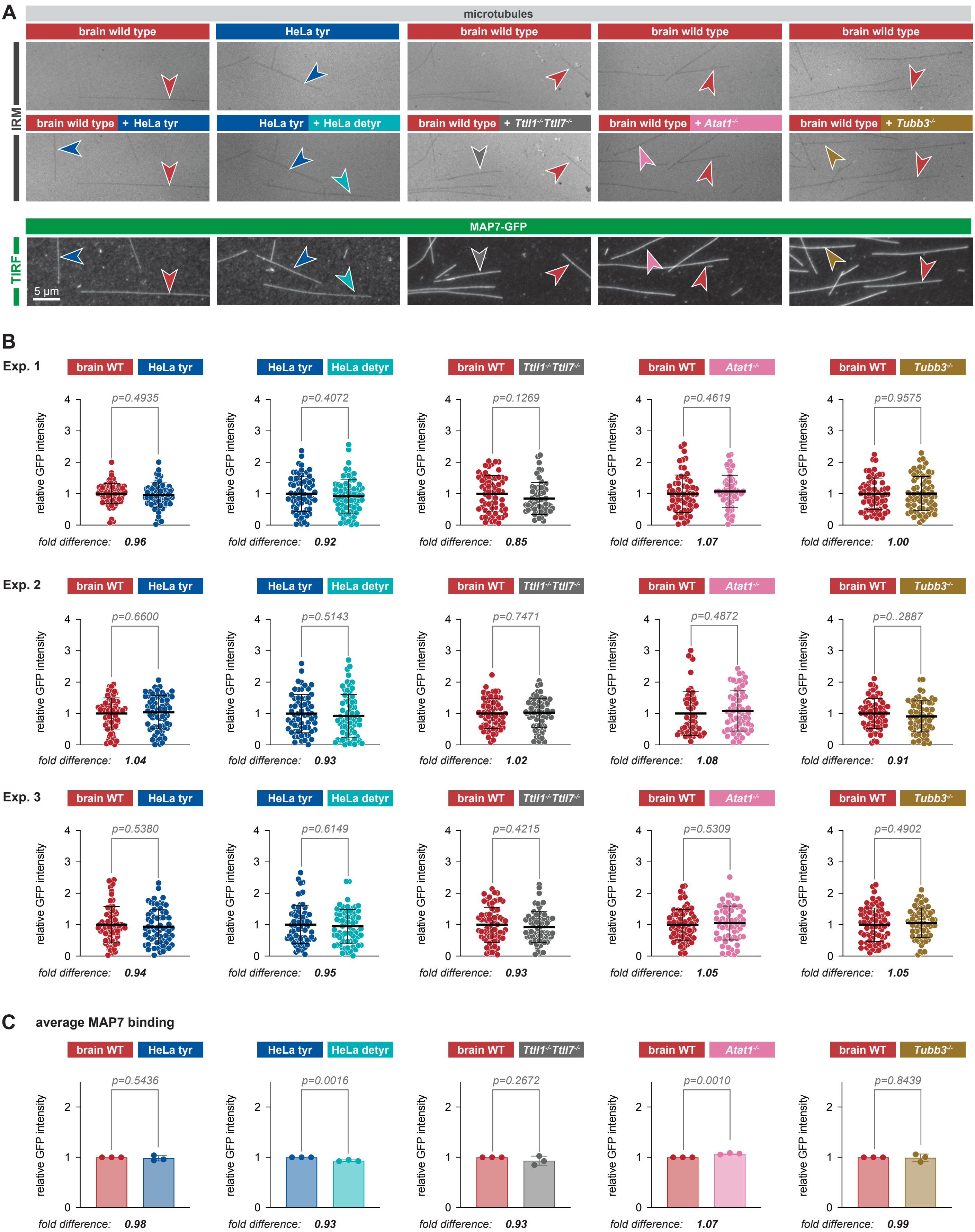

↑ Appendix Figure S18: MAP7 binding to microtubules with distinct tubulin PTMs and isotype compositions.

A) TIRF microscopy-based assay showing MAP7 binding from cell lysates to Taxol-stabilised microtubules polymerised from tubulin sources with different PTMs and isotype compositions. IRM images show microtubules added sequentially, with corresponding snapshots and TIRF images displaying MAP7 binding.

B) Quantification of MAP7-GFP intensities in three independent assays. Fluorescence values are normalised to wild-type microtubules (either wild-type brain or wild-type HeLa). Each data point represents a single microtubule. Bars represent mean ± standard deviation.

C) Average MAP7 binding to different microtubule types. Each data point represents the mean value from one independent assay. **(B,C)** Red indicates wild-type brain microtubules; dark blue, wild-type HeLa; light blue, detyrosinated HeLa; grey, *Ttll1*$^{-/-}$*Ttll7*$^{-/-}$; pink, *Atat1*$^{-/-}$ and golden brown, *Tubb3*$^{-/-}$ mouse brain-derived microtubules. Statistical significance was determined using unpaired t-tests; p-values are shown above each comparison, and fold differences are indicated below. All experiments were performed in triplicate using independently purified tubulin and MAP7 lysates prepared from three separate cell batches.

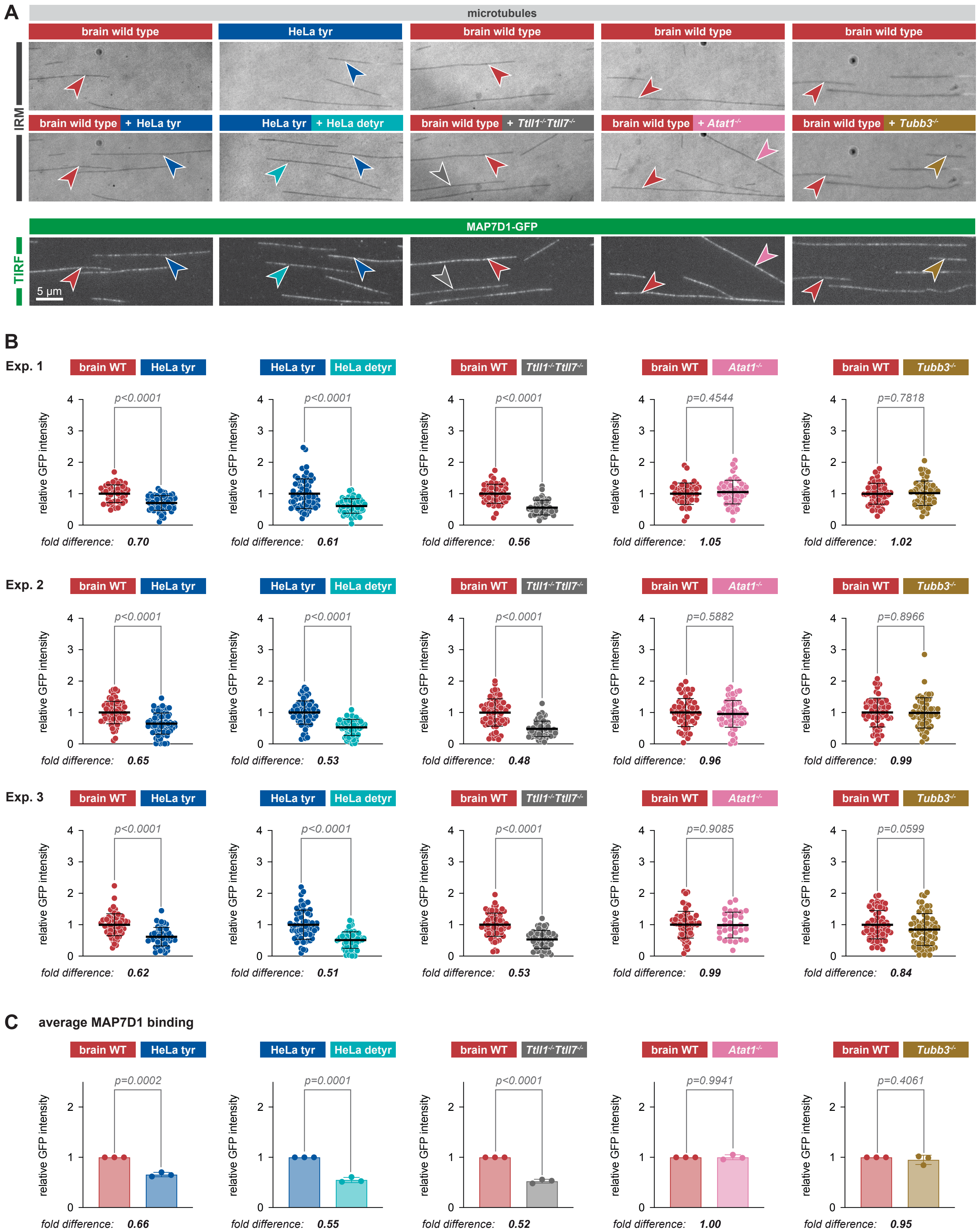
A
microtubules
brain wild type
HeLa tyr
brain wild type
brain wild type
brain wild type
IRM
brain wild type + HeLa tyr
HeLa tyr + HeLa detyr
brain wild type + Ttll1-/-Ttll7-/-
brain wild type + Atat1-/-
brain wild type + Tubb3-/-
MAP7D1-GFP
TIRF
5 µm
B
Exp. 1
brain WT HeLa tyr
HeLa tyr HeLa detyr
brain WT Ttll1-/-Ttll7-/-
brain WT Atat1-/-
brain WT Tubb3-/-
relative GFP intensity
p<0.0001
p<0.0001
p<0.0001
p=0.4544
p=0.7818
fold difference: 0.70
fold difference: 0.61
fold difference: 0.56
fold difference: 1.05
fold difference: 1.02
Exp. 2
p<0.0001
p<0.0001
p<0.0001
p=0.5882
p=0.8966
fold difference: 0.65
fold difference: 0.53
fold difference: 0.48
fold difference: 0.96
fold difference: 0.99
Exp. 3
p<0.0001
p<0.0001
p<0.0001
p=0.9085
p=0.0599
fold difference: 0.62
fold difference: 0.51
fold difference: 0.53
fold difference: 0.99
fold difference: 0.84
C
average MAP7D1 binding
p=0.0002
p=0.0001
p<0.0001
p=0.9941
p=0.4061
fold difference: 0.66
fold difference: 0.55
fold difference: 0.52
fold difference: 1.00
fold difference: 0.95

↑ Appendix Figure S19: MAP7D1 binding to microtubules with distinct tubulin PTMs and isotype compositions.

A) TIRF microscopy-based assay showing MAP7D1 binding from cell lysates to Taxol-stabilised microtubules polymerised from tubulin sources with different PTMs and isotype compositions. IRM images show microtubules added sequentially, with corresponding snapshots and TIRF images displaying MAP7D1 binding.

B) Quantification of MAP7D1-GFP intensities in three independent assays. Fluorescence values are normalised to wild-type microtubules (either wild-type brain or wild-type HeLa). Each data point represents a single microtubule. Bars represent mean ± standard deviation.

C) Average MAP7D1 binding to different microtubule types. Each data point represents the mean value from one independent assay. **(B,C)** Red indicates wild-type brain microtubules; dark blue, wild-type HeLa; light blue, detyrosinated HeLa; grey, *Ttll1*$^{-/-}$*Ttll7*$^{-/-}$; pink, *Atat1*$^{-/-}$ and golden brown, *Tubb3*$^{-/-}$ mouse brain-derived microtubules. Statistical significance was determined using unpaired t-tests; p-values are shown above each comparison, and fold differences are indicated below. All experiments were performed in triplicate using independently purified tubulin and MAP7D1 lysates prepared from three separate cell batches.

Krishnan et al. Appendix Figure S20

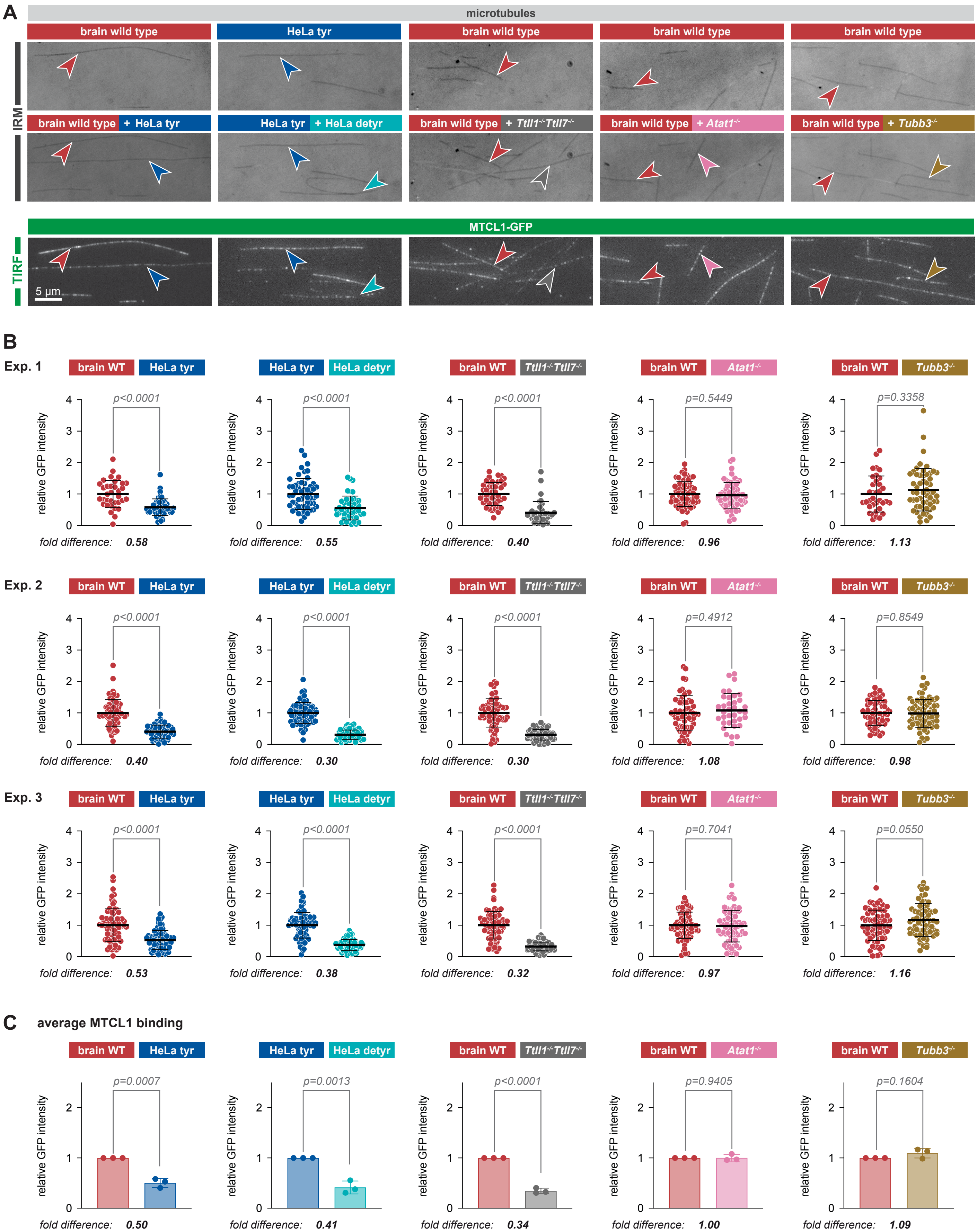

↑ Appendix Figure S20: MTCL1 binding to microtubules with distinct tubulin PTMs and isotype compositions.

A) TIRF microscopy-based assay showing MTCL1 binding from cell lysates to Taxol-stabilised microtubules polymerised from tubulin sources with different PTMs and isotype compositions. IRM images show microtubules added sequentially, with corresponding snapshots and TIRF images displaying MTCL1 binding.

B) Quantification of MTCL1-GFP intensities in three independent assays. Fluorescence values are normalised to wild-type microtubules (either wild-type brain or wild-type HeLa). Each data point represents a single microtubule. Bars represent mean ± standard deviation.

C) Average MTCL1 binding to different microtubule types. Each data point represents the mean value from one independent assay. **(B,C)** Red indicates wild-type brain microtubules; dark blue, wild-type HeLa; light blue, detyrosinated HeLa; grey, *Ttll1*$^{-/-}$*Ttll7*$^{-/-}$; pink, *Atat1*$^{-/-}$; and golden brown, *Tubb3*$^{-/-}$ mouse brain-derived microtubules. Statistical significance was determined using unpaired t-tests; p-values are shown above each comparison, and fold differences are indicated below. All experiments were performed in triplicate using independently purified tubulin and MTCL1 lysates prepared from three separate cell batches.

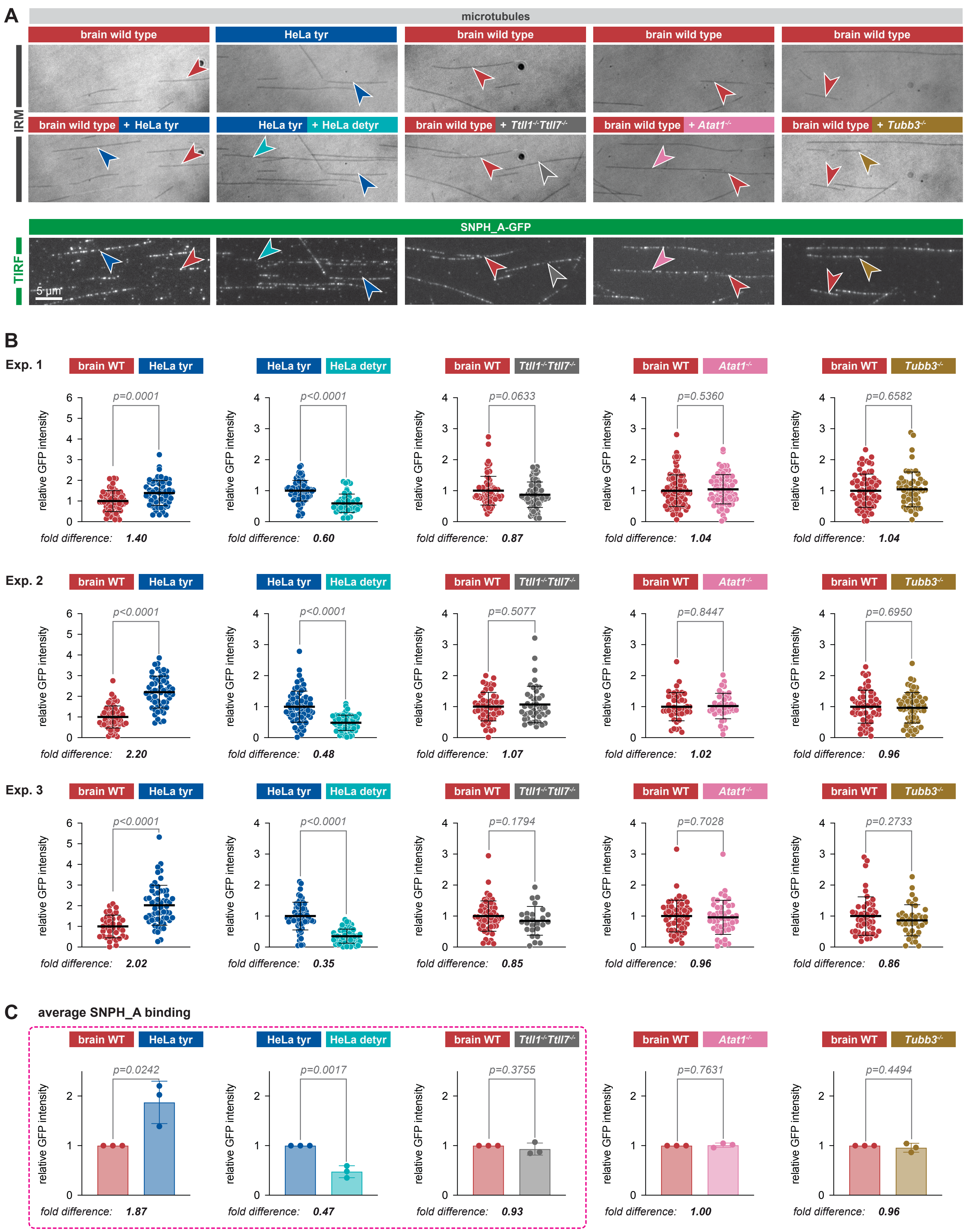
A
microtubules
brain wild type
HeLa tyr
brain wild type
brain wild type
brain wild type
IRM
brain wild type + HeLa tyr
HeLa tyr + HeLa detyr
brain wild type + Ttll1-/-Ttll7-/-
brain wild type + Atat1-/-
brain wild type + Tubb3-/-
SNPH_A-GFP
TIRF
5 µm
B
Exp. 1
brain WT HeLa tyr
p=0.0001
fold difference: 1.40
HeLa tyr HeLa detyr
p<0.0001
fold difference: 0.60
brain WT Ttll1-/-Ttll7-/-
p=0.0633
fold difference: 0.87
brain WT Atat1-/-
p=0.5360
fold difference: 1.04
brain WT Tubb3-/-
p=0.6582
fold difference: 1.04
relative GFP intensity
Exp. 2
p<0.0001
fold difference: 2.20
p<0.0001
fold difference: 0.48
p=0.5077
fold difference: 1.07
p=0.8447
fold difference: 1.02
p=0.6950
fold difference: 0.96
Exp. 3
p<0.0001
fold difference: 2.02
p<0.0001
fold difference: 0.35
p=0.1794
fold difference: 0.85
p=0.7028
fold difference: 0.96
p=0.2733
fold difference: 0.86
C
average SNPH_A binding
p=0.0242
fold difference: 1.87
p=0.0017
fold difference: 0.47
p=0.3755
fold difference: 0.93
p=0.7631
fold difference: 1.00
p=0.4494
fold difference: 0.96

↑ Appendix Figure S21: SNPH_A binding to microtubules with distinct tubulin PTMs and isotype compositions.

A) TIRF microscopy-based assay showing SNPH_A binding from cell lysates to Taxol-stabilised microtubules polymerised from tubulin sources with different PTMs and isotype compositions. IRM images show microtubules added sequentially, with corresponding snapshots and TIRF images displaying SNPH_A binding.

B) Quantification of SNPH_A-GFP intensities in three independent assays. Fluorescence values are normalised to wild-type microtubules (either wild-type brain or wild-type HeLa). Each data point represents a single microtubule. Bars represent mean ± standard deviation.

C) Average SNPH_A binding to different microtubule types. Each data point represents the mean value from one independent assay. Figure elements (dotted box) are also shown in Fig. 4B-D. **(B,C)** Red indicates wild-type brain microtubules; dark blue, wild-type HeLa; light blue, detyrosinated HeLa; grey, *Ttll1*$^{-/-}$*Ttll7*$^{-/-}$; pink, *Atat1*$^{-/-}$ and golden brown, *Tubb3*$^{-/-}$ mouse brain-derived microtubules. Statistical significance was determined using unpaired t-tests; p-values are shown above each comparison, and fold differences are indicated below. All experiments were performed in triplicate using independently purified tubulin and SNPH_A lysates prepared from three separate cell batches.

Krishnan et al. Appendix Figure S22

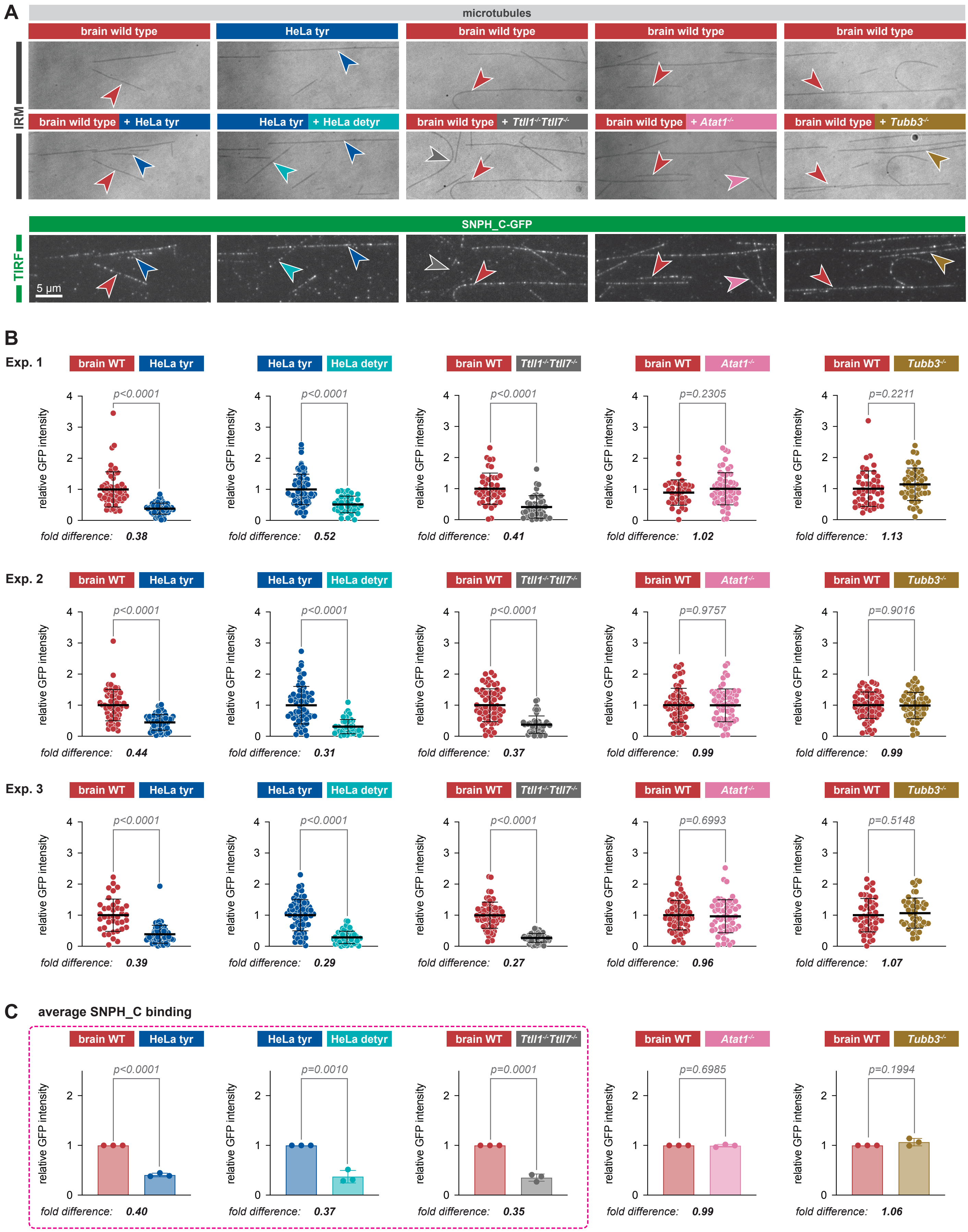

↑ Appendix Figure S22: SNPH_C binding to microtubules with distinct tubulin PTMs and isotype compositions.

A) TIRF microscopy-based assay showing SNPH_C binding from cell lysates to Taxol-stabilised microtubules polymerised from tubulin sources with different PTMs and isotype compositions. IRM images show microtubules added sequentially, with corresponding snapshots and TIRF images displaying SNPH_C binding.

B) Quantification of SNPH_C-GFP intensities in three independent assays. Fluorescence values are normalised to wild-type microtubules (either wild-type brain or wild-type HeLa). Each data point represents a single microtubule. Bars represent mean ± standard deviation.

C) Average SNPH_C binding to different microtubule types. Each data point represents the mean value from one independent assay. Figure elements (dotted box) are also shown in Fig. 4B-D. **(B,C)** Red indicates wild-type brain microtubules; dark blue, wild-type HeLa; light blue, detyrosinated HeLa; grey, *Ttll1*$^{-/-}$*Ttll7*$^{-/-}$; pink, *Atat1*$^{-/-}$ and golden brown, *Tubb3*$^{-/-}$ mouse brain-derived microtubules. Statistical significance was determined using unpaired t-tests; p-values are shown above each comparison, and fold differences are indicated below. All experiments were performed in triplicate using independently purified tubulin and SNPH_C lysates prepared from three separate cell batches.

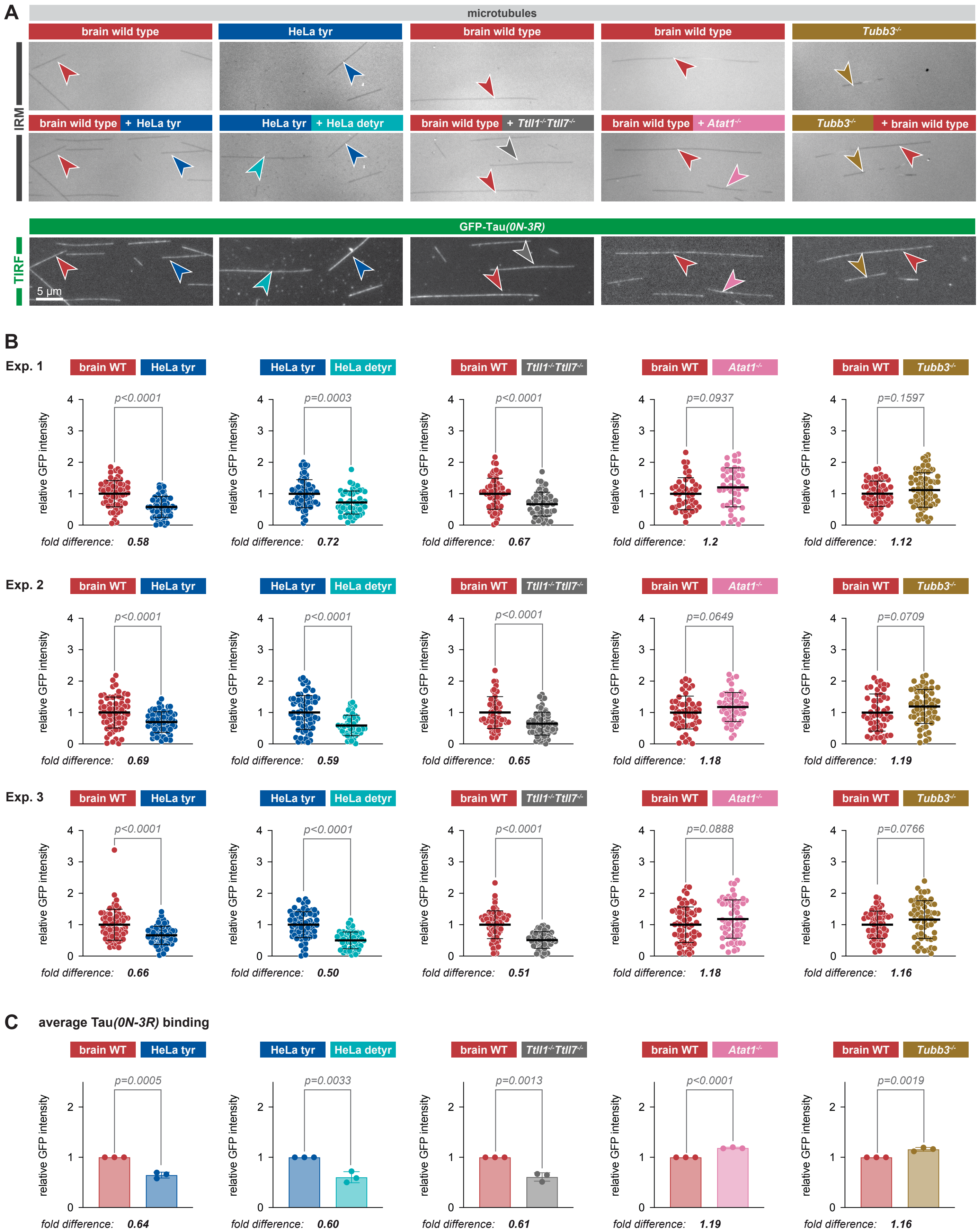
A
microtubules
brain wild type
HeLa tyr
brain wild type
brain wild type
Tubb3-/-
IRM
brain wild type + HeLa tyr
HeLa tyr + HeLa detyr
brain wild type + Ttll1-/-Ttll7-/-
brain wild type + Atat1-/-
Tubb3-/- + brain wild type
GFP-Tau(0N-3R)
TIRF
5 µm
B
Exp. 1
brain WT HeLa tyr
p<0.0001
fold difference: 0.58
HeLa tyr HeLa detyr
p=0.0003
fold difference: 0.72
brain WT Ttll1-/-Ttll7-/-
p<0.0001
fold difference: 0.67
brain WT Atat1-/-
p=0.0937
fold difference: 1.2
brain WT Tubb3-/-
p=0.1597
fold difference: 1.12
Exp. 2
p<0.0001
fold difference: 0.69
p<0.0001
fold difference: 0.59
p<0.0001
fold difference: 0.65
p=0.0649
fold difference: 1.18
p=0.0709
fold difference: 1.19
Exp. 3
p<0.0001
fold difference: 0.66
p<0.0001
fold difference: 0.50
p<0.0001
fold difference: 0.51
p=0.0888
fold difference: 1.18
p=0.0766
fold difference: 1.16
relative GFP intensity
C average Tau(0N-3R) binding
p=0.0005
fold difference: 0.64
p=0.0033
fold difference: 0.60
p=0.0013
fold difference: 0.61
p<0.0001
fold difference: 1.19
p=0.0019
fold difference: 1.16

↑ Appendix Figure S23: Tau*(0N-3R)* binding to microtubules with distinct tubulin PTMs and isotype compositions.

A) TIRF microscopy-based assay showing Tau*(0N-3R)* binding from cell lysates to GMPCPP-stabilised microtubules polymerised from tubulin sources with different PTMs and isotype compositions. IRM images show microtubules added sequentially, with corresponding snapshots and TIRF images displaying Tau binding.

B) Quantification of GFP-Tau*(0N-3R)* intensities in three independent assays. Fluorescence values are normalised to wild-type microtubules (either wild-type brain or wild-type HeLa). Each data point represents a single microtubule. Bars represent mean ± standard deviation.

C) Average Tau*(0N-3R)* binding to different microtubule types. Each data point represents the mean value from one independent assay. **(B,C)** Red indicates wild-type brain microtubules; dark blue, wild-type HeLa; light blue, detyrosinated HeLa; grey, *Ttll1*$^{-/-}$*Ttll7*$^{-/-}$; pink, *Atat1*$^{-/-}$ and golden brown, *Tubb3*$^{-/-}$ mouse brain-derived microtubules. Statistical significance was determined using unpaired t-tests; p-values are shown above each comparison, and fold differences are indicated below. All experiments were performed in triplicate using independently purified tubulin and Tau*(0N-3R)* lysates prepared from three separate cell batches.

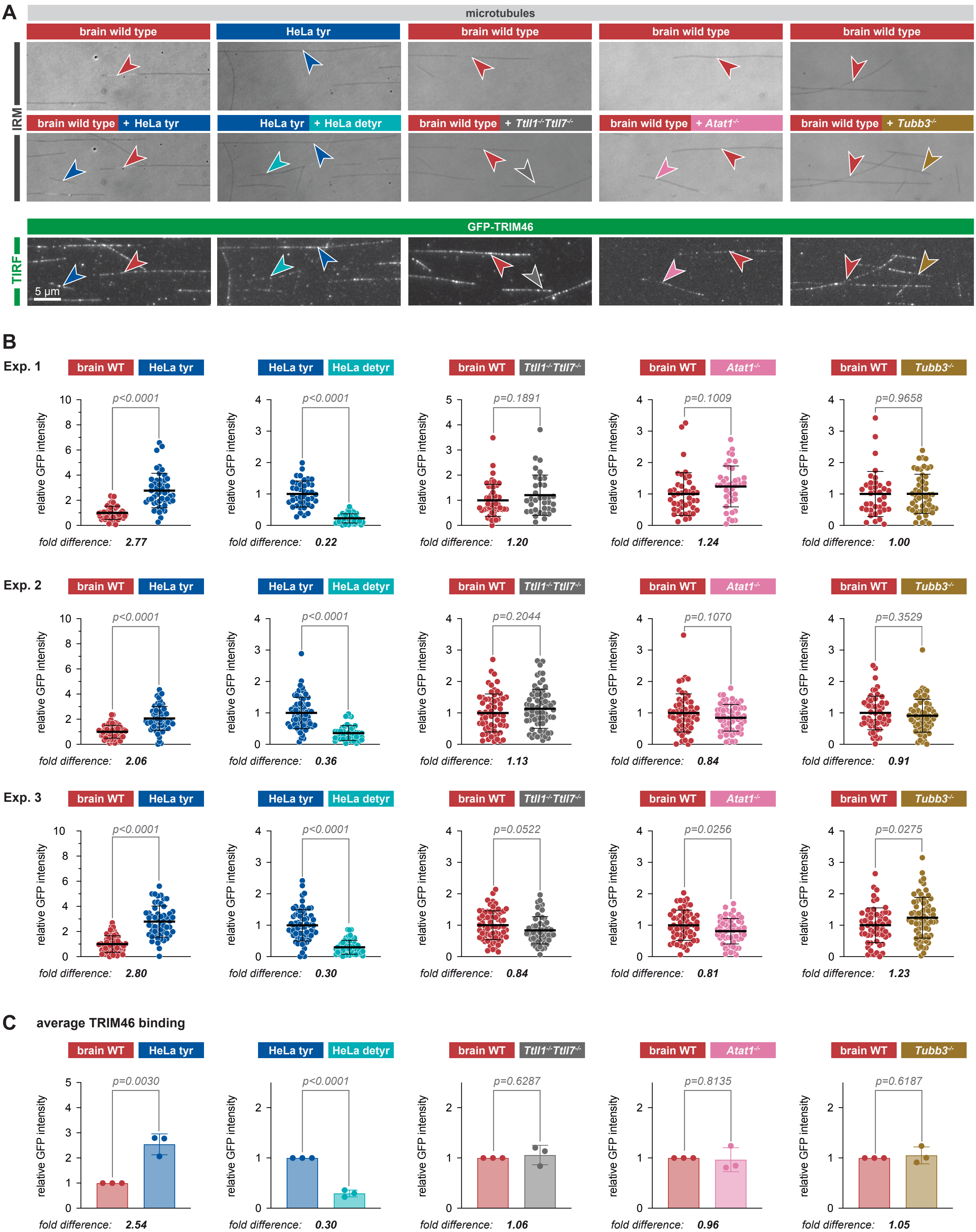
A
microtubules
IRM
brain wild type
HeLa tyr
brain wild type
brain wild type
brain wild type
brain wild type + HeLa tyr
HeLa tyr + HeLa detyr
brain wild type + Ttll1-/-Ttll7-/-
brain wild type + Atat1-/-
brain wild type + Tubb3-/-
GFP-TRIM46
TIRF
5 µm
B
Exp. 1
brain WT HeLa tyr
HeLa tyr HeLa detyr
brain WT Ttll1-/-Ttll7-/-
brain WT Atat1-/-
brain WT Tubb3-/-
relative GFP intensity
p<0.0001
p<0.0001
p=0.1891
p=0.1009
p=0.9658
fold difference: 2.77
fold difference: 0.22
fold difference: 1.20
fold difference: 1.24
fold difference: 1.00
Exp. 2
p<0.0001
p<0.0001
p=0.2044
p=0.1070
p=0.3529
fold difference: 2.06
fold difference: 0.36
fold difference: 1.13
fold difference: 0.84
fold difference: 0.91
Exp. 3
p<0.0001
p<0.0001
p=0.0522
p=0.0256
p=0.0275
fold difference: 2.80
fold difference: 0.30
fold difference: 0.84
fold difference: 0.81
fold difference: 1.23
C average TRIM46 binding
p=0.0030
p<0.0001
p=0.6287
p=0.8135
p=0.6187
fold difference: 2.54
fold difference: 0.30
fold difference: 1.06
fold difference: 0.96
fold difference: 1.05

↑ Appendix Figure S24: TRIM46 binding to microtubules with distinct tubulin PTMs and isotype compositions.

A) TIRF microscopy-based assay showing TRIM46 binding from cell lysates to Taxol-stabilised microtubules polymerised from tubulin sources with different PTMs and isotype compositions. IRM images show microtubules added sequentially, with corresponding snapshots and TIRF images displaying TRIM46 binding.

B) Quantification of GFP-TRIM46 intensities in three independent assays. Fluorescence values are normalised to wild-type microtubules (either wild-type brain or wild-type HeLa). Each data point represents a single microtubule. Bars represent mean ± standard deviation.

C) Average TRIM46 binding to different microtubule types. Each data point represents the mean value from one independent assay. **(B,C)** Red indicates wild-type brain microtubules; dark blue, wild-type HeLa; light blue, detyrosinated HeLa; grey, *Ttll1*$^{-/-}$*Ttll7*$^{-/-}$; pink, *Atat1*$^{-/-}$ and golden brown, *Tubb3*$^{-/-}$ mouse brain-derived microtubules. Statistical significance was determined using unpaired t-tests; p-values are shown above each comparison, and fold differences are indicated below. All experiments were performed in triplicate using independently purified tubulin and TRIM46 lysates prepared from three separate cell batches.

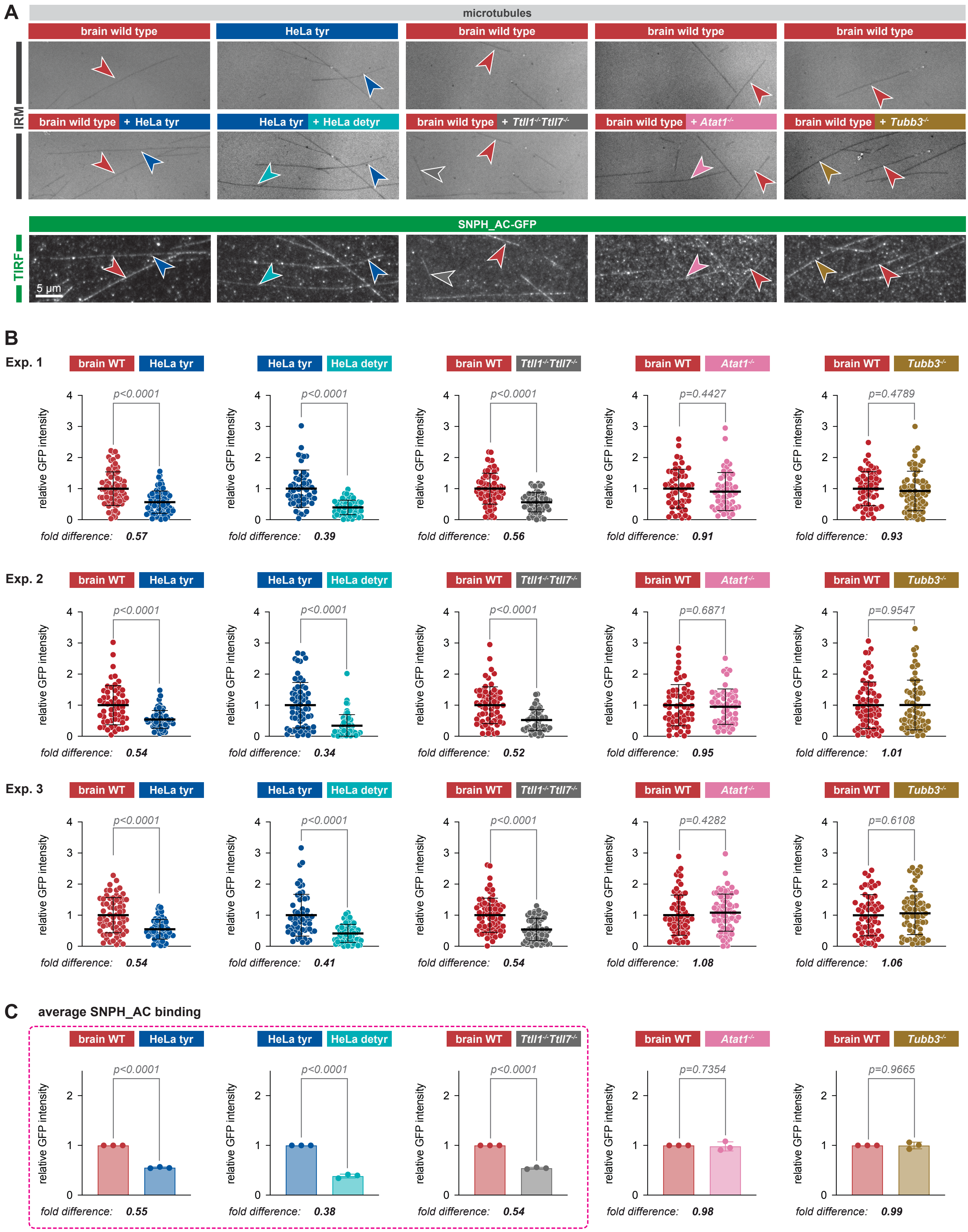
A
microtubules
brain wild type
HeLa tyr
brain wild type
brain wild type
brain wild type
IRM
brain wild type + HeLa tyr
HeLa tyr + HeLa detyr
brain wild type + Ttll1-/-Ttll7-/-
brain wild type + Atat1-/-
brain wild type + Tubb3-/-
SNPH_AC-GFP
TIRF
5 µm
B
Exp. 1
brain WT HeLa tyr p<0.0001 fold difference: 0.57
HeLa tyr HeLa detyr p<0.0001 fold difference: 0.39
brain WT Ttll1-/-Ttll7-/- p<0.0001 fold difference: 0.56
brain WT Atat1-/- p=0.4427 fold difference: 0.91
brain WT Tubb3-/- p=0.4789 fold difference: 0.93
Exp. 2
brain WT HeLa tyr p<0.0001 fold difference: 0.54
HeLa tyr HeLa detyr p<0.0001 fold difference: 0.34
brain WT Ttll1-/-Ttll7-/- p<0.0001 fold difference: 0.52
brain WT Atat1-/- p=0.6871 fold difference: 0.95
brain WT Tubb3-/- p=0.9547 fold difference: 1.01
Exp. 3
brain WT HeLa tyr p<0.0001 fold difference: 0.54
HeLa tyr HeLa detyr p<0.0001 fold difference: 0.41
brain WT Ttll1-/-Ttll7-/- p<0.0001 fold difference: 0.54
brain WT Atat1-/- p=0.4282 fold difference: 1.08
brain WT Tubb3-/- p=0.6108 fold difference: 1.06
relative GFP intensity
C
average SNPH_AC binding
brain WT HeLa tyr p<0.0001 fold difference: 0.55
HeLa tyr HeLa detyr p<0.0001 fold difference: 0.38
brain WT Ttll1-/-Ttll7-/- p<0.0001 fold difference: 0.54
brain WT Atat1-/- p=0.7354 fold difference: 0.98
brain WT Tubb3-/- p=0.9665 fold difference: 0.99

↑ Appendix Figure S25: Binding of the chimeric SNPH_AC chimera microtubules with distinct tubulin PTMs and isotype compositions.

A) TIRF microscopy-based assay showing the binding of the chimeric SNPH_AC chimera from cell lysates to Taxol-stabilised microtubules polymerised from tubulin sources with different PTMs and isotype compositions. IRM images show microtubules added sequentially, with corresponding snapshots and TIRF images displaying binding of the chimeric construct.

B) Quantification of SNPH_AC-GFP intensities in three independent assays. Fluorescence values are normalised to wild-type microtubules (either wild-type brain or wild-type HeLa). Each data point represents a single microtubule. Bars represent mean ± standard deviation.

C) Average binding of the chimeric the chimeric SNPH_AC chimera construct to different microtubule types. Each data point represents the mean value from one independent assay. Figure elements (dotted box) are also shown in Fig. 4B-D. **(B,C)** Red indicates wild-type brain microtubules; dark blue, wild-type HeLa; light blue, detyrosinated HeLa; grey, *Ttll1*$^{-/-}$*Ttll7*$^{-/-}$; pink, *Atat1*$^{-/-}$ and golden brown, *Tubb3*$^{-/-}$ mouse brain-derived microtubules. Statistical significance was determined using unpaired t-tests; p-values are shown above each comparison, and fold differences are indicated below. All experiments were performed in triplicate using independently purified tubulin and chimeric construct lysates prepared from three separate cell batches.

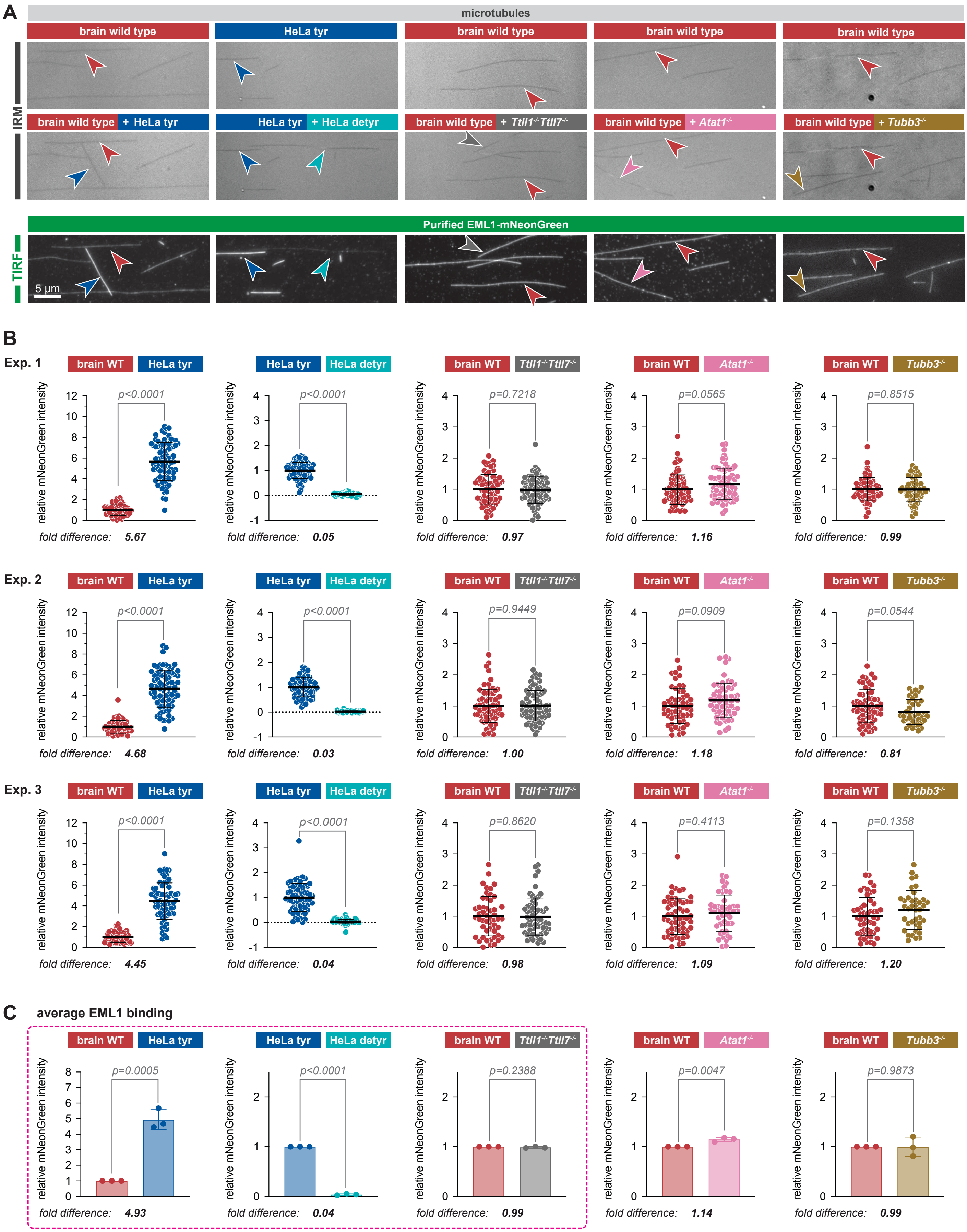
A
microtubules
brain wild type
HeLa tyr
brain wild type
brain wild type
brain wild type
IRM
brain wild type + HeLa tyr
HeLa tyr + HeLa detyr
brain wild type + Ttll1-/-Ttll7-/-
brain wild type + Atat1-/-
brain wild type + Tubb3-/-
Purified EML1-mNeonGreen
TIRF
5 µm
B
Exp. 1
Exp. 2
Exp. 3
brain WT
HeLa tyr
HeLa detyr
Ttll1-/-Ttll7-/-
Atat1-/-
Tubb3-/-
relative mNeonGreen intensity
p<0.0001
p=0.7218
p=0.0565
p=0.8515
fold difference: 5.67
fold difference: 0.05
fold difference: 0.97
fold difference: 1.16
fold difference: 0.99
p=0.9449
p=0.0909
p=0.0544
fold difference: 4.68
fold difference: 0.03
fold difference: 1.00
fold difference: 1.18
fold difference: 0.81
p=0.8620
p=0.4113
p=0.1358
fold difference: 4.45
fold difference: 0.04
fold difference: 0.98
fold difference: 1.09
fold difference: 1.20
C
average EML1 binding
p=0.0005
p<0.0001
p=0.2388
p=0.0047
p=0.9873
fold difference: 4.93
fold difference: 0.04
fold difference: 0.99
fold difference: 1.14
fold difference: 0.99

↑ Appendix Figure S26: Purified EML1 binding to microtubules with distinct tubulin PTMs and isotype compositions.

A) TIRF microscopy-based assay showing the binding of ~100 nM of recombinant EML1-mNeonGreen protein, purified from insect cells, to Taxol-stabilised microtubules polymerised from tubulin sources with different PTMs and isotype compositions. IRM images show microtubules added sequentially, with corresponding snapshots and TIRF images displaying EML1 binding.

B) Quantification of purified EML1-mNeonGreen intensities in three independent assays. Fluorescence values are normalised to wild-type microtubules (either wild-type brain or wild-type HeLa). Each data point represents a single microtubule. Bars represent mean ± standard deviation.

C) Average EML1 binding to different microtubule types. Each data point represents the mean value from one independent assay. Figure elements (dotted box) are also shown in Fig. EV2B-D. **(B,C)** Red indicates wild-type brain microtubules; dark blue, wild-type HeLa; light blue, detyrosinated HeLa; grey, *Ttll1*$^{-/-}$*Ttll7*$^{-/-}$; pink, *Atat1*$^{-/-}$; and golden brown, *Tubb3*$^{-/-}$ mouse brain-derived microtubules. Statistical significance was determined using unpaired t-tests; p-values are shown above each comparison, and fold differences are indicated below. All experiments were performed in triplicate using independently purified tubulin and EML1 protein from a single purification batch.

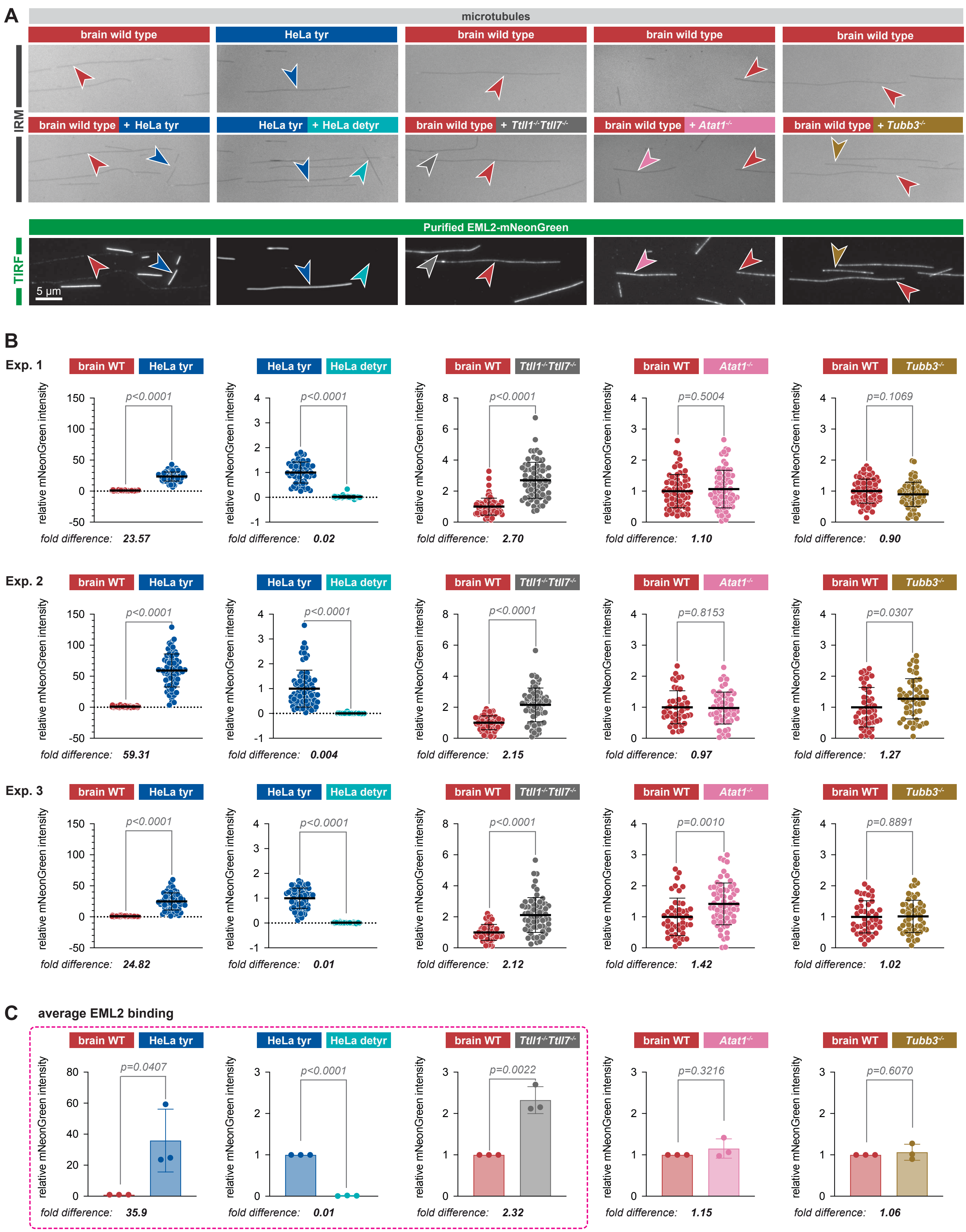
A
microtubules
brain wild type
HeLa tyr
brain wild type
brain wild type
brain wild type
IRM
brain wild type + HeLa tyr
HeLa tyr + HeLa detyr
brain wild type + Ttll1-/-Ttll7-/-
brain wild type + Atat1-/-
brain wild type + Tubb3-/-
Purified EML2-mNeonGreen
TIRF
5 µm
B
Exp. 1
brain WT HeLa tyr
p<0.0001
fold difference: 23.57
HeLa tyr HeLa detyr
p<0.0001
fold difference: 0.02
brain WT Ttll1-/-Ttll7-/-
p<0.0001
fold difference: 2.70
brain WT Atat1-/-
p=0.5004
fold difference: 1.10
brain WT Tubb3-/-
p=0.1069
fold difference: 0.90
Exp. 2
p<0.0001
fold difference: 59.31
p<0.0001
fold difference: 0.004
p<0.0001
fold difference: 2.15
p=0.8153
fold difference: 0.97
p=0.0307
fold difference: 1.27
Exp. 3
p<0.0001
fold difference: 24.82
p<0.0001
fold difference: 0.01
p<0.0001
fold difference: 2.12
p=0.0010
fold difference: 1.42
p=0.8891
fold difference: 1.02
relative mNeonGreen intensity
C
average EML2 binding
brain WT HeLa tyr
p=0.0407
fold difference: 35.9
HeLa tyr HeLa detyr
p<0.0001
fold difference: 0.01
brain WT Ttll1-/-Ttll7-/-
p=0.0022
fold difference: 2.32
brain WT Atat1-/-
p=0.3216
fold difference: 1.15
brain WT Tubb3-/-
p=0.6070
fold difference: 1.06

↑ Appendix Figure S27: Purified EML2 binding to microtubules with distinct tubulin PTMs and isotype compositions.

A) TIRF microscopy-based assay showing the binding of recombinant ~40 nM EML2-mNeonGreen protein, purified from insect cells, to Taxol-stabilised microtubules polymerised from tubulin sources with different PTMs and isotype compositions. IRM images show microtubules added sequentially, with corresponding snapshots and TIRF images displaying EML2 binding.

B) Quantification of purified EML2-mNeonGreen intensities in three independent assays. Fluorescence values are normalised to wild-type microtubules (either wild-type brain or wild-type HeLa). Each data point represents a single microtubule. Bars represent mean ± standard deviation.

C) Average EML2 binding to different microtubule types. Each data point represents the mean value from one independent assay. Figure elements (dotted box) are also shown in Fig. EV2B-D. **(B,C)** Red indicates wild-type brain microtubules; dark blue, wild-type HeLa; light blue, detyrosinated HeLa; grey, *Ttll1*$^{-/-}$*Ttll7*$^{-/-}$; pink, *Atat1*$^{-/-}$; and golden brown, *Tubb3*$^{-/-}$ mouse brain-derived microtubules. Statistical significance was determined using unpaired t-tests; p-values are shown above each comparison, and fold differences are indicated below. All experiments were performed in triplicate using independently purified tubulin and EML2 protein from a single purification batch.

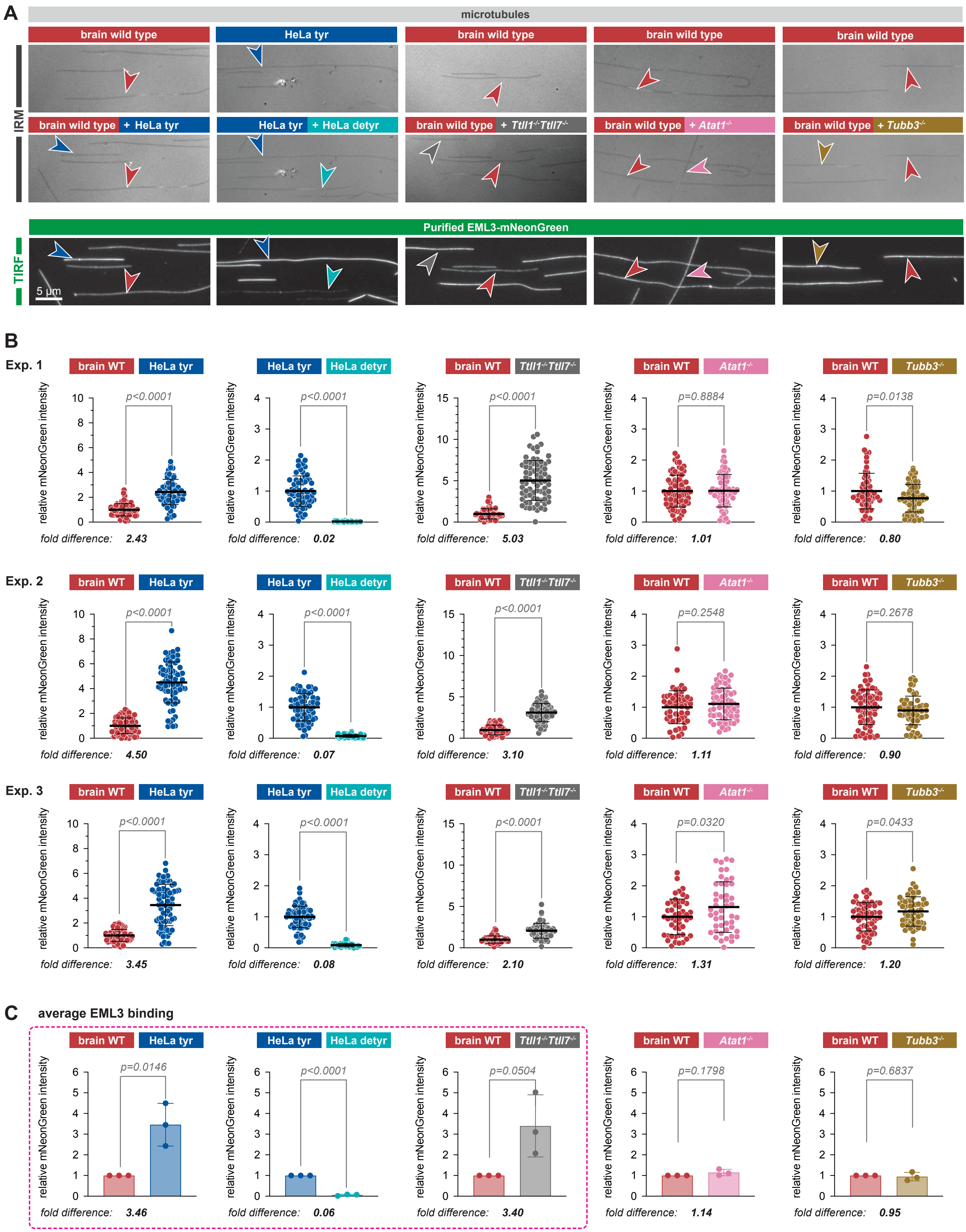
A
microtubules
brain wild type
HeLa tyr
brain wild type
brain wild type
brain wild type
IRM
brain wild type + HeLa tyr
HeLa tyr + HeLa detyr
brain wild type + Ttll1-/-Ttll7-/-
brain wild type + Atat1-/-
brain wild type + Tubb3-/-
Purified EML3-mNeonGreen
TIRF
5 µm
B
Exp. 1
brain WT HeLa tyr
p<0.0001
fold difference: 2.43
HeLa tyr HeLa detyr
p<0.0001
fold difference: 0.02
brain WT Ttll1-/-Ttll7-/-
p<0.0001
fold difference: 5.03
brain WT Atat1-/-
p=0.8884
fold difference: 1.01
brain WT Tubb3-/-
p=0.0138
fold difference: 0.80
Exp. 2
brain WT HeLa tyr
p<0.0001
fold difference: 4.50
HeLa tyr HeLa detyr
p<0.0001
fold difference: 0.07
brain WT Ttll1-/-Ttll7-/-
p<0.0001
fold difference: 3.10
brain WT Atat1-/-
p=0.2548
fold difference: 1.11
brain WT Tubb3-/-
p=0.2678
fold difference: 0.90
Exp. 3
brain WT HeLa tyr
p<0.0001
fold difference: 3.45
HeLa tyr HeLa detyr
p<0.0001
fold difference: 0.08
brain WT Ttll1-/-Ttll7-/-
p<0.0001
fold difference: 2.10
brain WT Atat1-/-
p=0.0320
fold difference: 1.31
brain WT Tubb3-/-
p=0.0433
fold difference: 1.20
relative mNeonGreen intensity
C
average EML3 binding
brain WT HeLa tyr
p=0.0146
fold difference: 3.46
HeLa tyr HeLa detyr
p<0.0001
fold difference: 0.06
brain WT Ttll1-/-Ttll7-/-
p=0.0504
fold difference: 3.40
brain WT Atat1-/-
p=0.1798
fold difference: 1.14
brain WT Tubb3-/-
p=0.6837
fold difference: 0.95

🡩 Appendix Figure S28: Purified EML3 binding to microtubules with distinct tubulin PTMs and isotype compositions.

A) TIRF microscopy-based assay showing the binding of ~35 nM recombinant EML3-mNeonGreen protein, purified from insect cells, to Taxol-stabilised microtubules polymerised from tubulin sources with different PTMs and isotype compositions. IRM images show microtubules added sequentially, with corresponding snapshots and TIRF images displaying EML3 binding.

B) Quantification of purified EML3-mNeonGreen intensities in three independent assays. Fluorescence values are normalised to wild-type microtubules (either wild-type brain or wild-type HeLa). Each data point represents a single microtubule. Bars represent mean ± standard deviation.

C) Average EML3 binding to different microtubule types. Each data point represents the mean value from one independent assay. Figure elements (dotted box) are also shown in Fig. EV2B-D. **(B,C)** Red indicates wild-type brain microtubules; dark blue, wild-type HeLa; light blue, detyrosinated HeLa; grey, *Ttll1*$^{-/-}$*Ttll7*$^{-/-}$; pink, *Atat1*$^{-/-}$ and golden brown, *Tubb3*$^{-/-}$ mouse brain-derived microtubules. Statistical significance was determined using unpaired t-tests; p-values are shown above each comparison, and fold differences are indicated below. All experiments were performed in triplicate using independently purified tubulin and EML3 protein from a single purification batch.

Krishnan et al. Appendix Figure S29

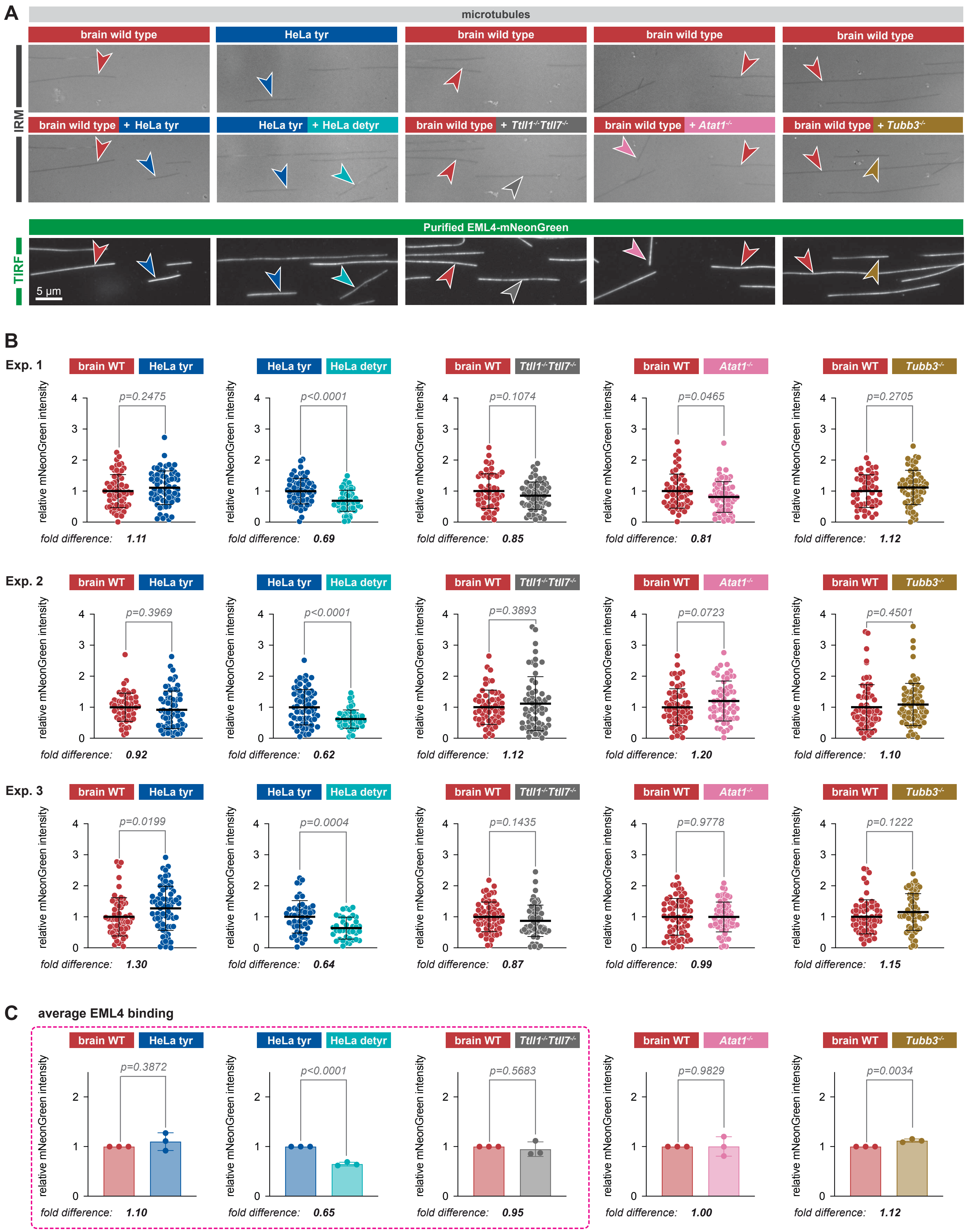

↑ Appendix Figure S29: Purified EML4 binding to microtubules with distinct tubulin PTMs and isotype compositions.

A) TIRF microscopy-based assay showing the binding of ~15 nM recombinant EML4-mNeonGreen protein, purified from insect cells, to Taxol-stabilised microtubules polymerised from tubulin sources with different PTMs and isotype compositions. IRM images show microtubules added sequentially, with corresponding snapshots and TIRF images displaying EML4 binding.

B) Quantification of purified EML4-mNeonGreen intensities in three independent assays. Fluorescence values are normalised to wild-type microtubules (either wild-type brain or wild-type HeLa). Each data point represents a single microtubule. Bars represent mean ± standard deviation.

C) Average EML4 binding to different microtubule types. Each data point represents the mean value from one independent assay. Figure elements (dotted box) are also shown in Fig. EV2B-D. **(B,C)** Red indicates wild-type brain microtubules; dark blue, wild-type HeLa; light blue, detyrosinated HeLa; grey, *Ttll1*$^{-/-}$*Ttll7*$^{-/-}$; pink, *Atat1*$^{-/-}$; and golden brown, *Tubb3*$^{-/-}$ mouse brain-derived microtubules. Statistical significance was determined using unpaired t-tests; p-values are shown above each comparison, and fold differences are indicated below. All experiments were performed in triplicate using independently purified tubulin and EML4 protein from a single purification batch.

Appendix References

Azimzadeh J, Hergert P, Delouvee A, Euteneuer U, Formstecher E, Khodjakov A, Bornens M (2009) hPOC5 is a centrin-binding protein required for assembly of full-length centrioles. *J Cell Biol* **185:** 101-114

Bernier G, Brown A, Dalpe G, De Repentigny Y, Mathieu M, Kothary R (1995) Dystonin expression in the developing nervous system predominates in the neurons that degenerate in dystonia musculorum mutant mice. *Mol Cell Neurosci* **6:** 509-520

Bernier G, Mathieu M, De Repentigny Y, Vidal SM, Kothary R (1996) Cloning and characterization of mouse ACF7, a novel member of the dystonin subfamily of actin binding proteins. *Genomics* **38:** 19-29

Bieling P, Telley IA, Surrey T (2010) A minimal midzone protein module controls formation and length of antiparallel microtubule overlaps. *Cell* **142:** 420-432

Bosc C, Cronk JD, Pirollet F, Watterson DM, Haiech J, Job D, Margolis RL (1996) Cloning, expression, and properties of the microtubule-stabilizing protein STOP. *Proc Natl Acad Sci U S A* **93:** 2125-2130

Canty JT, Hensley A, Aslan M, Jack A, Yildiz A (2023) TRAK adaptors regulate the recruitment and activation of dynein and kinesin in mitochondrial transport. *Nat Commun* **14:** 1376

Chapin SJ, Lue CM, Yu MT, Bulinski JC (1995) Differential expression of alternatively spliced forms of MAP4: a repertoire of structurally different microtubule-binding domains. *Biochemistry* **34:** 2289-2301

Cleveland DW, Hwo SY, Kirschner MW (1977a) Physical and chemical properties of purified tau factor and the role of tau in microtubule assembly. *J Mol Biol* **116:** 227-247

Cleveland DW, Hwo SY, Kirschner MW (1977b) Purification of tau, a microtubule-associated protein that induces assembly of microtubules from purified tubulin. *J Mol Biol* **116:** 207-225

Conkar D, Culfa E, Odabasi E, Rauniyar N, Yates JR, 3rd, Firat-Karalar EN (2017) The centriolar satellite protein CCDC66 interacts with CEP290 and functions in cilium formation and trafficking. *J Cell Sci* **130:** 1450-1462

Dacheux D, Roger B, Bosc C, Landrein N, Roche E, Chansel L, Trian T, Andrieux A, Papaxanthos-Roche A, Marthan R et al (2015) Human FAM154A (SAXO1) is a microtubule-stabilizing protein specific to cilia and related structures. *J Cell Sci* **128:** 1294-1307

Dafinger C, Liebau MC, Elsayed SM, Hellenbroich Y, Boltshauser E, Korenke GC, Fabretti F, Janecke AR, Ebermann I, Nurnberg G et al (2011) Mutations in KIF7 link Joubert syndrome with Sonic Hedgehog signaling and microtubule dynamics. *J Clin Invest* **121:** 2662-2667

Datta P, Cribbs JT, Seo S (2021) Differential requirement of NPHP1 for compartmentalized protein localization during photoreceptor outer segment development and maintenance. *PLoS One* **16:** e0246358

Di Benedetto M, Pineau P, Nouet S, Berhouet S, Seitz I, Louis S, Dejean A, Couraud PO, Strosberg AD, Stoppa-Lyonnet D et al (2006) Mutation analysis of the 8p22 candidate

tumor suppressor gene ATIP/MTUS1 in hepatocellular carcinoma. *Mol Cell Endocrinol* **252:** 207-215

Dougherty GW, Adler HJ, Rzadzinska A, Gimona M, Tomita Y, Lattig MC, Merritt RC, Jr., Kachar B (2005) CLAMP, a novel microtubule-associated protein with EB-type calponin homology. *Cell Motil Cytoskeleton* **62:** 141-156

Eudy JD, Ma-Edmonds M, Yao SF, Talmadge CB, Kelley PM, Weston MD, Kimberling WJ, Sumegi J (1997) Isolation of a novel human homologue of the gene coding for echinoderm microtubule-associated protein (EMAP) from the Usher syndrome type 1a locus at 14q32. *Genomics* **43:** 104-106

Fong K-W, Leung JW-C, Li Y, Wang W, Feng L, Ma W, Liu D, Songyang Z, Chen J (2013) MTR120/KIAA1383, a novel microtubule-associated protein, promotes microtubule stability and ensures cytokinesis. *J Cell Sci* **126:** 825-837

Francis F, Koulakoff A, Boucher D, Chafey P, Schaar B, Vinet MC, Friocourt G, McDonnell N, Reiner O, Kahn A et al (1999) Doublecortin is a developmentally regulated, microtubule-associated protein expressed in migrating and differentiating neurons. *Neuron* **23:** 247-256.

Garner CC, Brugg B, Matus A (1988) A 70-kilodalton microtubule-associated protein (MAP2c), related to MAP2. *J Neurochem* **50:** 609-615

Garner CC, Garner A, Huber G, Kozak C, Matus A (1990) Molecular cloning of microtubule-associated protein 1 (MAP1A) and microtubule-associated protein 5 (MAP1B): identification of distinct genes and their differential expression in developing brain. *J Neurochem* **55:** 146-154

Gleeson JG, Lin PT, Flanagan LA, Walsh CA (1999) Doublecortin is a microtubule-associated protein and is expressed widely by migrating neurons. *Neuron* **23:** 257-271

Gundersen GG, Kalnoski MH, Bulinski JC (1984) Distinct populations of microtubules: tyrosinated and nontyrosinated alpha tubulin are distributed differently in vivo. *Cell* **38:** 779-789

Henrichs V, Grycova L, Barinka C, Nahacka Z, Neuzil J, Diez S, Rohlena J, Braun M, Lansky Z (2020) Mitochondria-adaptor TRAK1 promotes kinesin-1 driven transport in crowded environments. *Nat Commun* **11:** 3123

Hooikaas PJ, Martin M, Muhlethaler T, Kuijntjes G-J, Peeters CAE, Katrukha EA, Ferrari L, Stucchi R, Verhagen DGF, van Riel WE et al (2019) MAP7 family proteins regulate kinesin-1 recruitment and activation. *J Cell Biol* **218:** 1298-1318

Hotta T, McAlear TS, Yue Y, Higaki T, Haynes SE, Nesvizhskii AI, Sept D, Verhey KJ, Bechstedt S, Ohi R (2022) EML2-S constitutes a new class of proteins that recognizes and regulates the dynamics of tyrosinated microtubules. *Curr Biol* **32:** 3898-3910 e3814

Jakobsen L, Vanselow K, Skogs M, Toyoda Y, Lundberg E, Poser I, Falkenby LG, Bennetzen M, Westendorf J, Nigg EA et al (2011) Novel asymmetrically localizing components of human centrosomes identified by complementary proteomics methods. *EMBO J* **30:** 1520-1535

Jijumon AS, Bodakuntla S, Genova M, Bangera M, Sackett V, Besse L, Maksut F, Henriot V, Magiera MM, Sirajuddin M et al (2022) Lysate-based pipeline to characterize microtubule-associated proteins uncovers unique microtubule behaviours. *Nat Cell Biol* **24:** 253–267

Jin Y, Murakumo Y, Ueno K, Hashimoto M, Watanabe T, Shimoyama Y, Ichihara M, Takahashi M (2004) Identification of a mouse cytoskeleton-associated protein, CKAP2, with microtubule-stabilizing properties. *Cancer Sci* **95:** 815-821

Kang J-S, Tian J-H, Pan P-Y, Zald P, Li C, Deng C, Sheng Z-H (2008) Docking of axonal mitochondria by syntaphilin controls their mobility and affects short-term facilitation. *Cell* **132:** 137-148

Karpova N, Bobinnec Y, Fouix S, Huitorel P, Debec A (2006) Jupiter, a new Drosophila protein associated with microtubules. *Cell Motil Cytoskeleton* **63:** 301-312

Kikuchi K, Sakamoto Y, Uezu A, Yamamoto H, Ishiguro K-I, Shimamura K, Saito T, Hisanaga S-I, Nakanishi H (2022) Map7D2 and Map7D1 facilitate microtubule stabilization through distinct mechanisms in neuronal cells. *Life Sci Alliance* **5:** e202201390

Kilmartin JV, Wright B, Milstein C (1982) Rat monoclonal antitubulin antibodies derived by using a new nonsecreting rat cell line. *J Cell Biol* **93:** 576-582

Lee MK, Tuttle JB, Rebhun LI, Cleveland DW, Frankfurter A (1990) The expression and posttranslational modification of a neuron-specific beta-tubulin isotype during chick embryogenesis. *Cell Motil Cytoskeleton* **17:** 118-132

Lepley DM, Palange JM, Suprenant KA (1999) Sequence and expression patterns of a human EMAP-related protein-2 (HuEMAP-2). *Gene* **237:** 343-349

Leung CL, Sun D, Zheng M, Knowles DR, Liem RK (1999) Microtubule actin cross-linking factor (MACF): a hybrid of dystonin and dystrophin that can interact with the actin and microtubule cytoskeletons. *J Cell Biol* **147:** 1275-1286

Mack GJ, Compton DA (2001) Analysis of mitotic microtubule-associated proteins using mass spectrometry identifies astrin, a spindle-associated protein. *Proc Natl Acad Sci U S A* **98:** 14434-14439

Manabe Ri, Whitmore L, Weiss JM, Horwitz AR (2002) Identification of a Novel Microtubule-Associated Protein that Regulates Microtubule Organization and Cytokinesis by Using a GFP-Screening Strategy. *Curr Biol* **12:** 1946-1951

Maouche-Chretien L, Deleu N, Badoual C, Fraissignes P, Berger R, Gaulard P, Romeo PH, Leroy-Viard K (1998) Identification of a novel cDNA, encoding a cytoskeletal associated protein, differentially expressed in diffuse large B cell lymphomas. *Oncogene* **17:** 1245-1251

Masson D, Kreis TE (1993) Identification and molecular characterization of E-MAP-115, a novel microtubule-associated protein predominantly expressed in epithelial cells. *J Cell Biol* **123:** 357-371

Mendes Maia T, Gogendeau D, Pennetier C, Janke C, Basto R (2014) Bug22 influences cilium morphology and the post-translational modification of ciliary microtubules. *Biol Open* **3:** 138-151

Mollinari C, Kleman J-P, Jiang W, Schoehn G, Hunter T, Margolis RL (2002) PRC1 is a microtubule binding and bundling protein essential to maintain the mitotic spindle midzone. *J Cell Biol* **157:** 1175-1186

Ohta S, Hamada M, Sato N, Toramoto I (2015) Polyglutamylated Tubulin Binding Protein C1orf96/CSAP Is Involved in Microtubule Stabilization in Mitotic Spindles. *PLoS One* **10:** e0142798

Orban-Nemeth Z, Simader H, Badurek S, Trancikova A, Propst F (2005) Microtubule-associated protein 1S, a short and ubiquitously expressed member of the microtubule-associated protein 1 family. *J Biol Chem* **280:** 2257-2265

Paturle-Lafanechere L, Manier M, Trigault N, Pirollet F, Mazarguil H, Job D (1994) Accumulation of delta 2-tubulin, a major tubulin variant that cannot be tyrosinated, in neuronal tissues and in stable microtubule assemblies. *J Cell Sci* **107:** 1529-1543

Perez Y, Bar-Yaacov R, Kadir R, Wormser O, Shelef I, Birk OS, Flusser H, Birnbaum RY (2019) Mutations in the microtubule-associated protein MAP11 (C7orf43) cause microcephaly in humans and zebrafish. *Brain* **142:** 574-585

Piperno G, Fuller MT (1985) Monoclonal antibodies specific for an acetylated form of alpha-tubulin recognize the antigen in cilia and flagella from a variety of organisms. *J Cell Biol* **101:** 2085-2094

Pollmann M, Parwaresch R, Adam-Klages S, Kruse M-L, Buck F, Heidebrecht H-J (2006) Human EML4, a novel member of the EMAP family, is essential for microtubule formation. *Exp Cell Res* **312:** 3241-3251

Rickard JE, Kreis TE (1990) Identification of a novel nucleotide-sensitive microtubule-binding protein in HeLa cells. *J Cell Biol* **110:** 1623-1633

Rodrigues-Ferreira S, Di Tommaso A, Dimitrov A, Cazaubon S, Gruel N, Colasson H, Nicolas A, Chaverot N, Molinie V, Reyal F et al (2009) 8p22 MTUS1 gene product ATIP3 is a novel anti-mitotic protein underexpressed in invasive breast carcinoma of poor prognosis. *PLoS One* **4:** e7239

Rogowski K, van Dijk J, Magiera MM, Bosc C, Deloulme J-C, Bosson A, Peris L, Gold ND, Lacroix B, Bosch Grau M et al (2010) A family of protein-deglutamylating enzymes associated with neurodegeneration. *Cell* **143:** 564-578

Saffin J-M, Venoux M, Prigent C, Espeut J, Poulat F, Giorgi D, Abrieu A, Rouquier S (2005) ASAP, a human microtubule-associated protein required for bipolar spindle assembly and cytokinesis. *Proc Natl Acad Sci U S A* **102:** 11302-11307

Sato Y, Akitsu M, Amano Y, Yamashita K, Ide M, Shimada K, Yamashita A, Hirano H, Arakawa N, Maki T et al (2013) The novel PAR-1-binding protein MTCL1 has crucial roles in organizing microtubules in polarizing epithelial cells. *J Cell Sci* **126:** 4671-4683

Sato Y, Hayashi K, Amano Y, Takahashi M, Yonemura S, Hayashi I, Hirose H, Ohno S, Suzuki A (2014) MTCL1 crosslinks and stabilizes non-centrosomal microtubules on the Golgi membrane. *Nat Commun* **5:** 5266

Scales TME, Lin S, Kraus M, Goold RG, Gordon-Weeks PR (2009) Nonprimed and DYRK1A-primed GSK3 beta-phosphorylation sites on MAP1B regulate microtubule dynamics in growing axons. *J Cell Sci* **122:** 2424-2435

Su K-C, Radul E, Maier NK, Tsang M-J, Goul C, Moodie B, Marescal O, Keys HR, Cheeseman IM (2025) Functional genetics reveals modulators of antimicrotubule drug sensitivity. *J Cell Biol* **224:** e202403065

Sun X, Shi X, Liu M, Li D, Zhang L, Liu X, Zhou J (2011) Mdp3 is a novel microtubule-binding protein that regulates microtubule assembly and stability. *Cell Cycle* **10:** 3929-3937

Tamai K, Sawamura D, Do HC, Tamai Y, Li K, Uitto J (1993) The human 230-kD bullous pemphigoid antigen gene (BPAG1). Exon-intron organization and identification of regulatory tissue specific elements in the promoter region. *J Clin Invest* **92:** 814-822

Tegha-Dunghu J, Neumann B, Reber S, Krause R, Erfle H, Walter T, Held M, Rogers P, Hupfeld K, Ruppert T et al (2008) EML3 is a nuclear microtubule-binding protein required for the correct alignment of chromosomes in metaphase. *J Cell Sci* **121:** 1718-1726

van Beuningen SFB, Will L, Harterink M, Chazeau A, van Battum EY, Frias CP, Franker MAM, Katrukha EA, Stucchi R, Vocking K et al (2015) TRIM46 Controls Neuronal Polarity and Axon Specification by Driving the Formation of Parallel Microtubule Arrays. *Neuron* **88:** 1208-1226

Wehland J, Schroder HC, Weber K (1984) Amino acid sequence requirements in the epitope recognized by the alpha-tubulin-specific rat monoclonal antibody YL 1/2. *EMBO J* **3:** 1295-1300

Weingarten MD, Lockwood AH, Hwo S-Y, Kirschner MW (1975) A protein factor essential for microtubule assembly. *Proc Natl Acad Sci U S A* **72:** 1858-1862

Wong EYM, Tse JYM, Yao K-M, Lui VCH, Tam P-C, Yeung WSB (2004) Identification and characterization of human VCY2-interacting protein: VCY2IP-1, a microtubule-associated protein-like protein. *Biol Reprod* **70:** 775-784

Wu G, Lin Y-T, Wei R, Chen Y, Shan Z, Lee W-H (2008) Hice1, a novel microtubule-associated protein required for maintenance of spindle integrity and chromosomal stability in human cells. *Mol Cell Biol* **28:** 3652-3662

Yumoto T, Nakadate K, Nakamura Y, Sugitani Y, Sugitani-Yoshida R, Ueda S, Sakakibara S-i (2013) Radmis, a novel mitotic spindle protein that functions in cell division of neural progenitors. *PLoS One* **8:** e79895
