# Supplementary material for: Microtubule posttranslational modifications provide unique recognition patterns for associated proteins
Source: EMBO J. 2026 Sep 12;45(20):7274–306. doi: 10.1038/s44318-026-00908-8 (PMC13672295; doi:10.1038/s44318-026-00908-8)
Supplement: Supplementary file 3 [file 44318_2026_908_MOESM2_ESM.pdf]

THE EMBO JOURNAL

Microtubule posttranslational modifications provide unique recognition patterns for associated proteins

Arya Krishnan, Eva Desvigne-Hansch, Veronique Henriot, Alica Dodokova, Sinda Khanfir, Mariya Genova, Gryčová Lenka, Maria Magiera, Marcus Braun, Zdenek Lansky, and Carsten Janke

Corresponding authors: Carsten Janke (Carsten.Janke@curie.fr) , Zdenek Lansky (zdenek.lansky@ibt.cas.cz)

Review Timeline:

Submission Date:	10th Jun 26
Editorial Decision:	17th Jun 26
Revision Received:	22nd Jul 26
Editorial Decision:	18th Aug 26
Revision Received:	21st Aug 26
Accepted:	27th Aug 26

Editor: Ieva Gailite

Transaction Report:

Please note that the manuscript was previously reviewed at another journal and the reports were taken into account in the decision making process at The EMBO Journal.

(Note: With the exception of the correction of typographical or spelling errors that could be a source of ambiguity, letters and reports are not edited. Depending on transfer agreements, referee reports obtained elsewhere may or may not be included in this compilation. Referee reports are anonymous unless the Referee chooses to sign their reports.)

Dear Carsten,

Thank you for submitting your manuscript for consideration by the EMBO Journal together with reviewer reports from another journal. I have now read your manuscript and the reviewer comments.

Based on our editorial assessment, your study would be a good candidate for our Resource section, which does not require as in-depth mechanistic insights as the Article section. Based on our evaluation, the novelty of the study lies in the breadth and the extensiveness of the analysis and its usefulness for other researchers working on this topic, as appropriate for our Resource section. Therefore, I would like to invite you to submit a revised version of the manuscript in response to the comments by both reviewers, including an extension of the introduction and placing the findings more transparently within the context of the published literature.

We generally allow three months as standard revision time, which can be extended to six months in the case of major revisions. Should you foresee a problem in meeting this three-month deadline, please let us know in advance in order to arrange an extension.

As a matter of policy, competing manuscripts published during this period will not negatively impact on our assessment of the conceptual advance presented by your study. However, please contact me as soon as possible upon publication of any related work to discuss the appropriate course of action.

When preparing your letter of response to the referees' comments, please bear in mind that this will form part of the Review Process File and will therefore be available online to the community. For more details on our Transparent Editorial Process, please visit our website: https://link.springer.com/partners/embo-press/editorial-policies#Peer%20review. Please also see the attached instructions for further guidelines on preparation of the revised manuscript.

Please feel free to contact me if you have any further questions regarding the revision. Thank you for the opportunity to consider your work for publication. I look forward to receiving your revised manuscript.

With kind regards,

Ieva

Ieva Gailite, PhD
Senior Scientific Editor
The EMBO Journal
Meyerhofstrasse 1
D-69117 Heidelberg
Tel: +4962218891309
i.gailite@embojournal.org

Instructions for preparing your revised manuscript:

Read our guidance for manuscript revisions and related editorial policies: https://link.springer.com/journal/44318/submission-guidelines#cms-Revised-submissions

Please make sure you upload a letter of response to the referees' comments together with the revised manuscript.

Please also check that the title and abstract of the manuscript are brief, yet explicit, even to non-specialists.

When assembling figures, please refer to our figure preparation guideline in order to ensure proper formatting and readability in print as well as on screen:
https://media.springernature.com/original/springer-cms/rest/v1/content/27825798/data/v1

At EMBO Press we ask authors to provide source data for the main manuscript figures. You will receive a separate email with instructions for providing source data with your revised manuscript, including how to upload and organize the files.

IMPORTANT: When you send the revision we will require
- a point-by-point response to the referees' comments, with a detailed description of the changes made (as a word file).
- a word file of the manuscript text.
- individual production quality figure files (one file per figure)

- a complete author checklist
- Expanded View files (replacing Supplementary Information)
- a Reagents and Tools Table as part of the Methods section

Please remember: Digital image enhancement is acceptable practice, as long as it accurately represents the original data and conforms to community standards. If a figure has been subjected to significant electronic manipulation, this must be noted in the figure legend or in the 'Materials and Methods' section. The editors reserve the right to request original versions of figures and the original images that were used to assemble the figure.

We realize that it is difficult to revise to a specific deadline. In the interest of protecting the conceptual advance provided by the work, we recommend a revision within 3 months (15th Sep 2026). Please discuss the revision progress ahead of this time with the editor if you require more time to complete the revisions.

Krishnan et al.; **Answers to reviewers' comments:**

We would like to thank both referees for their insightful comments, which helped us to substantially improve the manuscript. The replies to the referees' comments are highlighted in blue, while in the manuscript key changes are highlighted in red.

Reviewer #1 (Remarks to the Author):

In this manuscript, Krishnan et al. present a collection of tubulin preparations from different sources, carrying different post-translational modifications (PTMs), and use cell lysates to examine the selectivity of microtubule-associated proteins (MAPs) for microtubules assembled from these preparations. While the biochemical effort involved in purifying tubulin with distinct properties is impressive, the overall contribution falls short of the standards expected for [another journal], for reasons that concern both the novelty and importance of the results and the standard of scholarship.

A central problem is the overclaiming of novelty. The abstract asserts that biochemical diversification is among the least explored molecular mechanisms by which microtubules are adapted to specific cellular functions. This claim misrepresents the state of the field, given the substantial body of literature already addressing this topic. The title similarly overstates the findings by asserting that PTMs provide "unique recognition patterns," when in fact the data show that the majority of proteins tested are not detectably affected by tubulin source or PTM status. This mismatch between claims and results undermines confidence in the framing of the work.

We thank the referee for the critical assessment of our work. While we agree that we need to revise some of the wordings of our introduction, we maintain that our work is novel and unique as that we tested more than 50 proteins in parallel for their sensitivity to multiple tubulin PTMs, a work that in this form and breadth has not been done before. This is an important point in particular in the light of what the referee has themself said: "This claim misrepresents the state of the field, given the substantial body of literature already addressing this topic." – while we acknowledge this, the difficulty with the interpretation of these results is that they were obtained in different laboratories using different experimental approaches that can not necessarily be directly compared. The breakthrough we present here are therefore directly comparably data, which we now highlight in the abstract and the introduction.

We would also like to point out that the referee's criticism: "when in fact the data show that the majority of proteins tested are not detectably affected by tubulin source or PTM status" is a misinterpretation of our results. In fact, our observation that only about 50% of the tested MAPs show sensitivity to tubulin PTMs is a very important result, as the tubulin code concept would NOT be valid if all MAPs would be sensitive to PTMs. The idea of selectivity requires that PTMs can only be read by a subset of interactors, for which our work provides solid evidence. We have revised the wording to make sure this message is clearly stated from the beginning of the paper.

Likewise, the introduction states that "several labs including ours have shown that some microtubule interactors can directly sense tubulin modifications," as if the current work is about to add many more MAPs to this list. This is clearly not the case: most MAPs were found to be unaffected by PTMs, while those that were affected (e.g. CLIP-170) were mostly already known. Earlier work has also already addressed the EML proteins (e.g., PMID 35963242), yet this work is acknowledged only superficially. Although the discussion is somewhat more explicit about prior findings, the choice not to engage with them in the introduction creates an asymmetry that distorts the perceived novelty of the contribution.

We politely disagree with this point. Many of the MAPs we show here as sensitive to tubulin PTMs have not been shown before to be responsive to the tubulin code. In fact, a substantial subset of them was never investigated in cell-free approaches. Among the MAPs that, as the referee rightly pointed out, have been investigated before, often only one tubulin PTM was tested, while our work gives a comprehensive picture of their sensitivity to multiple tubulin PTMs. Among the MAPs we have tested here, no comparable data were so far available for SNPH_A, SNPH_C, TRIM46, MACF1(C1023), MAP1A, MAP1B, MAP2C, MAP4_X16, MAP4_X16, MTCL1, ATIP3, CFAP20, CKAP2, CKAP2L, DCX, GLFND1, GLFND2, MACF2(C1079), MAP6, MAP7, MAP7D1, MAP7D1, MAP7D2, MAP7D3, MAP8, MAP9, MAP10, PRC1, SPEF1 – in other words, 83% of the data shown here are novel.

To address the referee's point, we have now moved and substantially expanded parts of the Discussion into the Introduction to introduce the topic in a more explicit and detailed way.

The results themselves are, for the most part, not very compelling. No effect of acetylation is observed, and no difference is detected between brain tubulin with and without TUBB3.

The referee is absolutely right about this observation, but why is this not compelling? The fact that we see effects of polyglutamylation and tyrosination in the very same assay demonstrates that in the group of MAPs we are investigating none is sensitive to acetylation and TUBB3, which we think is a valuable result. We are aware that this can be related to the experimental conditions we use (stabilised microtubules), and we now discuss this in our revised manuscript.

The majority of MAPs that do show a difference follow the same trend, preferring tyrosinated over detyrosinated microtubules, which limits the mechanistic diversity of the findings.

As mentioned above, we have investigated a broad and unbiased selection of proteins here, therefore the fact that all of those that sense tyrosination prefer the tyrosinated state of tubulin is a result that we cannot influence. We also discuss this result in our manuscript and provide potential explanations for it.

Crucially, where clear effects are observed, they are not followed up functionally, leaving the biological significance of the selectivity differences entirely unexplored. The most interesting result, the differential behaviour of the EML proteins, is also the most problematic, as it is inconsistent with previously published data. Rather than investigating this discrepancy, the manuscript leaves it unresolved.

We agree with the referee that the different sensitivities of EMLs to different PTMs is one of the most exciting results of our work. However, in the context of this study, we did not follow it up as it would go beyond the scope.

As already discussed in the manuscript, possible explanation of the discrepancy between our results and the published work could be in different methods that have been used and that might have visualised kinetically different states of the MAP-microtubule interactions. We feel that we are in no position to directly determine the reason for this difference. Having said this, the literature contains many examples of divergent results, which will likely be resolved when deeper insight is gained in future work.

Thus, while the tubulin purification work is genuinely impressive, the biological insights derived from these preparations are limited, the most interesting finding is in tension with the existing literature without explanation, and the standard of scholarship is not at the level expected for [another journal]. While I hope the authors will improve their engagement with earlier work in a revised version, the reported findings would still be best suited for a more specialized journal.

We politely disagree with some of the claims made here: we have discussed differences with the literature, and while we will revise the paper to adapt the wording, we do not agree that our work does not provide novel insights and does not advance the field for reasons already discussed in our answers to the above points.

Reviewer #2 (Remarks to the Author):

Paper Summary

The paper presents an effective medium-throughput approach for determining the tubulin code preference of different MAPs. Tubulin purified from mouse brain under different conditions of tubulin PTM modifying enzyme knockdown is used to assess relative binding of 35 different MAPs in cell lysate that have been GFP-tagged. The study identifies 17 MAPs that display a clear binding preference for PTM specific tubulin, primarily focused on differences in tyrosination state of the tubulin, which is notably highly variable between brain WT and HeLA WT cells. The authors then further analyze the impact of PTMs on Syntaphilin and discuss the role of alternative splicing in establishing PTM preference, and of differences within the EML protein family. Through these tests, the authors can recognize specific domains that enable PTM recognition.

Major Points

1) Fig 1b. It appears that the TtlI1/Ttll7 knockdown sample is still polyglutamylated at some rate as well as an increase in acetylation. I would generally like to see this as the rate change from wild-type with some discussion of what percentage of tubulin you expect to contain the PTM based off of the appearance rate relative to tubulin amount used. Similarly, please show this for the in vitro detyrosinated HeLA tubulin used throughout the study.

We thank the referee for mentioning this point. Quantifying polyglutamylation using more precise methods than immuno blots have for long been a difficult task, in particular mass spectrometry was challenging due to the complex and highly acidic nature of the peptides carrying the polyglutamylation. This has now been overcome in a recent work in which the same tubulins used in the current study have been quantitatively analysed by top-down mass spectrometry (Gant MS, Magiera MM, Druart K, Dupré M, Genova M, Khanfir S, Matondo M, Bastin P, Janke C, Chamot-Rooke J (2026) Unlocking the activities of polyglutamylation enzymes in brain tubulin with top-down mass spectrometry. *ChemRxiv***:** https://doi.org/10.26434/chemrxiv.15005988/v15005981).

We now cite this paper and provide the quantitative measures found in this study, which clearly show that polyglutamylation on α-tubulin is lost after knockout of TTLL1, while β-tubulin glutamylation virtually entirely disappears after knockout of TTLL7. For the tyrosination / detyrosination: the specificity of the two antibodies, anti-tyrosinated (YL1/2) and anti-detyrosinated are such that the complete absence of any immunoreactivity in the two analysed samples clearly indicates the absence of detyrosination in the untreated Hela tubulin, and vice versa the absence of tyrosinated tubulin in the carboxy-peptidase A treated tubulin. We think this result is clear and does not require additional quantification, which is now explicitly stated in the text.

2) The recent work presented at BPS and ASCB from Yildiz and Nogales https://www.sciencedirect.com/science/article/pii/S0006349525023422?via=ihub showed the

EML recognition motif of tubulin PTMs through cryo-EM, is the identified domain consistent with the domain studied here?

We thank the referee for pointing this out. Currently, however, we have only the information from the conference abstract cited by the referee, we are therefore in no position to know the content of this work until it will be published. We can therefore not address this comment, though we think it would be great to compare our studies.

3) Has any sequence analysis been done on the splice-regulated domain identified in SNPH variants against other proteins? It would be of particular interest to see its sequence relation to other MAPs with tyrosination recognition differences.

This is an interesting question, which we now explored. We searched the domain that is generated by the splicing of SNPH_C that conferred sensitivity to polyglutamylation (both SNPH isoforms are sensitive to tyrosination, but only SNPH_C is sensitive to polyglutamylation) in the database, but we could not find any other mammalian protein containing this motif. We now mention this in the result section.

4) Fig 6. As is, this figure does not seem to provide much more information about the subject of the study. As a validation that the GFP-tagged proteins are still viable in the cell, it would be fine. As such, this should be moved to the beginning of the manuscript and possibly into a supplemental material.

At the same time, it would be interesting to see if there is a difference in co-localization between tubulin signal and the GFP-MAPs across Brain WT/HeLA WT/Ttll1+/Ttll7+ cell types. This should be tried and included with a discussion of PTMs in MDCK cells to get to a compelling final figure and thus ending of this manuscript.

We thank the referee for the thoughtful comment. After having discussed this point with the editor, we decided to leave the figure as final experimental figure because it is important to demonstrate that at least some of the in vitro data can be reproduced in cells with a simple overexpression of MAPs. This is unfortunately not the case for all of the here-measured PTM-induced changes of MAP-microtubule interactions, as some of the effects we measured in our in vitro assays induced rather gradual changes in binding affinities which are likely to be overwritten by the overexpression of MAPs in cells. We have tested proteins that were found to be sensitive to polyglutamylation but could not visualise the impact of polyglutamylation in cells, where these MAPs already bound to microtubules in the absence of polyglutamylation.

Minor Points

5) Please review grammar in the introduction section.

We have carefully revised the manuscript, and we hope that all grammar issues have been addressed.

6) Fig 1b. Please include the targeted PTM and tubulin isoform in the figure itself. Additionally, the source labeling has a box rendering error.

The dotted line was meant to connect the two grey boxes representing brain tubulin. Thanks to the reviewer's comment we realised that this was difficult to understand, and we have now changed the figure to make the annotation clearer. We have now also added the targeted PTM or tubulin isotype directly on the immuno blots to make it clearer for the reader.

7) Fig 5a – Please find a different way for labeling these domains that is more consistent with the canonical tubulin binding domain. Also, from this there is one clear difference in the carboxy-terminal domain from EML4, is there any significance in the rest of this domain for PTM recognition?

The labelling for the EML proteins followed our choices of cloning and should help the reader to visualise the constructs used in Fig. 5, EV3, EV4, EV5 and EV6. However, we appreciate that more detailed information is available on domains which we have now included in Fig. 5A and Fig. EV1.

We also include a new experiment (Fig. EV6) that demonstrates that the rest of the EML4 C-terminal domain has no impact on the binding preference of EML4, underpinning the notion that the PTM recognition of EML proteins is not assured by a single sequence domain.

8) You mentioned the dilution factors used for EML lysates, but not others. What were they and were they also robust across dilution factors?

The GFP-MAP lysates were diluted such that the GFP-MAP binding to the microtubule was not saturated, while the GFP signal was high enough for reliable quantification (Fig. 2B). In this regime, the precise concentration of GFP-MAPs in our assays had negligible impact on the final results. We now added additional dilution series for three other proteins used in this paper in previous Fig. S33 (now Appendix Fig. S29) to demonstrate that the observed binding preferences of several proteins can consistently be observed across different dilutions.

1st Revision - Editorial Decision 18th Aug 2026

Dear Carsten,

Thank you for submitting a revised version of your manuscript to The EMBO Journal. I have now gone through your responses to the reviewers' comments and find them generally reasonable. Therefore, there remain only a few editorial points that need to be addressed before I can extend the official acceptance of the manuscript:

1. Please note that it is The EMBO Journal policy for the transcript of the editorial process (containing referee reports and your response letters) to be published as an online supplement to each paper. If you should prefer removal of any referee-only figures included in the point-by-point response(s), e.g. because they may still be used for future publication or because they have been reproduced from published work by others, please do let us know immediately via response email.
More information is available here: https://www.embopress.org/transparent-process#Review_Process
2. Please update the order and the headings of the manuscript sections to: Abstract / Introduction / Results / Discussion / Methods / Data Availability / Acknowledgements / Disclosure and Competing Interests Statement / References / Figure Legends / Expanded View Figure Legends.
3. CRediT has replaced the traditional author contributions section because it offers a systematic, machine-readable author contributions format that allows for more effective research assessment. Please remove the Authors Contributions from the manuscript and use the free text boxes beneath each contributing author's name in our online submission system to add specific details on the author's contribution. More information is available in our guide to authors.
4. Please ensure that the figures are cited in sequential order. Currently, Fig 2A is called out before Fig 1, Appendix Fig S29 is called out before Appendix Fig S1; callouts are missing for Fig 3A-C.
5. Our data editors have asked to indicate the statistical test used for data analysis in the legends of figures 3B, EV3 B.
6. In the "References" section, please remove the DOIs for all published articles.
7. In the Appendix file, please add page numbers to the table of contents and correct the nomenclature in the figure legends to "Appendix Figure S1" etc.
8. Some of the immunofluorescence images and graphs are reused between the main figures and the Appendix figures. Please state this explicitly in the figure legends.
9. Papers published in The EMBO Journal are accompanied online by a 'Synopsis' to enhance discoverability of the manuscript. It consists of A) a short (1-2 sentences) summary of the findings and their significance, B) 3-4 bullet points highlighting key results (the highlights currently included in the manuscript can be used here) and C) a synopsis image that is 550x300 pixels large (width x height, jpeg or png format). You can either show a model or key data in the synopsis image. Please note that the image size is rather small and that text needs to be readable at the final size.

Please let me know if you have any questions regarding any of these points. You can use the link below to upload the revised files.

Thank you again for giving us the chance to consider your manuscript for The EMBO Journal. I look forward to receiving the final version.

With best wishes,

Ieva

Ieva Gailite, PhD
Senior Scientific Editor
The EMBO Journal
Meyerhofstrasse 1
69117 Heidelberg
i.gailite@embojournal.org

We realize that it is difficult to revise to a specific deadline. In the interest of protecting the conceptual advance provided by the work, we recommend a revision within 3 months (16th Nov 2026). Please discuss the revision progress ahead of this time with the editor if you require more time to complete the revisions.

The authors addresses the remaining editorial issues.

2nd Revision - Editorial Decision **27th Aug 2026**

Dear Carsten,

Thank you for incorporating the final formatting requests in the manuscript. I am now pleased to inform you that your manuscript has been accepted for publication. Congratulations on a nice study!

Before we forward your manuscript to our publishers, I would like to propose some edits in the synopsis text that you kindly provided, mainly to streamline it. I would also like to propose a couple of alternative versions of the title to increase its clarity. Finally, I have selected a short blurb that will accompany the title of your manuscript in our online table of contents. Please take a look at the proposed textual changes in the attached text file and let me know what you think.

Your manuscript will be processed for publication by EMBO Press. It will be copy edited and you will receive page proofs prior to publication. Please note that you will be contacted by Springer Nature Author Services to complete licensing and payment information.

You may qualify for financial assistance for your publication charges - either via a Springer Nature fully open access agreement or an EMBO initiative. Check your eligibility: https://link.springer.com/journal/44318/how-to-publish-with-us

Should you be planning a Press Release on your article, please notify the production team via this form in order to coordinate publication and release dates: https://forms.cloud.microsoft/e/eH4A8JVP6g

Once I have received your input on the textual edits, I will be happy to forward your manuscript to our publishers for typesetting.

Thank you for this interesting contribution to The EMBO Journal!

Best wishes,

Ieva

Ieva Gailite, PhD
Senior Scientific Editor
The EMBO Journal
Meyerhofstrasse 1
D-69117 Heidelberg
i.gailite@embojournal.org

*****
Please note that it is The EMBO Journal policy for the transcript of the editorial process (containing referee reports and your response letters) to be published as an online supplement to each paper. If you should prefer removal of any referee-only figures included in the point-by-point response(s), e.g. because they may still be used for future publication or because they have been reproduced from published work by others, please do let us know immediately via response email.
More information is available here: https://link.springer.com/partners/embo-press/editorial-policies#Peer%20review

EMBO Press Author Checklist

Corresponding Author Name: Carsten Janke	
Journal Submitted to: EMBO Journal	
Manuscript Number: EMBOJ-2026-124886-T	

USEFUL LINKS FOR COMPLETING THIS FORM
The EMBO Journal - Author Guidelines
EMBO Reports - Author Guidelines
Molecular Systems Biology - Author Guidelines
EMBO Molecular Medicine - Author Guidelines

Reporting Checklist for Life Science Articles (updated January
This checklist is adapted from Materials Design Analysis Reporting (MDAR) Checklist for Authors. MDAR establishes a minimum set of requirements in transparent reporting in the life sciences (see Statement of Task: 10.31222/osf.io/9sm4x). Please follow the journal's guidelines in preparing your
Please note that a copy of this checklist will be published alongside your article.

Abridged guidelines for figures
1. Data
The data shown in figures should satisfy the following conditions:
- the data were obtained and processed according to the field's best practice and are presented to reflect the results of the experiments in an accurate and unbiased manner.
- ideally, figure panels should include only measurements that are directly comparable to each other and obtained with the same assay.
- plots include clearly labeled error bars for independent experiments and sample sizes. Unless justified, error bars should not be shown for technical
- if n<5, the individual data points from each experiment should be plotted. Any statistical test employed should be justified.
- Source Data should be included to report the data underlying figures according to the guidelines set out in the authorship guidelines on Data

2. Captions
Each figure caption should contain the following information, for each panel where they are relevant:
- a specification of the experimental system investigated (eg cell line, species name).
- the assay(s) and method(s) used to carry out the reported observations and measurements.
- an explicit mention of the biological and chemical entity(ies) that are being measured.
- an explicit mention of the biological and chemical entity(ies) that are altered/varied/perturbed in a controlled manner.
- the exact sample size (n) for each experimental group/condition, given as a number, not a range;
- a description of the sample collection allowing the reader to understand whether the samples represent technical or biological replicates (including how many animals, litters, cultures, etc.).
- a statement of how many times the experiment shown was independently replicated in the laboratory.
- definitions of statistical methods and measures:
  - common tests, such as t-test (please specify whether paired vs. unpaired), simple χ^2 tests, Wilcoxon and Mann-Whitney tests, can be unambiguously identified by name only, but more complex techniques should be described in the methods section;
  - are tests one-sided or two-sided?
  - are there adjustments for multiple comparisons?
  - exact statistical test results, e.g., P values = x but not P values < x;
  - definition of 'center values' as median or average;
  - definition of error bars as s.d. or s.e.m.

Please complete ALL of the questions below.
Select "Not Applicable" only when the requested information is not relevant for your study.

Materials

Newly Created Materials	Information included in the manuscript?	In which section is the information available? (Reagents and Tools Table, Materials and Methods, Figures, Data Availability Section)
New materials and reagents need to be available; do any restrictions apply?	Yes	Reagents and Tools table, Materials and methods, Tables S1 - S4

Antibodies	Information included in the manuscript?	In which section is the information available? (Reagents and Tools Table, Materials and Methods, Figures, Data Availability Section)
For **antibodies** provide the following information:<br>- Commercial antibodies: RRID (if possible) or supplier name, catalogue number and or/clone number<br>- Non-commercial: RRID or citation	Yes	Reagents and Tools table, Materials and methods, Tables S1 - S4

DNA and RNA sequences	Information included in the manuscript?	In which section is the information available? (Reagents and Tools Table, Materials and Methods, Figures, Data Availability Section)
**Short novel DNA or RNA including primers, probes**: provide the sequences.	Yes	Reagents and Tools table, Materials and methods, Tables S1; S2

Cell materials	Information included in the manuscript?	In which section is the information available? (Reagents and Tools Table, Materials and Methods, Figures, Data Availability Section)
**Cell lines:** Provide species information, strain. Provide accession number in repository **OR** supplier name, catalog number, clone number, and/**OR** RRID.	Yes	Reagents and Tools table, Materials and methods
**Primary cultures:** Provide species, strain, sex of origin, genetic modification status.	Not Applicable	
Report if the cell lines were recently **authenticated** (e.g., by STR profiling) and tested for mycoplasma contamination.	Not Applicable	

Experimental animals	Information included in the manuscript?	In which section is the information available? (Reagents and Tools Table, Materials and Methods, Figures, Data Availability Section)
**Laboratory animals or Model organisms:** Provide species, strain, sex, age, genetic modification status. Provide accession number in repository **OR** supplier name, catalog number, clone number, **OR** RRID.	Yes	Reagents and Tools table, Materials and methods
**Animal observed in or captured from the field:** Provide species, sex, and age where possible.	Not Applicable	
Please detail **housing and husbandry conditions**.	Yes	Materials and methods

Plants and microbes	Information included in the manuscript?	In which section is the information available? (Reagents and Tools Table, Materials and Methods, Figures, Data Availability Section)
**Plants:** provide species and strain, ecotype and cultivar where relevant, unique accession number if available, and source (including location for collected wild specimens).	Not Applicable	
**Microbes:** provide species and strain, unique accession number if available, and source.	Not Applicable	

Human research participants	Information included in the manuscript?	In which section is the information available? (Reagents and Tools Table, Materials and Methods, Figures, Data Availability Section)
If collected and within the bounds of privacy constraints report on age, sex and gender or ethnicity for all study participants.	Not Applicable	

Core facilities	Information included in the manuscript?	In which section is the information available? (Reagents and Tools Table, Materials and Methods, Figures, Data Availability Section)
If your work benefited from core facilities, was their service mentioned in the acknowledgments section?	Yes	Reagents and Tools table, Acknowledgement

Design

Study protocol	Information included in the manuscript?	In which section is the information available? (Reagents and Tools Table, Materials and Methods, Figures, Data Availability Section)
If study protocol has been **pre-registered, provide DOI in the manuscript**. For clinical trials, provide the trial registration number **OR** cite DOI.	Not Applicable	
Report the **clinical trial registration number** (at ClinicalTrials.gov or equivalent), where applicable.	Not Applicable	

Laboratory protocol	Information included in the manuscript?	In which section is the information available? (Reagents and Tools Table, Materials and Methods, Figures, Data Availability Section)
Provide DOI OR other citation details if **external detailed step-by-step protocols** are available.	Yes	Materials and Methods, citation of Jijumon et al. 2024 (DOI: 10.1002/cpz1.1070)

Experimental study design and statistics	Information included in the manuscript?	In which section is the information available? (Reagents and Tools Table, Materials and Methods, Figures, Data Availability Section)
Include a statement about **sample size** estimate even if no statistical methods were used.	Not Applicable	
Were any steps taken to minimize the effects of subjective bias when allocating animals/samples to treatment (e.g. **randomization procedure**)? If yes, have they been described?	Not Applicable	
Include a statement about **blinding** even if no blinding was done.	Not Applicable	
Describe **inclusion/exclusion criteria** if samples or animals were excluded from the analysis. Were the criteria pre-established?<br>If sample or data points were omitted from analysis, report if this was due to attrition or intentional exclusion and provide justification.	Not Applicable	Animals were only used for tubulin purification
For every figure, are **statistical tests** justified as appropriate? Do the data meet the assumptions of the tests (e.g., normal distribution)? Describe any methods used to assess it. Is there an estimate of variation within each group of data? Is the variance similar between the groups that are being statistically compared?	Yes	Figure legends, Appendix supplementary figure legends, Materials and Methods

Sample definition and in-laboratory replication	Information included in the manuscript?	In which section is the information available? (Reagents and Tools Table, Materials and Methods, Figures, Data Availability Section)
In the figure legends: state number of times the experiment was **replicated** in laboratory.	Yes	Figure legends, Appendix supplementary figure legends, Materials and Methods
In the figure legends: define whether data describe **technical or biological replicates**.	Yes	Figure legends, Appendix supplementary figure legends, Materials and Methods

Ethics

Ethics	Information included in the manuscript?	In which section is the information available? (Reagents and Tools Table, Materials and Methods, Figures, Data Availability Section)
Studies involving **human participants**: State details of **authority granting ethics approval** (IRB or equivalent committee(s), provide reference number for approval.	Not Applicable	
Studies involving **human participants**: Include a statement confirming that **informed consent** was obtained from all subjects and that the experiments conformed to the principles set out in the WMA Declaration of Helsinki and the Department of Health and Human Services Belmont Report.	Not Applicable	
Studies involving **human participants:** For publication of **patient photos**, include a statement confirming that consent to publish was obtained.	Not Applicable	
Studies involving experimental **animals**: State details of **authority granting ethics approval** (IRB or equivalent committee(s), provide reference number for approval. Include a statement of compliance with ethical regulations.	Yes	Materials and Methods section
Studies involving **specimen and field samples:** State if relevant **permits** obtained, provide details of authority approving study; if none were required, explain why.	Not Applicable	

Dual Use Research of Concern (DURC)	Information included in the manuscript?	In which section is the information available? (Reagents and Tools Table, Materials and Methods, Figures, Data Availability Section)
Could your study fall under dual use research restrictions? Please check biosecurity documents and list of **select agents and toxins** (CDC): https://www.selectagents.gov/sat/list.htm	Not Applicable	
If you used a select agent, is the security level of the lab appropriate and reported in the manuscript?	Not Applicable	
If a study is subject to dual use research of concern regulations, is the name of the **authority granting approval and reference number** for the regulatory approval provided in the manuscript?	Not Applicable	

Reporting

The MDAR framework recommends adoption of discipline-specific guidelines, established and endorsed through community initiatives. Journals have their own policy about requiring specific guidelines and recommendations to complement MDAR.

Adherence to community standards	Information included in the manuscript?	In which section is the information available? (Reagents and Tools Table, Materials and Methods, Figures, Data Availability Section)
State if relevant guidelines or checklists (e.g., **ICMJE, MIBBI, ARRIVE, PRISMA**) have been followed or provided.	Not Applicable	
For **tumor marker prognostic studies,** we recommend that you follow the **REMARK** reporting guidelines (see link list at top right). See author guidelines, under 'Reporting Guidelines'. Please confirm you have followed these guidelines.	Not Applicable	
For **phase II and III randomized controlled trials**, please refer to the **CONSORT** flow diagram (see link list at top right) and submit the CONSORT checklist (see link list at top right) with your submission. See author guidelines, under 'Reporting Guidelines'. Please confirm you have submitted this list.	Not Applicable	

Data Availability

Data availability	Information included in the manuscript?	In which section is the information available? (Reagents and Tools Table, Materials and Methods, Figures, Data Availability Section)
Have **primary datasets** been deposited according to the journal's guidelines (see 'Data Deposition' section) and the respective accession numbers provided in the Data Availability Section?	Yes	
Were **human clinical and genomic datasets** deposited in a public access-controlled repository in accordance to ethical obligations to the patients and to the applicable consent agreement?	Not Applicable	
Are **computational models** that are central and integral to a study available without restrictions in a machine-readable form? Were the relevant accession numbers or links provided?	Not Applicable	
If publicly available data were reused, provide the respective **data citations in the reference list**.	Not Applicable	
